# Advances in Dendrimer-Based Anti-Infective Systems: In Vivo Insights and Perspectives

**DOI:** 10.3390/pharmaceutics18070851

**Published:** 2026-07-13

**Authors:** Charlotte Aparici, Kevin Antraygues, Vania Bernardes-Génisson, Manuel S. Rodriguez, Cédric-Olivier Turrin, Valérie Maraval, Anne-Marie Caminade

**Affiliations:** 1 Univ Toulouse, CNRS, LCC, Toulouse, France; charlotte.aparici@lcc-toulouse.fr (C.A.); kevin.antraygues@lcc-toulouse.fr (K.A.); vania.bernardes-genisson@lcc-toulouse.fr (V.B.-G.); manuel.rodriguez@lcc-toulouse.fr (M.S.R.); valerie.maraval@lcc-toulouse.fr (V.M.); 2 Laboratoire de Chimie de Coordination, 205 Route de Narbonne, 31077 Toulouse CEDEX 4, France; 3 IMD-Pharma, 205 Route de Narbonne, 31077 Toulouse CEDEX 4, France

**Keywords:** dendrimer, antimicrobial resistance, anti-infective therapy, in vivo, bacterial vaginosis, wound infection, tuberculosis, antiviral

## Abstract

The rise of antimicrobial resistance and the persistence of difficult-to-treat infections have stimulated interest in new strategies to overcome these problems. Among these strategies, dendrimers, which are highly branched monodisperse macromolecules, have emerged as innovative antimicrobial and anti-infective platforms. Dendrimers can act as intrinsic antimicrobial agents through multivalent interactions or membrane disruption or serve as nanocarriers for antibiotics, antiviral agents, antibiofilm compounds, gas-releasing active molecules, or photosensitizers. This review analyzes dendrimer-based anti-infective systems for which in vivo or clinical evaluation has been reported. The literature covers diverse platforms, including PAMAM, poly(L-lysine), peptide, carbosilane, phosphorhydrazone, polyglycerol, polyester, and other dendritic architectures. The available evidence includes infected animal models, pharmacokinetic and biodistribution studies, local tolerance studies, and clinical trials. PAMAM systems are the most extensively explored preclinically, whereas poly(L-lysine) dendrimer astodrimer/SPL7013 remains the most clinically advanced example. Overall, dendrimers provide a chemically tunable and biologically versatile approach to anti-infective research, but the current evidence remains heterogeneous. Direct comparison across studies is limited by differences in dendrimer scaffold, generation, surface chemistry, formulation, pathogen, infection model, administration route, dosing regimen, and biological endpoint. Future development will require better-defined in vivo models, more systematic safety and biodistribution studies, clearer structure–activity relationships, and stronger links between in vitro activity and clinically relevant efficacy.

## 1. Introduction

Antibiotics transformed modern medicine following Alexander Fleming’s serendipitous discovery of penicillin in 1928 [1]. The subsequent expansion of the antibiotic arsenal rendered previously fatal infections treatable, saving millions of lives [2]. However, the rise of antibiotic-resistant bacterial strains, particularly those exhibiting multidrug resistance (MDR) [3], i.e., resistance to at least three different classes of antimicrobials [4,5], has undermined this progress, necessitating innovative strategies to combat antimicrobial resistance [6,7,8].

**Figure 1:**
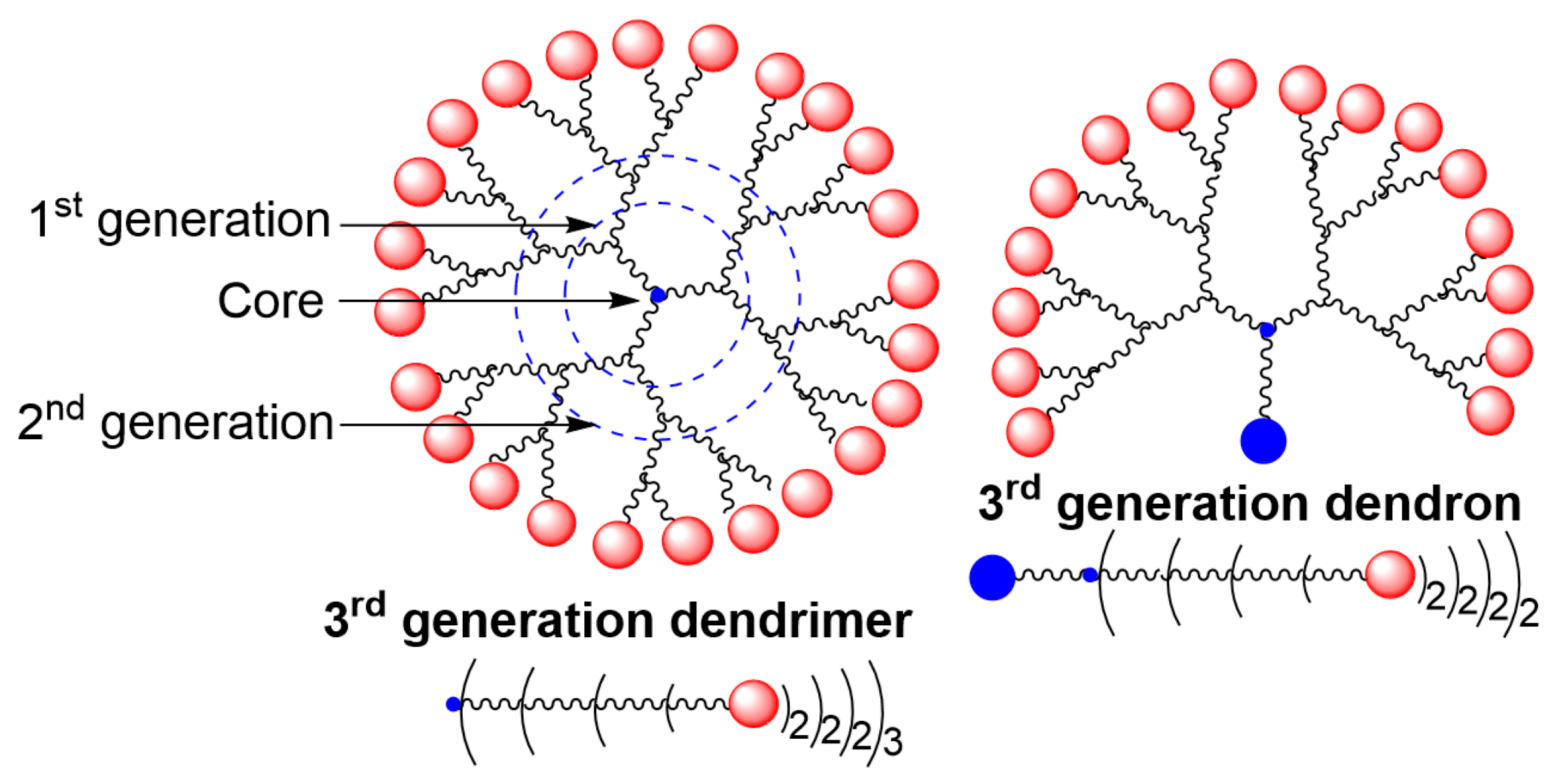
Schematization of dendrimers and dendrons (dendritic wedges). Both are represented as the full structure and the abbreviated chemical structure, in a linear form, with parentheses after each branching point, which define the number of generations (red points: surface functions; blue points: core).

Dendrimers [9,10,11,12,13], highly branched and monodisperse macromolecules, have emerged as promising tools in this fight. Dendrimers, as well as dendrons, which are dendritic wedges [14], offer both intrinsic antimicrobial activity and the ability to enhance the efficacy of existing drugs [15,16]. Their unique architecture—a central core, branching units, and functional surface groups—enables precise modification and targeted delivery, making them attractive for in vivo applications (Figure 1). Certain dendrimers, such as cationic PAMAM (polyamidoamine) [17] and peptide-based dendrimers [18,19,20], exhibit intrinsic antibacterial and antitoxin effects [21]. Their positive charge can disrupt bacterial membranes and induce cell lysis. This membrane-targeting mode of action may reduce the likelihood of target-specific resistance compared to single-enzyme inhibitors, although bacterial adaptation to cationic membrane-active compounds, including quaternary ammonium antiseptics and disinfectants, can occur through mechanisms such as cell-envelope remodeling, changes in membrane structure or function, efflux pumps, and biofilm formation [22]. Beyond direct action, dendrimers and dendrons can encapsulate or be conjugated with antimicrobial agents, enhancing their solubility, stability, and bioavailability. Advanced dendrimer designs now incorporate environmental responsiveness (e.g., pH, redox conditions), enabling targeted drug release at infection sites and minimizing off-target effects. Dendritic materials appear as an innovative tool in the clinical management of infectious diseases and many other health issues [23].

While numerous studies and reviews have described the antimicrobial potential of dendritic molecules [24,25,26,27], most have focused primarily on chemical design, structure–activity relationships, and in vitro activity. However, moving from promising in vitro results to potential clinical development requires in vivo studies, which provide essential information on efficacy, tolerability, biodistribution, administration routes, and biological outcomes in complex organisms [28]. These data remain scattered across heterogeneous studies involving different dendrimer scaffolds, pathogens, infection models, dosing regimens, and endpoints, making direct comparison difficult. This review primarily focuses on dendrimer-based antimicrobial systems evaluated in vivo, including dendrimers with intrinsic antibacterial or antibiofilm activity and dendrimer-based carriers of antimicrobial agents. Antiviral examples are included when they involve dendritic systems developed as anti-infective agents, particularly when in vivo or clinical data are available. Studies mainly addressing wound healing, tissue regeneration, or inflammation are discussed only when they are directly associated with infection control, antimicrobial activity, or the delivery of anti-infective agents.

The in vivo models discussed in this review should not be interpreted as providing equivalent levels of translational evidence. At the same time, their value cannot be ranked solely according to the animal species used. As summarized by Box’s aphorism that “all models are wrong, but some are useful” [29], each model should be considered according to its fitness for the biological question being asked. Invertebrate models such as *Caenorhabditis elegans* and *Galleria mellonella* can be informative for early antimicrobial screening, host survival, and preliminary toxicity assessment. Zebrafish larvae may provide useful information on infection-site accumulation and whole-organism biodistribution in a lower vertebrate model. Rodent models are more relevant for evaluating mammalian infection biology, local and systemic efficacy, tissue distribution, and route-dependent pharmacology. Non-human primate studies provide the closest translational evidence but remain available for only a small number of dendrimer systems. Finally, clinical trials are the first evaluation in humans, performed with a limited sample of volunteers. Therefore, rather than ranking studies only by species, this review considers whether the model is fit for the intended biological question, including infection site, pathogen, immune context, route of administration, dosing regimen, comparator, and endpoint.

This review is organized according to dendrimer family, including PAMAM dendrimers and dendrons, poly(L-lysine) and other peptide dendrimers, and miscellaneous dendritic architectures. For each family, we discuss both dendrimers acting as antimicrobial agents themselves and systems used to deliver or potentiate antimicrobial compounds.

## 2. Literature Search Strategy and Scope of the Review

This review focuses on dendrimer-based anti-infective systems for which at least one in vivo or clinical evaluation has been reported. Literature searches were performed using Web of Science and to a lesser extent SciFinder, using combinations of the following keywords: dendrimer, dendron, anti-infective, in vivo. The search covered the literature available up to April 2026.

Studies were included when they described dendrimer or dendron systems with anti-infective relevance and included at least one in vivo or clinical evaluation. In vivo evidence was defined broadly as data obtained in living organisms or human subjects, including infected animal models, clinical trials, pharmacokinetic or biodistribution studies, local tolerance studies, or in vivo delivery studies when the dendrimer system was designed for anti-infective use. In vitro data were discussed when they were necessary to introduce the antimicrobial activity, mechanism of action, drug-loading strategy, antibiofilm effect, cytotoxicity, or release behavior of systems subsequently evaluated in vivo. Dendrimer systems supported exclusively by in vitro data were not considered within the main scope of this review.

Antiviral examples were included when they involved dendrimer-based anti-infective systems evaluated in vivo or clinically, particularly when they illustrated clinically advanced dendrimer platforms. Studies mainly addressing wound healing, tissue regeneration, or inflammation were included only when they were directly associated with infection control, antimicrobial activity, local tolerance, or delivery of anti-infective agents. This review was not designed as a systematic review, a regulatory assessment, or a pharmaceutical development review. Its objective is to provide a chemistry-guided overview of dendrimer-based antimicrobial and anti-infective systems evaluated in vivo, while highlighting the main biological and translational limitations emerging from these studies.

## 3. Poly(amidoamine) (PAMAM) Dendrimers and Dendrons

**Figure 2:**
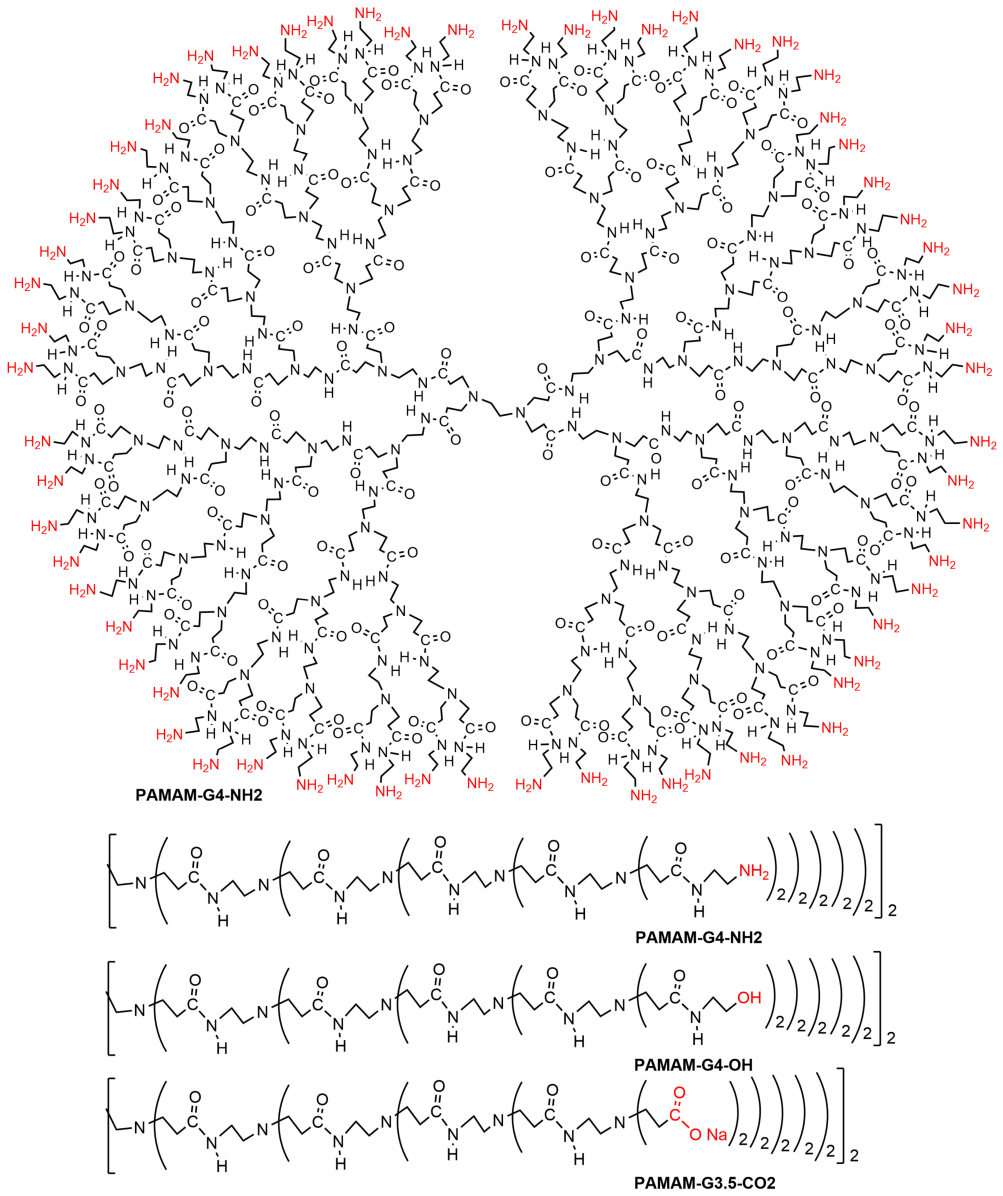
Full chemical structure and linear representation of **PAMAM-G4-NH2** and linear representation of other PAMAM dendrimers. Surface functions in red.

Poly(amidoamine) (PAMAM) dendrimers, pioneered by D.A. Tomalia et al. [30], are the most widely used type of dendrimers. They possess either carboxylic acids or primary amines as terminal functions, depending on the synthesis step considered, or can be modified to alcohols. Both the full structure and the corresponding linear representation for **PAMAM-G4-NH2** are shown in Figure 2, together with the linear structures of **PAMAM-G4-OH** and **PAMAM-G3.5-CO2**. The relatively large number of PAMAM examples discussed below reflects their predominant place in the dendrimer literature and, more specifically, their frequent use in in vivo anti-infective studies.

### 3.1. PAMAM Dendrimers and Dendrons as Antimicrobial Drugs by Themselves

The three dendrimers shown in Figure 2 were tested as antimicrobial agents against *Escherichia coli*. **PAMAM-G4-NH2** showed high antibacterial activity, with an IC_50_ value (half maximal inhibitory concentration, corresponding to the concentration of dendrimer necessary to kill 50% of the bacteria) of 3.8 μg·mL^−1^, but was toxic to a human cervical epithelial cell line above 10 μg·mL^−1^. On the contrary, **PAMAM-G4-OH** was non-cytotoxic up to 1 mg·mL^−1^, while exhibiting good antibacterial activity, inhibiting *E. coli* growth at concentrations as low as 3.13 mg·mL^−1^. Thus, this dendrimer was used to treat pregnant guinea pigs with intra-cervical *E. coli* infection (model of chorioamnionitis). The treatment consisted of topically applying the **PAMAM-G4-OH** dendrimer to the cervical endometrium of guinea pigs at a dose of 625 μg·kg^−1^. The amniotic fluid samples from different fetuses were collected after 48 h and did not show any bacterial growth (0%), unlike untreated animals, for which 57.1% of the amniotic fluid samples from different fetuses tested positive for bacterial growth. The proposed mechanism of action involved binding of **PAMAM-G4-OH** via hydrogen bonds to the hydrophilic O-antigens of the outer membrane of *E. coli* [31]. This pregnancy-associated infection model is relevant because it evaluates the ability of locally administered **PAMAM-G4-OH** to prevent bacterial growth in the amniotic environment. However, in such a gestational setting, antibacterial efficacy alone is not sufficient to support translation. Placental transfer, fetal exposure, reproductive safety, and long-term developmental outcomes should be specifically investigated in future studies.

**Figure 3:**
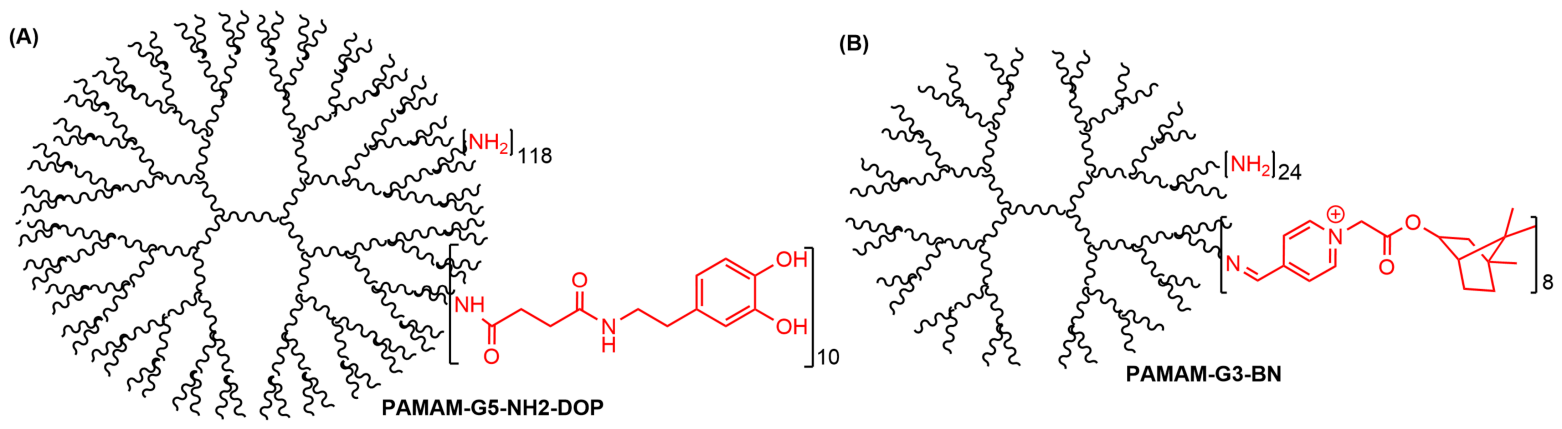
(**A**) **PAMAM-G5-NH2** partly covalently functionalized by reaction with a dopamine derivative. (**B**) **PAMAM-G3-NH2** partly covalently functionalized with borneol.

In addition to the intrinsic activity of native PAMAM dendrimers, they were also partly functionalized on their surface with different types of drug-related molecules. **PAMAM-G5-NH2** was partly functionalized with dopamine attached to the dendrimer via a succinyl linker (10 equiv. per PAMAM molecule), affording **PAMAM-G5-NH2-DOP** (Figure 3A). Dopamine is a derivative of catechol 3,4-dihydroxyphenylalanine, which is contained in adhesion proteins secreted by mussels. The resulting dendrimer adhered strongly to titanium alloy surfaces, which displayed antimicrobial and osteogenic activities. The same properties were observed in vivo for bone implants in rats, significantly enhancing the long-term stability of implants [32].

A very recent example of **PAMAM-G3-NH2** partial functionalization was carried out with a derivative of borneol (a bicyclic monoterpene extracted from medicinal plants that can interact with bacterial surfaces), affording **PAMAM-G3-BN** (Figure 3B). This dendrimer was tested first against the oral bacteria *Streptococcus mutans* and *Streptococcus sanguinis*. **PAMAM-G3-BN** reduced bacterial adhesion and viability, neutralized surface charge, and affected the integrity of the bacterial membrane, thus resulting in almost complete disintegration of biofilms. Male Sprague–Dawley rats (8 weeks) received human pre-demineralized enamel blocks (~3 × 3 × 1 mm) fixed to the maxillary molars, then were inoculated with *S. mutans* twice a day for 3 days, to establish a cariogenic environment. **PAMAM-G3-BN** (50 µL) was applied topically twice a day for 3 weeks. The shallowest lesion depth, the greatest remineralization performance, and tunable bactericidal activity were observed when using **PAMAM-G3-BN**. This dendrimer exhibited both antibacterial and enamel-preserving effects [33].

**Scheme 1:**
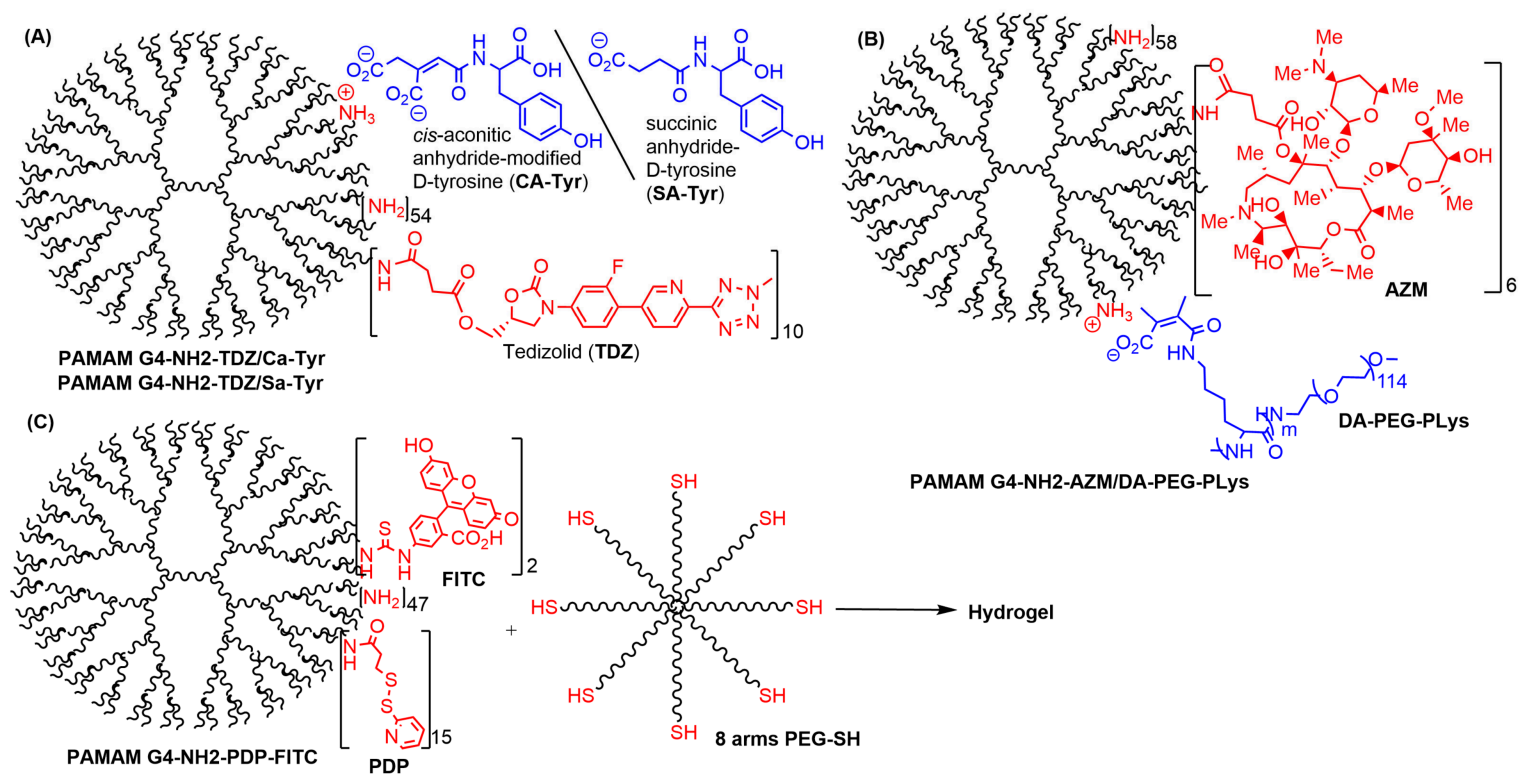
(**A**) **PAMAM G4-NH2** dendrimer partly covalently functionalized with tedizolid and interacting with **CA-Tyr** or **SA-Tyr**. (**B**) PAMAM dendrimer covalently functionalized with azithromycin and interacting with 2,3-dimethyl maleic anhydride modified by poly(ethylene glycol)-block-polylysine. (**C**) Synthesis of a hydrogel by thiol-disulfide exchange between partly functionalized PAMAM and a dendritic PEG.

**PAMAM-G4-NH2** was partly covalently functionalized with the antibiotic tedizolid (**TDZ**) and interacted electrostatically with either *cis*-aconitic anhydride-modified D-tyrosine (**CA-Tyr**) or succinic anhydride-D-tyrosine (**SA-Tyr**), affording **PAMAM-G4-NH2-TDZ/CA-Tyr** or **PAMAM-G4-NH2-TDZ/SA-Tyr** particles able to penetrate biofilms (Scheme 1A). **TDZ** is a drug approved for the treatment of acute bacterial skin and skin structure infection caused by Gram-positive bacteria, but it is inactive against biofilms due to its poor penetration ability. D-amino acids were shown to interfere with biofilm matrix protein synthesis, thus preventing biofilm development. In an acidic environment, the immediate release of D-Tyr damaged the matrix of the biofilm, enabling the penetration of **PAMAM-G4-NH2-TDZ**. In response to lipase, sustained release of **TDZ** was observed for a long time, leading to the biofilm eradication. These concepts were then applied in vivo against methicillin-resistant *Staphylococcus aureus* (MRSA)-induced subcutaneous abscess in mice, using dendritic compounds labelled with a few Cy-5 dyes. After intravenous (i.v.) injection, the dendrimer/**CA-Tyr** electrostatic assembly appeared in the subcutaneous abscess within 1 h and remained there for more than 24 h, as shown by fluorescence. This formulation induced the disappearance of 99% of bacteria, which were killed by the treatment. On the contrary, the fluorescence of the dendrimer/**SA-Tyr** electrostatic assembly labelled with Cy-5 was very weak at the infected site and disappeared after 12 h [34]. This example illustrates the pharmacological interest of combining biofilm matrix disruption, improved penetration into infected tissue, and antibiotic delivery within a single dendrimer-based formulation. However, its translational interpretation remains limited by the complexity of the electrostatic assembly, the absence of long-term toxicity data, and the lack of direct comparison with clinically used anti-MRSA regimens.

**PAMAM G4-NH2** was partly covalently functionalized with an average of six azithromycin molecules (**AZM**) per dendrimer and interacted electrostatically with 2,3-dimethyl maleic anhydride (**DA**) modified by poly(ethylene glycol)-block-polylysine polymers (**PEG-PLys**) (Scheme 1B). The resulting electrostatically associated **PAMAM-G4-NH2-AZM/DA-PEG-PLys** can be disassembled at acidic pH, in particular at pH 6.0, which is typical in biofilm microenvironment. The released **PAMAM-G4-NH2-AZM** penetrated inside biofilms and increased internalization of **AZM**, compared to free **AZM**. Furthermore, this dendrimer effectively alleviated the occurrence of **AZM** resistance. This process was used in mice suffering from chronic *Pseudomonas aeruginosa* infection of the lungs. The level of inflammation factors was significantly reduced in treated mice, which was consistent with a decrease in bacterial burden [35].

**PAMAM G4-NH2** was also partly functionalized with thiopyridyl terminations by reaction with *N*-succinimidyl 3-(2-thiopyridyl)propionate (**SPDP**), affording a dendrimer having 49 NH_2_ and 15 NH-PDP. This dendrimer was then reacted with two equivalents of fluorescein isothiocyanate (FITC) and finally reacted with branched thiol-terminated PEG polymer (**8-arm-PEGSH**, 20 kDa), using a thiol-disulfide exchange to produce a hydrogel (Scheme 1C). This hydrogel was used to encapsulate amoxicillin, and the release was studied in vitro. Sustained release of the antibiotic and intrinsic antibacterial activity exhibited by the amine-terminated dendrimer were expected. The in vivo studies in pregnant guinea pig consisted of local intravaginal insertion of the hydrogel without the amoxicillin drug. Tissues were tolerant to the gel, and the gel was located on the superficial mucified epithelial layer [36]. The release of the antibiotic from the gels was evaluated in vitro and was shown to be slower (over 240 h) when a higher polymer concentration is used, due to the smaller pores formed in this case.

**Scheme 2:**
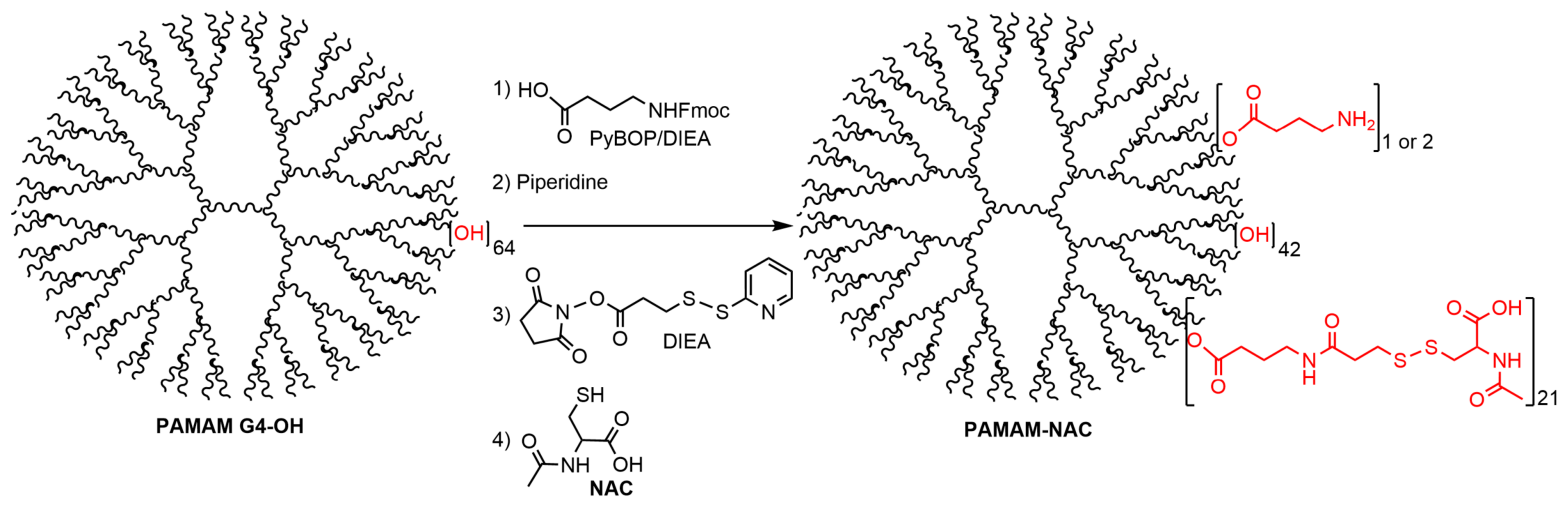
**PAMAM G4-OH** partly functionalized with *N*-acetyl cysteine (**NAC**). DIEA: diisopropylethylamine; PyBOP: benzotriazole-1-yloxytripyrrolidinophosphonium.

**PAMAM G4-OH** was partly functionalized with *N*-acetyl cysteine (**NAC**), known for attenuating neuroinflammation, to generate **PAMAM-NAC**, as shown in Scheme 2. This example illustrates a different type of clinical anti-infective relevance, in which the dendrimer platform is used to attenuate infection-associated inflammation and neurological injury rather than to directly inhibit viral replication. A disulfide linkage was used for grafting the **NAC**, which can be rapidly released in the brain in the presence of glutathione [37]. This dendrimer was tested in different animal models for treating inflammation, which is outside the scope of this review, but it was also used in a Phase 2a clinical trial as nanotherapy against severe COVID-19 infection. **PAMAM-NAC** was not directly used against the coronavirus; rather, it was used to attenuate inflammation and neurological injury. Twenty-four patients with severe COVID-19 infection received a single intravenous injection of **PAMAM-NAC** or of a placebo. Sixty days later, 3 of 7 patients receiving placebo and 14 of 17 patients treated with **PAMAM-NAC** were alive, and no-drug related adverse event was reported [38].

**Figure 4:**
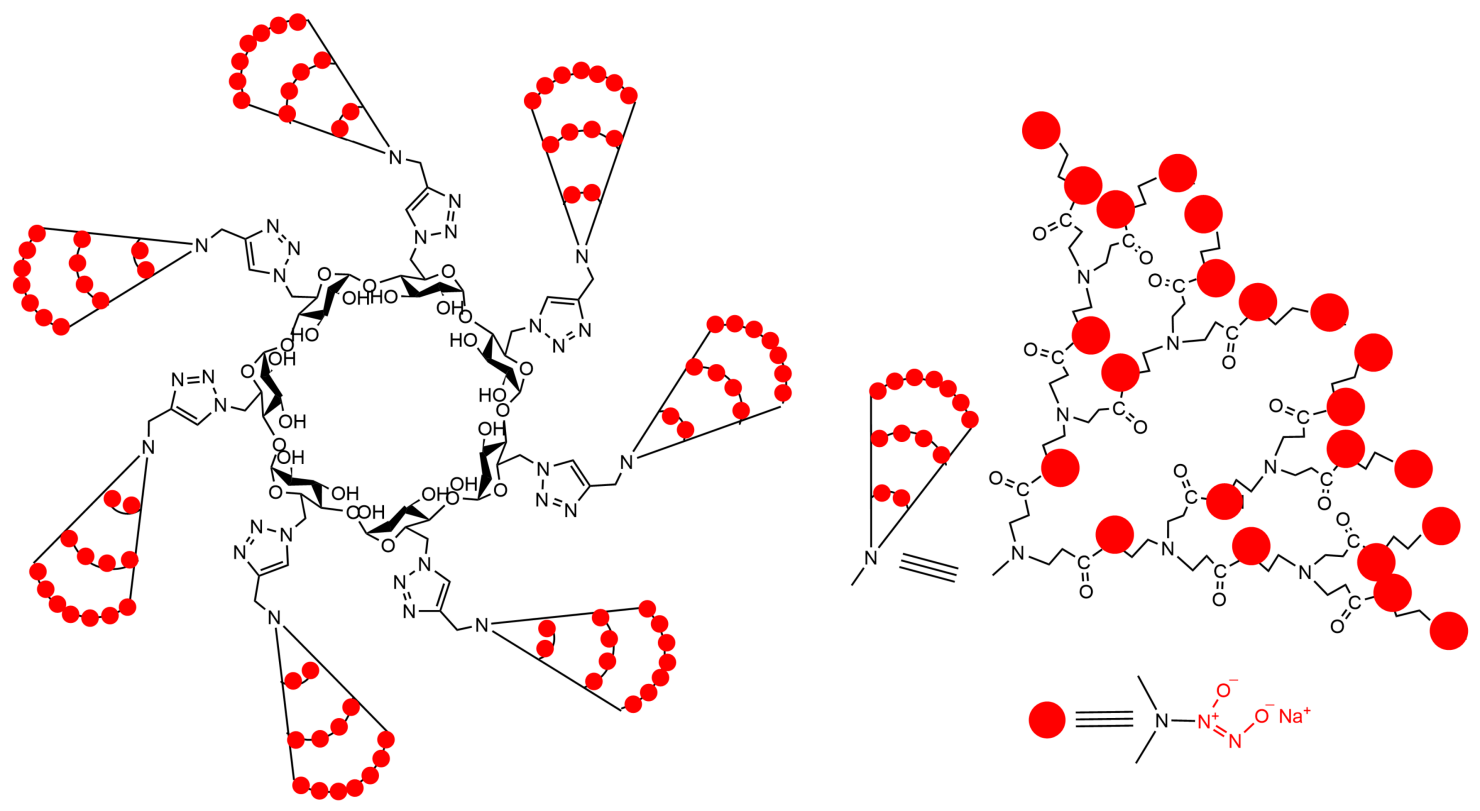
β-cyclodextrin as the core of the PAMAM dendrimer, and a proposed structure of its NO complex.

Another type of PAMAM dendrimer was synthesized by grafting seven PAMAM dendrons having an alkyne as the core to a β-cyclodextrin (CD) core functionalized with seven azides via a “click” reaction, which is frequently used for the synthesis and functionalization of dendrimers [39]. This dendrimer was then treated with nitric oxide (NO) under pressure (80 psi NO for 3 days) in the presence of MeOH/MeONa to react with the NH functions of the PAMAM dendrimer. A kind of NO dimer was thus created, with the aim of using the modified dendrimer as an NO reservoir, as NO is considered an interesting antibacterial agent [40]. The PAMAM dendrimer shown in Figure 4 was first shown to inhibit and disperse *Staphylococcus aureus* biofilms, thanks to the release of NO; then it was administered intranasally using a spray to rats inoculated with *S. aureus*. An excellent therapeutic effect was observed, with significant improvement of the symptoms [41].

**Figure 5:**
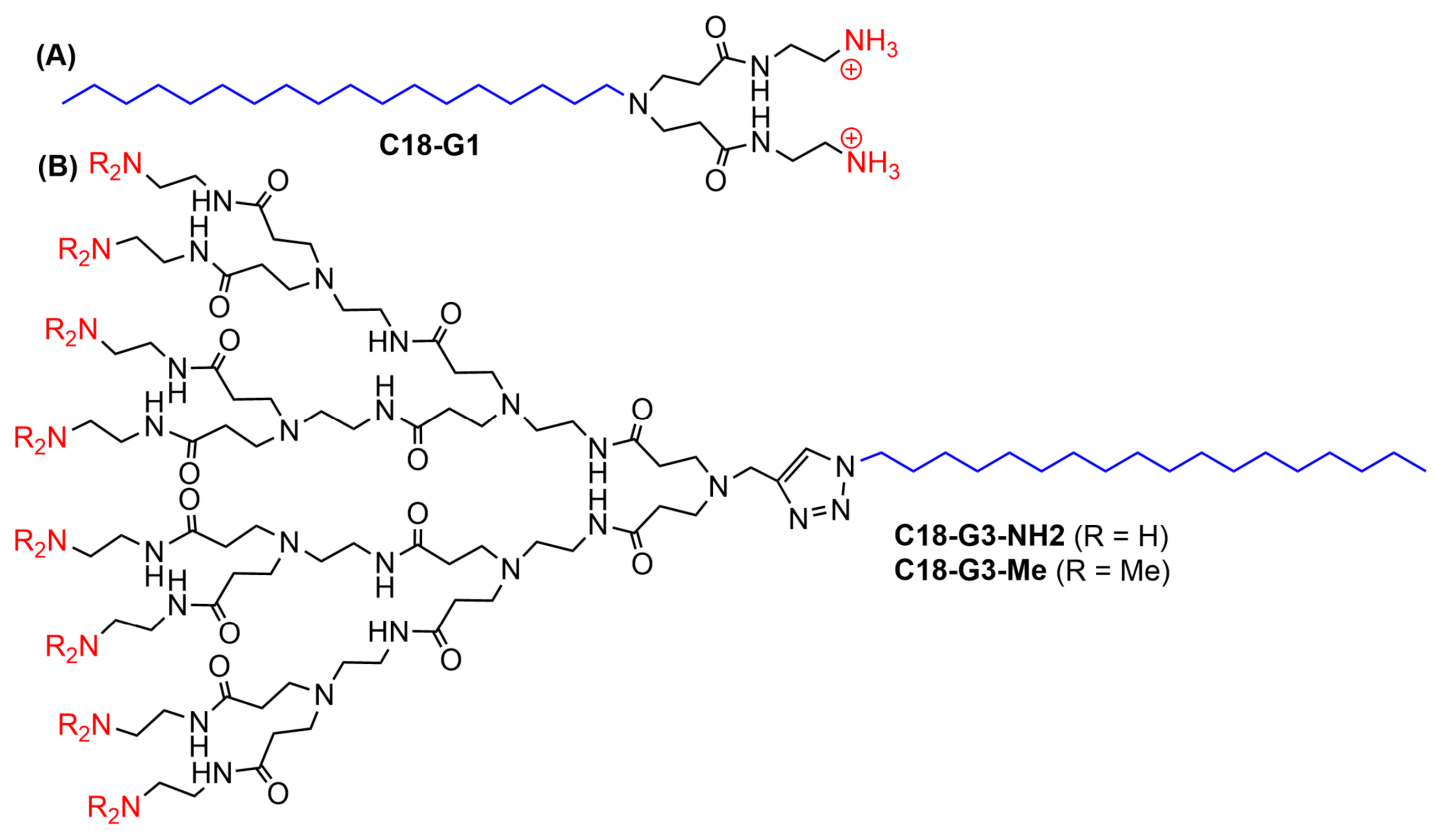
(**A**) PAMAM dendron **C18-G1**. (**B**) Two examples of third-generation PAMAM dendrons.

Different types of PAMAM dendrons were synthesized and identified as efficient antibacterial tools. A series of PAMAM dendrons having a hydrophobic alkyl chain (C6, C12, or C18) at the core and ammonium groups on the surface (2, or 4 NH_3_^+^) was synthesized and tested first on solutions of *S. aureus* and *E. coli*, including resistant strains. The best antibacterial activities were obtained using the compound with the C18 chain and 2 NH_3_^+^ (**C18-G1** Figure 5A). This compound was tested on healthy mice to evaluate its in vivo safety compared to that of methicillin, at a concentration of 0.6 mg·mL^−1^ in both cases. All the blood biochemical indices of mice from the dendron group were consistent with those from the methicillin groups, demonstrating the biocompatibility of dendron **C18-G1**. The in vivo antimicrobial efficacy of cationic dendron **C18-G1** was evaluated using a wound created on the back of the mice and infected with MRSA. An optimal wound healing rate reaching 65% on Day 7 was observed only with **C18-G1** [42].

A related compound with the same C18 alkyl chain at the core, but linked to the PAMAM part by a triazole ring, was synthesized by a “click” reaction. In a first example, the surface groups were eight primary amines, which can be easily protonated (compound **C18-G3-NH2** Figure 5B) [43]. It was shown that this compound binds to the bacterial membrane and that the hydrophobic tail of the dendron is inserted into this membrane, thereby inducing bacterial cell lysis. This dendron is thus mimicking the antibacterial activity of amphiphilic antimicrobial peptides. Dendron **C18-G3-NH2** was tested in vivo using mouse models of human infectious diseases (acute pneumonia and bacteremia caused by *Pseudomonas aeruginosa*). After intraperitoneal injection, it was found to be efficient at 10-fold lower therapeutic concentrations than specific antibiotics (methicillin and carbapenem), displaying a favorable safety profile in the reported study. Furthermore, the survival of mice during lethal bacteremia challenge was largely improved, with no death observed, unlike the 80% death rate observed for untreated mice [44].

An analogous dendron, in which the primary amines were transformed into tertiary amines, was also synthesized (**C18-G3-Me**). It was tested and found to be efficient towards a panel of Gram-positive and -negative bacterial strains, including resistant strains of *S. aureus*, *Enterococcus faecium*, *Klebsiella pneumoniae*, *E. coli*, *Acinetobacter baumannii*, and *Enterobacter* species. **C18-G3-Me** was also found to be effective in vivo in mouse models of bacteremia, acute pneumonia, and peritonitis-sepsis, with infection models based on *E. coli* and *A. baumannii* (both resistant strains). At a dose of 5.0 mg·kg^−1^, a significant decrease (1000-fold) of the bacterial burden, compared to tigecycline at the high dose of 50 mg·kg^−1^, was observed. The same efficacy was observed in the case of acute pneumonia. A notably higher survival rate was observed in the case of peritonitis-sepsis (90% survival in the case of *E. coli* and 80% in the case of *A. baumannii*) to be compared to untreated mice (12% survival in the case of *E. coli*, and less than 10% in the case of *A. baumannii*) [45].

**Scheme 3:**
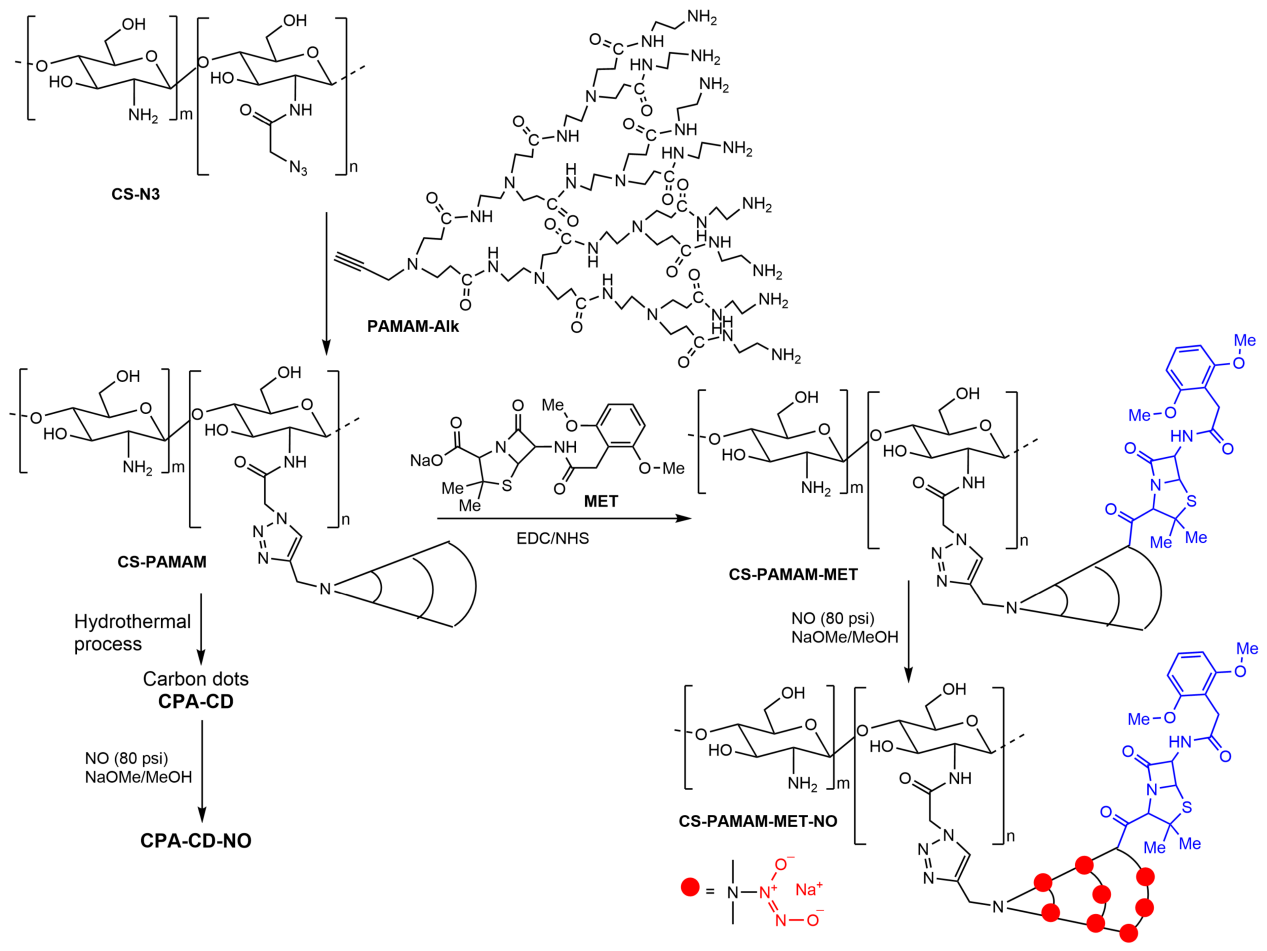
Chitosan functionalized with PAMAM dendrons, and grafting of methicillin and NO.

PAMAM dendrons bearing an alkyne group at the core were also grafted onto azide-functionalized chitosan through click reactions. Some of the dendron amino groups were then reacted with methicillin, while nitric oxide (NO) was conjugated both at the surface and within the dendrons, affording **CS-PAMAM-MET-NO** (Scheme 3). This system combined the antibacterial effect of methicillin and NO and showed activity against *E. coli* and *S. aureus* in vitro. In a rat model of wounds infected by MRSA, **CS-PAMAM-MET-NO** effectively accelerated wound healing in vivo [46].

A hydrothermal carbonization process was applied to **CS-PAMAM** (Scheme 3) dissolved in acetic acid solution and heated at 180 °C for 12 h in a furnace, followed by centrifugation. Only the fluorescent deposits were taken, affording functionalized carbon dots (**CPA-CD**), further functionalized with NO, as shown in Scheme 3, to afford **CPA-CD-NO**. This material was used as an NO-releasing platform against *P. aeruginosa*, decreasing both the bacterial viability and biofilm, as demonstrated by images obtained thanks to the bright blue fluorescence of **CPA-CD-NO**. In vivo theranostic assays were carried out to evaluate the treatment of bacteria-infected rat wounds, with eradication of the severe infection and inflammation [47].

**Scheme 4:**
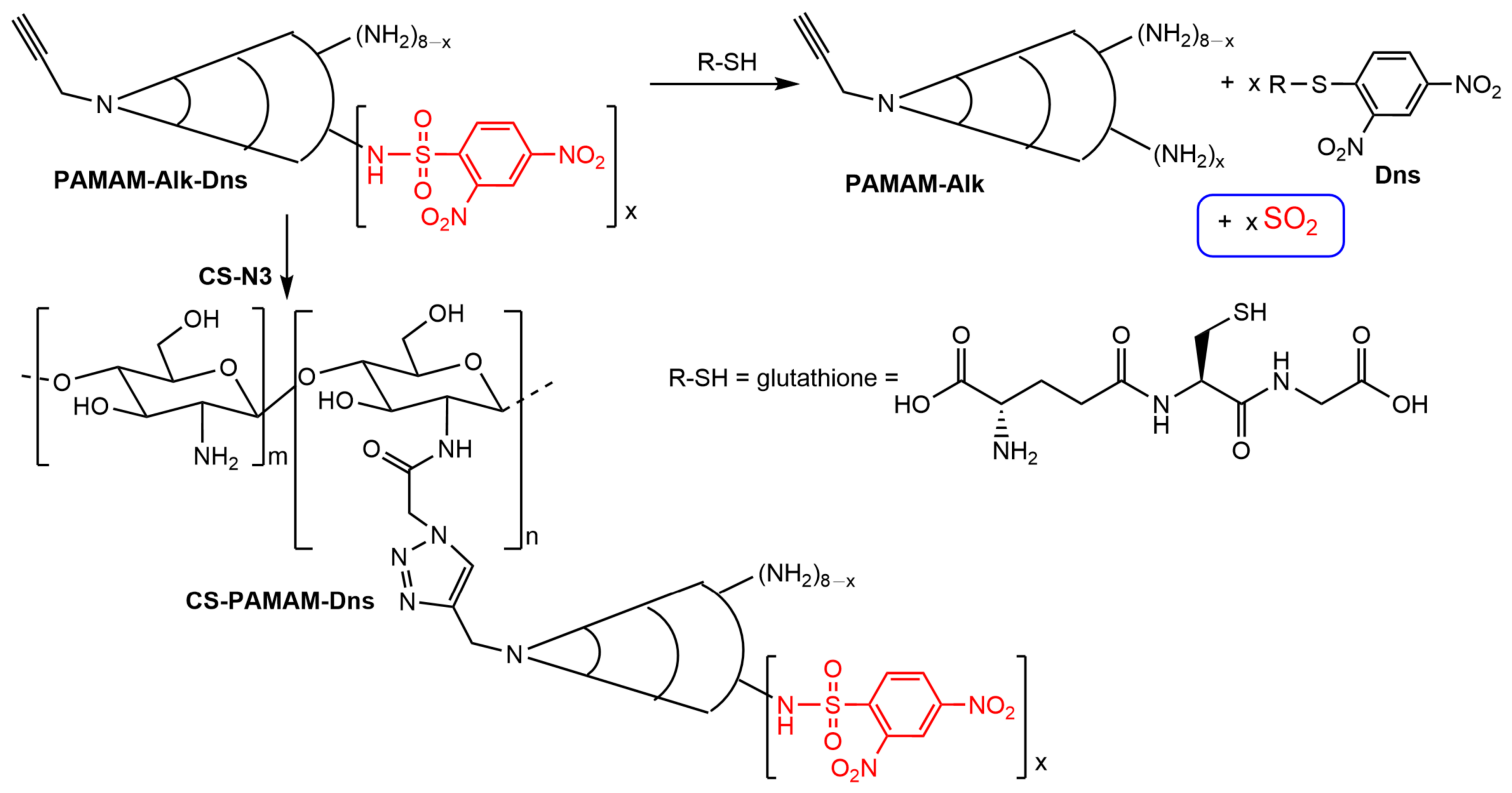
Chitosan functionalized with PAMAM dendrons, suitable for releasing SO_2_.

**PAMAM-Alk** (Scheme 3) was functionalized with 2,4-dinitrobenzenesulfonyl chloride, affording **PAMAM-Alk-Dns**. This compound was found to be suitable for releasing SO_2_ in the presence of glutathione. It was grafted to **CS-N3**, affording **CS-PAMAM-Dns** (Scheme 4). This functionalized polymer, when tested in vitro, displayed antibacterial activity against the Gram-positive bacterium *S. aureus* and its methicillin-resistant strain MRSA, as well as the Gram-negative bacteria *E. coli* and *P. aeruginosa*. The in vivo efficacy of **CS-PAMAM-Dns** was tested in the small, transparent nematode *Caenorhabditis elegans* infected with MRSA. The survival rate of untreated *C. elegans* was only 49.7%, whereas for those treated with **CS-PAMAM-Dns** at concentrations of 1 and 2 mg·mL^−1^, the survival rates were 84.6% and 90.3% at 24 h, respectively [48].

### 3.2. PAMAM Dendrimers and Dendrons as Non-Covalent Carriers of Antimicrobial Drugs

**Figure 6:**
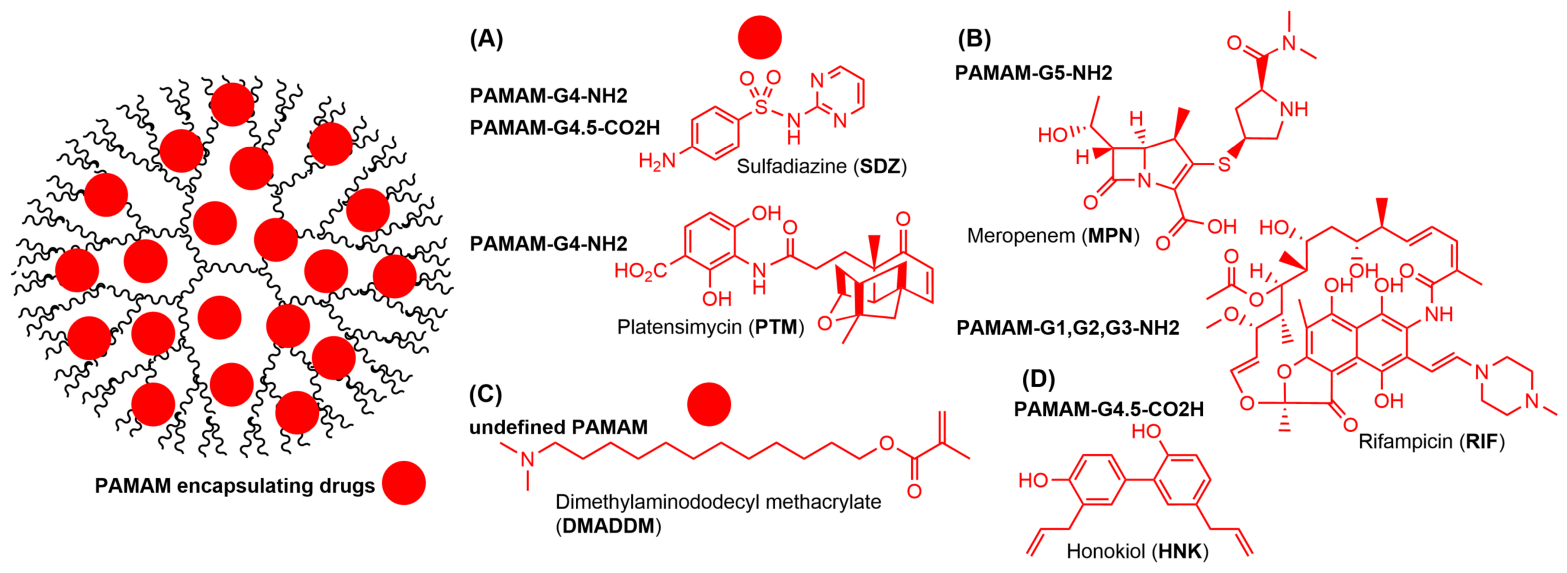
Antibacterial drugs encapsulated inside PAMAM dendrimers. (**A**,**B**) Miscellaneous examples. (**C**,**D**) Dendrimer associations used to treat dental infections.

Examples of antibacterial drugs covalently linked to PAMAM dendrimers have been shown in the previous paragraphs. This new part concerns the non-covalent encapsulation or entrapping of antimicrobial drugs by PAMAM dendrimers, for which examples are shown in Figure 3 and Scheme 1. Generation 4 with 64 NH_2_ terminal groups (**PAMAM-G4-NH2**) and generation 4.5 with 128 carboxylate terminal groups (**PAMAM-G4.5-CO2H**) were used for the encapsulation of sulfadiazine (**SDZ**) (Figure 6A), a drug used for the treatment of toxoplasmosis, an infection caused by the *Toxoplasma gondii* parasite. However, this drug induces severe collateral effects; thus, it was encapsulated inside PAMAM dendrimers in the hope of limiting its unwanted effects and increasing the drug delivery efficiency. No large difference was observed between the free drug and its association with the dendrimers when they were administered orally to rats. On the contrary, after i.v. administration, the plasma concentration of **SDZ** was higher when complexed with PAMAM dendrimers than when injected free, and increased delivery was also observed to muscles, kidneys, and brain. These effects were more significant with **PAMAM-G4-NH2** than with **PAMAM-G4.5-CO2H** [49].

Platensimycin (**PTM**) is a promising drug against Gram-positive bacteria, including many drug-resistant bacteria, but it is hampered by its too-rapid renal clearance. **PTM** was formulated with **PAMAM-G4-NH2** to try to enhance its antibacterial properties (Figure 6A). Treatment by intraperitoneal (i.p.) injection in a mouse peritonitis model of MRSA using **PTM** encapsulated in **PAMAM-G4-NH2** led to full survival of the mice. On the contrary, all infected mice treated with free **PTM** only died, demonstrating the great usefulness of this encapsulation strategy [50].

Rifampicin (**RIF**) is a first-line drug for the treatment of tuberculosis caused by *Mycobacterium tuberculosis*. This pulmonary disease results in severe morbidity and mortality [51]. A review has recently emphasized the role of some dendrimers, tested essentially in vitro, against *M. tuberculosis* [52]. Generations 1, 2, and 3 of PAMAM dendrimers were used for the encapsulation of rifampicin and co-spray-dried to produce inhalable microspheres for pulmonary delivery (Figure 6B). These microspheres were administered to healthy rats via the intratracheal route. The peak plasma concentration time of rifampicin depended on the generation of the dendrimer: 12 h for **PAMAM-G1-NH2** and **PAMAM-G2-NH2** and 24 h for **PAMAM-G3-NH2** [53]. Thus, the work mainly supports the feasibility of PAMAM-based inhalable rifampicin delivery and prolonged pulmonary/systemic exposure but does not demonstrate antimicrobial efficacy in an infected tuberculosis model.

Meropenem (**MPN**) is an antibiotic that is hydrolyzed by New Delhi metallo-β-lactamase (NDM) produced by superbugs during infections, which are particularly difficult to treat. To test whether encapsulation of **MPN** could restore its activity, an **MPN**-loaded **PAMAM** dendrimer was produced (Figure 6B) and then subjected to ultrasonication into platelet membrane nanovesicles (PMVs). Such nanovesicles have pH-responsive properties to precisely deliver both **PAMAM** and **MPN** to bacterial infection sites. **PAMAM-G5-NH2** inhibits NDM activity by removing zinc ions, thus preventing hydrolysis of **MPN** and boosting its efficacy. First, in vivo experiments were carried out with bacterial-enriched foci of zebrafish larvae, in which the loaded PMV specifically accumulated. In mouse models of *E. coli*-induced pneumonia or muscle sepsis, the delivery of loaded PMV by injection into the tail vein resulted in accumulation of **MPN**-loaded **PAMAM** at the infections sites. This method efficiently protected all infected mice from death [54].

Several recent papers concern the treatment of dental infections with PAMAM dendrimers interacting with drugs. A first example was a PAMAM dendrimer (generation and type of end groups unknown) loaded with dimethylaminododecyl methacrylate (**DMADDM**) (Figure 6C), a strong but cytotoxic antibacterial agent. Encapsulation decreased **DMADDM** toxicity, and encapsulation rate was 31%. **PAMAM/DMADDM** was used for treatment with micro-arc oxidation (MAO), which has antibacterial and osteogenic properties. This concept was used for a treatment in a model of severe peri-implantitis. Female rats that were highly infected with the micromycete *Candida albicans* and the Gram-positive bacterium *S. aureus* received an implant in the femur made of titanium coated or not with **PAMAM/DMADDM**, using the MAO process or not. It was shown that this association both decreased the development of microorganisms and promoted osteogenesis and osteointegration [55].

Honokiol (**HNK**) is a plant-derived lignan with antibacterial properties that is able to inhibit the growth of many cariogenic pathogens, but its water-insolubility limits its application. Encapsulation of **HNK** in PAMAM dendrimers functionalized with 128 carboxylates (**PAMAM-G4.5-CO2H**) (Figure 6D) endowed **HNK** with long-term antibacterial activity. It was shown that the release rate of **HNK** depended on the pH. Male rats fed a cariogenic diet developed a bacterial infection. Those that received **PAMAM-G4.5-CO2H/HNK** every 3 days developed significantly fewer pits and fissure lesions than the untreated group and exhibited decreased levels of *S. mutans*, one of the major cariogenic pathogens. This treatment induced enamel remineralization and effectively inhibited caries lesions in rats [56].

**Figure 7:**
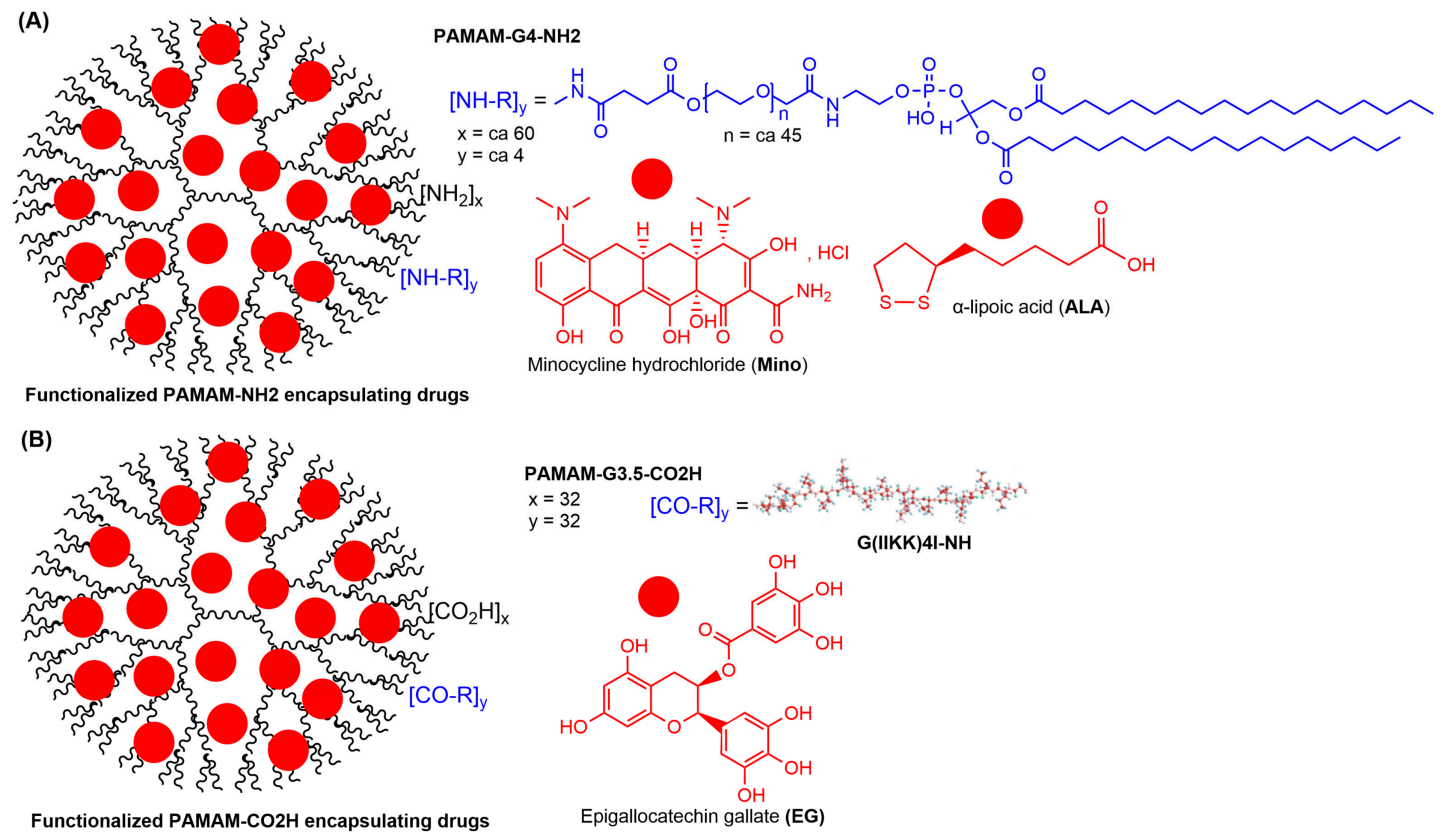
(**A**) **PAMAM-NH_2_** dendrimers covalently functionalized and encapsulating antibiotics. (**B**) **PAMAM-CO_2_H** dendrimers covalently functionalized and encapsulating antibiotics.

Another example concerned loading of the antibiotic minocycline hydrochloride (**Mino**), commonly used in clinical periodontal treatment, and the antioxidant α-lipoic acid (**ALA**) into **PAMAM-G4-NH2** functionalized with hydrophobic lipid formed by 1,2-distearoyl-sn-glycero-3-phosphoethanolamine-poly(ethylene glycol) (Figure 7A). In this system, **ALA** could be delivered under lipase stimulation, whereas **Mino** could be delivered at low pH. This system was tested in diabetic rats to evaluate its antibacterial efficacy and its periodontal regeneration ability. Periodontitis (periodontal bone loss) is a complication of diabetes. The system was found to disrupt dental plaque biofilms and suppress periodontal bone loss in diabetic rats [57].

Epigallocatechin gallate (**EG**), a bioactive polyphenol, inhibits matrix metalloproteinases in saliva through zinc chelation and disintegrates biofilm architecture. **EG** was encapsulated within **PAMAM-G3.5** having at the periphery 32 synthetic antimicrobial peptides (G(IIKK)4I–NH2, **AMP**), attached covalently by amide bonds and 32 CO_2_H for Ca^2+^ and PO_4_^3−^ sequestration (Figure 7B). This assembly displayed both biofilm disintegration and collagen protection for comprehensive prevention and treatment of dentin caries. Antimicrobial assays demonstrated a bactericidal efficacy of 99.75% in vitro. In vivo experiments with a rat caries model demonstrated both decreased salivary *S. mutans* counts and increased molar mineral density for the rats treated with **PAMAM-G3.5-AMP/EG** compared to untreated rats [58].

**Figure 8:**
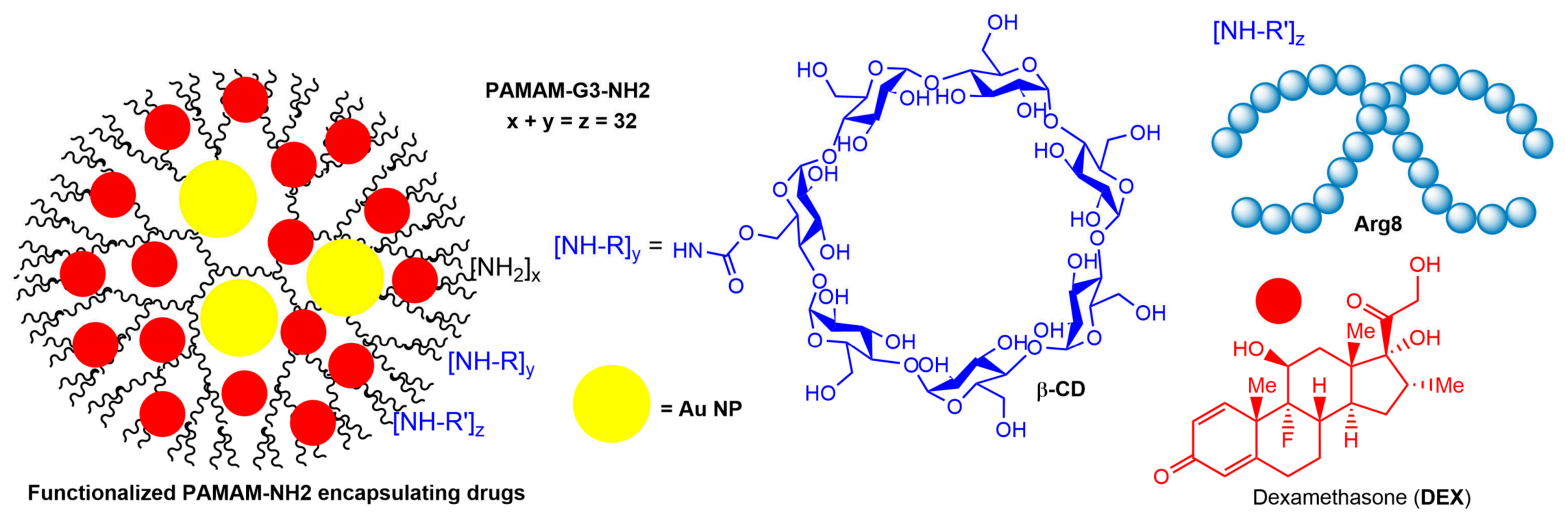
PAMAM covalently functionalized with β-cyclodextrin (CD) and the oligoarginine peptide Arg8 and encapsulating Au nanoparticles and dexamethasone.

**PAMAM-G3-CO2H** was partly functionalized with **CD** at the periphery, then used to entrap gold nanoparticles (AuNPs), before being further covalently functionalized with the oligoarginine peptide (Arg8), and finally used to encapsulate either dexamethasone (**DEX**) or dexamethasone fluoresceine (**DEX-FITC**) (Figure 8). **CD** was chosen to enhance the biocompatibility, AuNPs to measure the distribution of the dendrimer in important organs using ICP-AES (inductively coupled plasma atomic emission spectroscopy), and Arg8 to improve cellular internalization. Male mice with streptomycin (SM)-induced ototoxicity were injected with either **DEX** alone or **DEX** encapsulated in **PAMAM-G3-CD-Arg8/AuNP/DEX**. It was shown that improvement of metabolism in the cochlea was greatest with the intratympanic injection route, followed by the intravenous, intramuscular, and retro-auricular sulcus injection routes. In all cases, **PAMAM-G3-CD-Arg8/AuNP/DEX** was more efficient than **DEX** alone [59]. Thus, the study mainly illustrates the potential of PAMAM-based delivery to improve local dexamethasone exposure in a model of anti-infective drug-associated ototoxicity, rather than direct antimicrobial efficacy.

**Scheme 5:**
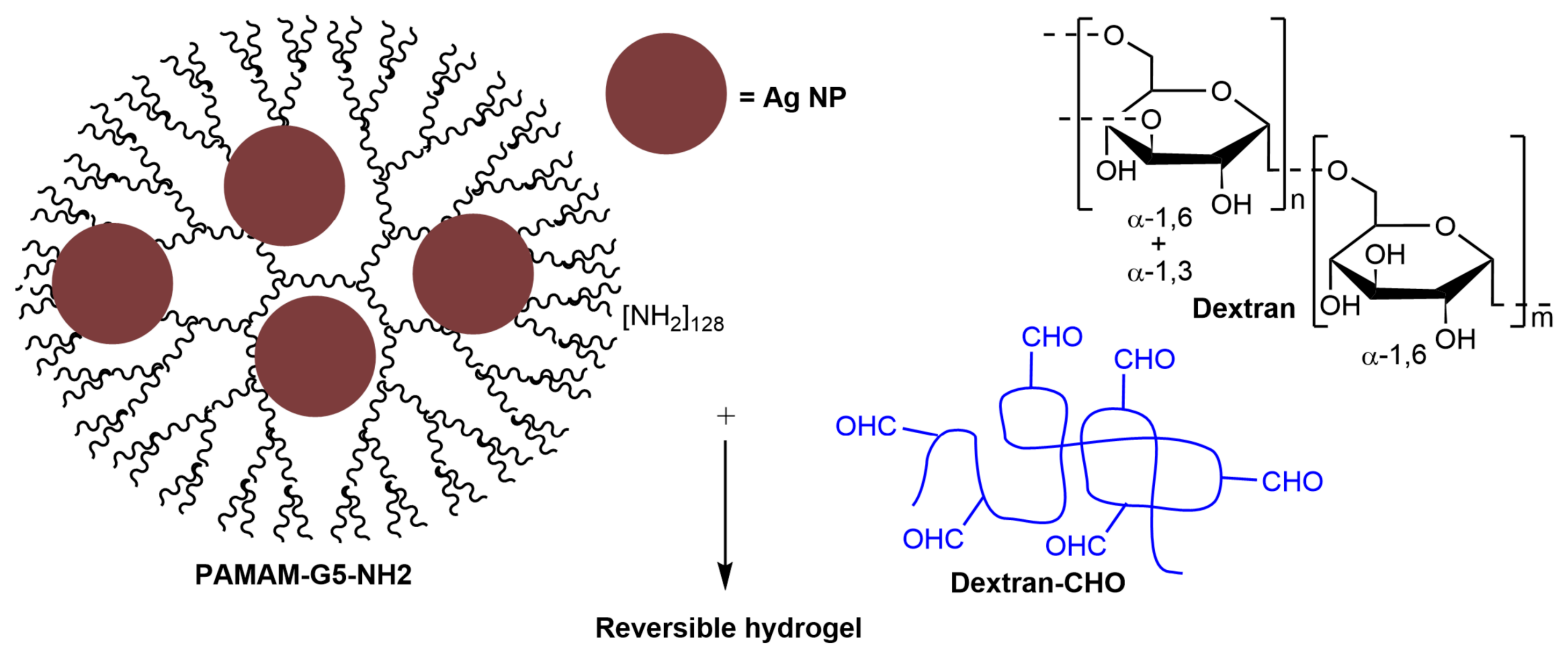
Hydrogel formed by condensation reactions between PAMAM and oxidized dextran.

A pH-responsive hydrogel was obtained by condensation of **PAMAM-G5-NH2** encapsulating silver nanoparticles, with oxidized dextran functionalized with aldehydes (Scheme 5). The Schiff bases (C=N linkages) can be reversibly cleaved in acidic media, in particular generated by growing bacteria. The released silver nanoparticles and the cationic PAMAM dendrimers exhibited a synergistic effect, displaying strong antibacterial activity against both Gram-negative (*E. coli* and *P. aeruginosa*) and Gram-positive bacteria (*Staphylococcus epidermidis* and *S. aureus*). Mice were treated (or not) with this hydrogel, then were infected with *S. aureus* via direct injection at the hydrogel sites. The treated mice exhibited a much lower bacterial burden in the skin lesion than those untreated or treated with a commercial Ag gel [60].

**Scheme 6:**
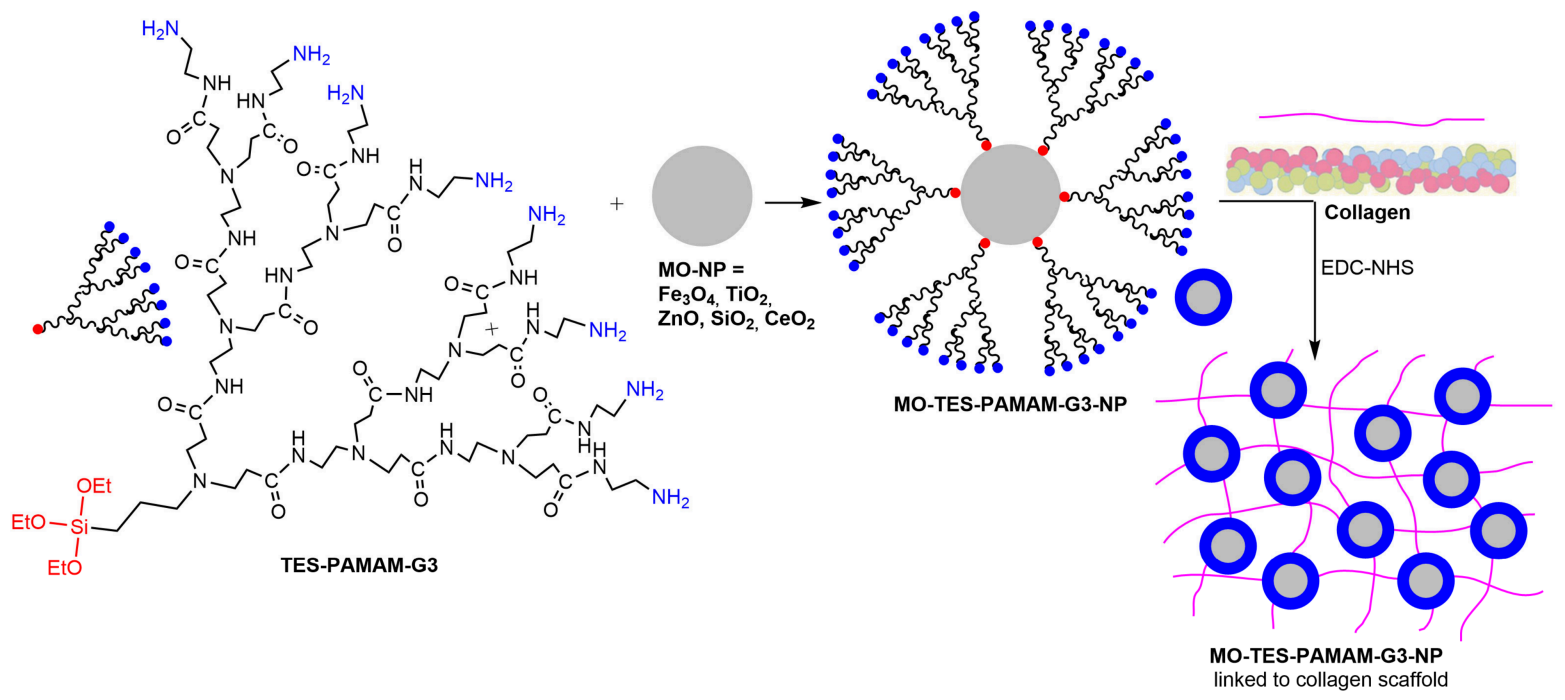
PAMAM dendrons used for coating metal oxide NPs that were then cross-linked with collagen.

A PAMAM dendron with a triethoxysilyl (**TES**) group at the core and eight NH_2_ functions on the surface (**TES-PAMAM-G3**) was used to coat different types of metal oxide nanoparticles (**MO-NP**) with sizes from 12 to 25 nm (Fe_3_O_4_, TiO_2_, ZnO, SiO_2_, CeO_2_). These coated NPs were then covalently cross-linked with triple-helical collagen through EDC-NHS (1-Ethyl-3-(3-dimethylaminopropyl)carbodiimide and *N*-hydroxysuccinimide) coupling with the NH_2_ functions (Scheme 6). These assemblies were used for skin regeneration of wounds in Wistar albino rats. The best antibacterial activity was obtained with the **ZnO-TES-PAMAM-G3-NP-collagen** scaffold, which accelerated skin regeneration compared to all the other cases studied in the paper [61].

**Figure 9:**
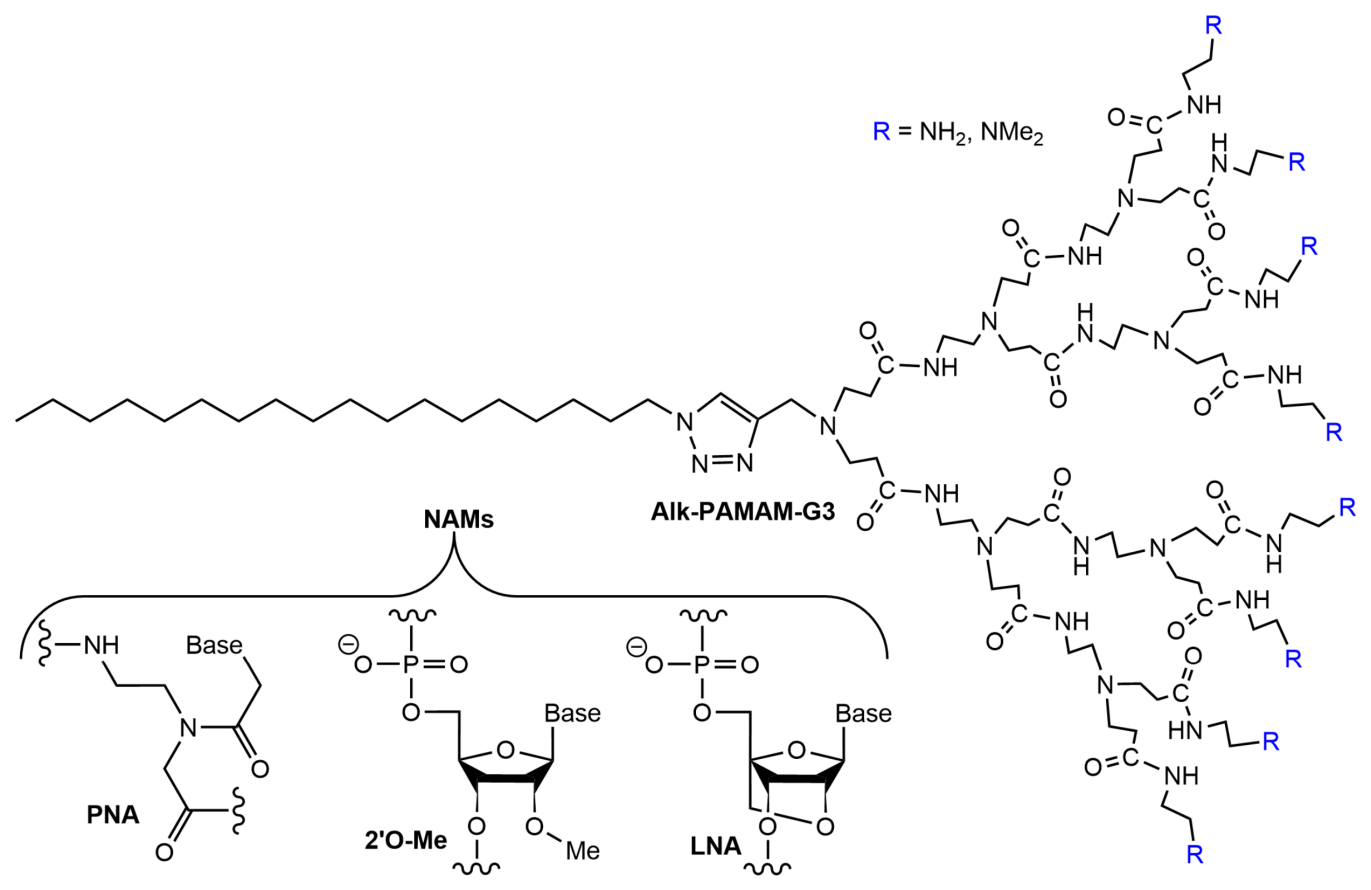
PAMAM dendron and oligonucleotides based on nucleic acid mimics (**NAMs**).

Another PAMAM dendron with a long alkyl chain at the core, linked through copper-catalyzed azide-alkyne cycloaddition (CuAAC) click chemistry and functionalized with eight primary amines (NH_2_) or tertiary amines (NMe_2_), self-assembled in water to form nanomicelles. These nanomicelles were used as carriers of oligonucleotides based on nucleic acid mimics (**NAMs**), in particular for the delivery of antisense **NAMs** in *E. coli*, selected for their ability to target the essential *acpP* gene in *E. coli*. These **NAMs** were peptide nucleic acid (**PNA**)-modified ribose entities, such as 2′-O-methyl RNA (**2′O-Me**) or locked nucleic acid (**LNA**) (Figure 9). The most efficient formulation was the one based on the tertiary amine dendron associated with **PNA**. This association was tested in a greater wax moth (*Galleria mellonella*) larva model. No significant toxicity was found regarding either survival or the overall health of the larvae [62].

### 3.3. Overall Assessment of PAMAM Dendrimers

Overall, PAMAM dendrimers and dendrons represent one of the most extensively explored dendritic platforms for antimicrobial applications in vivo. Their main strengths are their structural versatility, the availability of multiple terminal functions, and their ability to act either as antimicrobial agents themselves or as carriers for antibiotics, antibiofilm agents, nitric oxide, or other therapeutic payloads. The in vivo studies summarized above highlight their potential for the treatment of local infections, biofilm-associated infections, and pulmonary infections, but also for wound healing, dental applications, and selected systemic models. However, the biological outcome strongly depends on the dendrimer generation, surface charge, terminal groups, route of administration, and formulation. In particular, cationic PAMAM dendrimers may display potent antimicrobial effects but also raise toxicity concerns, whereas neutral or anionic derivatives generally show improved tolerability but may require additional functionalization to achieve strong antimicrobial activity. Thus, PAMAM-based systems appear to be highly adaptable, but their further development will require careful optimization of safety, biodistribution, degradability, physicochemical characterization, and reproducibility.

## 4. Peptide Dendrimers

Even though numerous types of peptide dendrimers were synthesized, the most studied derivatives were those based on L-Lysine.

### 4.1. Poly(L-Lysine) Dendrimers

Poly(L-Lysine) (**P-Lys**) dendrimers were the first large dendrimers patented [63] and are the most widely used type of dendrimers in clinical trials [64], essentially carried out by/for the Starpharma company. A review has emphasized the early instances of their use for HIV (human immunodeficiency virus) and STI (sexually transmitted infection) prevention [65].

**Figure 10:**
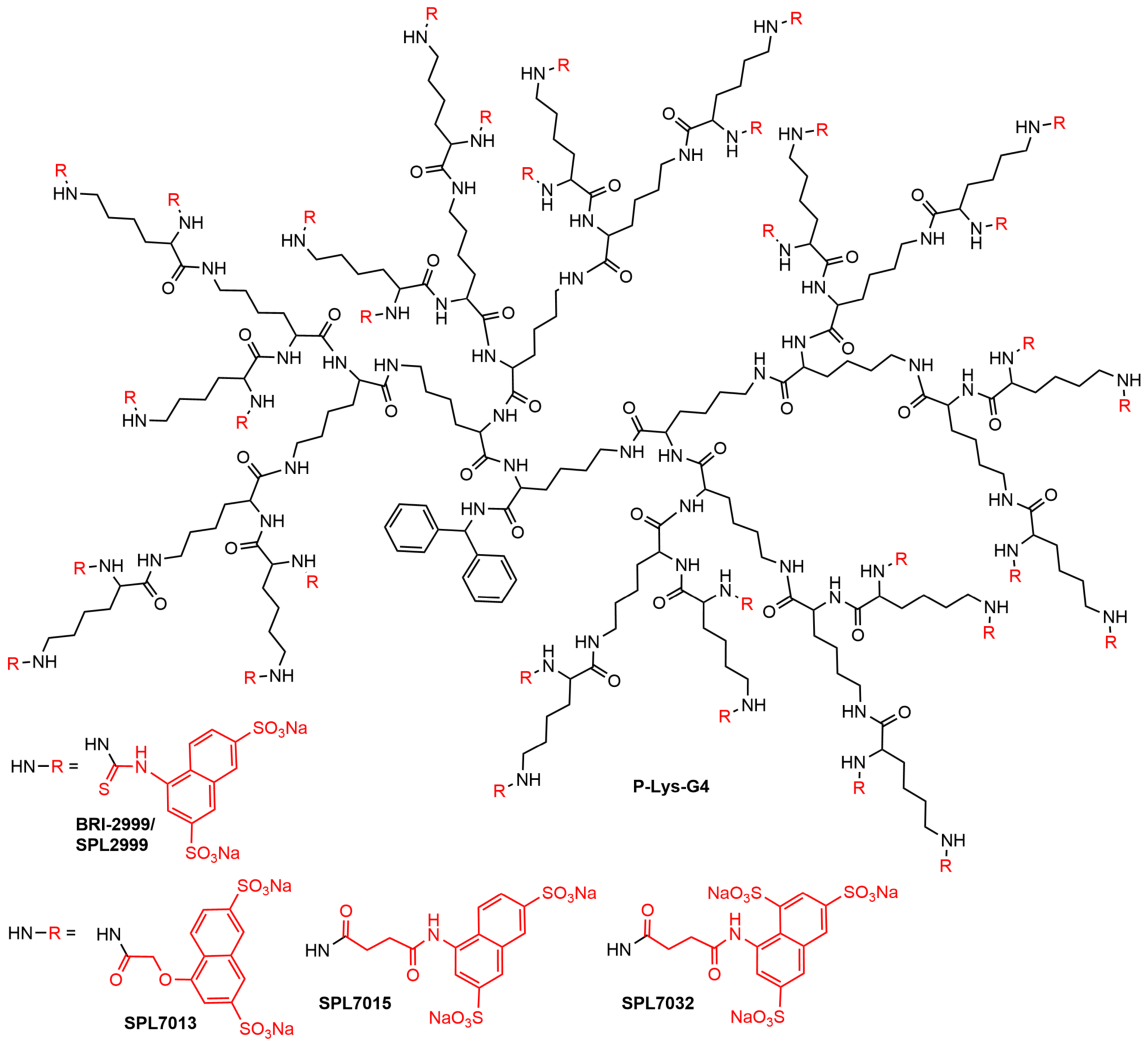
Poly(L-Lysine) dendrimer functionalized with aromatic-sulfonic acids on the surface through different types of linkers. **SPL7013** is the lead compound, used in many clinical trials.

The fourth-generation **P-Lys** dendrimer functionalized with naphthyl disodium disulfonate (**BRI-2999/SPL299** in Figure 10) and the fifth generation functionalized with phenyl disodium dicarboxylate were evaluated as topical microbicides in a mouse model of genital HSV-2 (herpes simplex virus) infection. It was shown that **BRI-2999** was the most active, but it should be used before and not after contact with the virus [66]. Three new microbicidal candidates from the same family were further identified, named **SPL7013**, **SPL7015**, and **SPL7032** (Figure 10). These compounds were tested either unformulated or included in three different formulations of gels in mouse and guinea pig models of HSV-2. Similar results were obtained with the four dendrimers, but, due to easier manufacturing, lower cost, and better stability, **SPL7013** was selected for further development. The concentrations of the active ingredient (**SPL7013**) in the gel were between 1 and 5%. Evaluation in a mouse model of genital HSV infection permitted the most active formulation to be chosen. This formula, based on Carbopol^®^ gel, was then tested in a guinea pig model of genital herpes, a better mimic of human disease. Concentrations of 3% or higher of the formulated product were found to be necessary for optimal protection [67]. **SPL7013** was then tested in cell cultures infected with HIV strains and showed high antiretroviral efficacy; thus, it was tested in a gel as topical microbicide for the prevention of vaginal transmission of SHIV89.6P in 44 adult female pigtailed macaques. Dose-dependent protection from HIV infection was observed, with all six macaques (6/6) treated with 5% *w*/*w* **SPL7013** gel protected from vaginal transmission of HIV, unlike only two of six macaques treated with 1% *w*/*w* **SPL7013** gel. Importantly, all **SPL7013**-protected macaques remained virus-negative throughout the 36-week follow-up period [68]. The next step consisted of repeated vaginal and rectal applications in pigtailed macaque, which were shown to be nonirritating [69]. Dendrimer **SPL7013** and the corresponding second generation were tested against HIV-1 and HSV-2. **SPL7013** was the most efficient and provided at least 12 h of protection against HSV-2 in the mouse vagina [70].

The first clinical trial (Phase 1) with this **P-Lys** dendrimer **SPL7013** concerned safety for men. They were asked to apply the gel to their penis and to abstain from all sexual activity for 7 days. Great interest in the concept of microbicides was the main conclusion of the study [71,72]. A randomized trial was carried out with 3% **SPL7013** gel or placebo gel. No safety or tolerability issues with **SPL7013** gel were observed (36 healthy men were involved) [73]. Clinical trials with healthy women focused first on safety, with randomized vaginal application of **SPL7013** gel (concentrations of 0.5% to 3%) or placebo gel once daily for 7 consecutive days. No evidence of systemic toxicity or absorption was observed [74]. This gel was then named VivaGel^®^ and was used in a clinical Phase 1 dose-ranging study involving 37 women for 7 days. It was also tested in 54 young women (placebo-controlled) who used it vaginally twice daily for 14 days. Mild epithelial irritation and inflammation occurred more commonly among women using VivaGel^®^ [75]. Cervical specimens were collected for analysis of cytokines, chemokines, T cells, and dendritic cells. Several mucosal immune parameters were increased in the VivaGel^®^-treated group compared with the placebo group [76]. The samples were also used to test inhibition of *E. coli*, but no clear trends could be inferred [77]. A second Phase 1 trial involved 61 young women (placebo controlled) using this gel vaginally twice daily for 14 days. This trial led to the same conclusion [78,79]. In another trial, young women received five single doses of the product, with at least 5 days between doses, and cervicovaginal fluid samples were collected to test the antiviral activity against HIV-1 and HSV-2 in vitro. For samples taken 3 h after application of VivaGel^®^, the inhibition of HIV-1 was 96%, and that of HSV-2 was 94% [80].

A Phase 2 placebo-controlled clinical trial was initiated to determine the dose ranging efficacy and safety. A new name for the gel containing the **SPL7013** dendrimer was proposed: astodrimer. The doses of the dendrimer were 0.5%, 1%, or 3%, and the gel was applied vaginally once daily for 7 days by 132 women with bacterial vaginosis. Treatment with the dendrimer was superior to the placebo and was well tolerated. The strongest efficacy for the treatment of bacterial vaginosis was obtained with the 1% dose [81]. Two Phase 3 clinical trials were carried out in parallel for women with bacterial vaginosis, one in the United States for astodrimer 1% at a dose of 5 g vaginally once daily for 7 days (N = 127) or placebo (N = 123), and the second one in the United States, Germany, and Belgium using the same treatment conditions (N = 128) or placebo (N = 123). The primary endpoint was defined as absence of bacterial vaginosis vaginal discharge at Day 9–12, whereas the secondary endpoint included clinical cure at Day 21–30. The conclusion was that this treatment is an effective, safe, and well-tolerated treatment for women with bacterial vaginosis [82].

Another Phase 3 clinical trial concerned the prevention of bacterial vaginosis recurrence. It involved 864 women with a diagnosis of bacterial vaginosis and a history of recurrent bacterial vaginosis who first received oral treatment with metronidazole. Women successfully treated with metronidazole then used astodrimer (N = 295) or placebo (N = 291) vaginally every second day for 16 weeks (56 doses total). At the end of the treatment with astodrimer, bacterial vaginosis recurred in 44.2%, whereas the rate of recurrence was 54.3% for those receiving placebo. Recurrence of subject-reported vaginosis up to 8 weeks after cessation of therapy was significantly lower in the astodrimer group than in the placebo group (36.1% versus 45.5%, respectively) [83]. A review has gathered all these clinical trials for the treatment of bacterial vaginosis with the astodrimer gel [84]. Overall, these clinical studies support the efficacy and tolerability of astodrimer 1% gel for the treatment and prevention of recurrent bacterial vaginosis. Astodrimer-containing products have subsequently been marketed in several regions, particularly in Europe, Asia, Australia, and New Zealand, under different product names depending on the market.

The antiviral activity of the same astodrimer gel was also tested in other diseases, in particular ocular adenovirus infection and SARS-CoV-2. In the first case, the ocular tolerability and anti-adenoviral efficacy were tested in a rabbit model. It was shown that this gel was non-toxic to rabbit eyes and had anti-adenoviral activity, albeit slightly lower than that of the reference treatment cidofovir [85]. The astodrimer gel was reformulated in a mucoadhesive nasal spray and used against SARS-CoV-2. Mice received astodrimer sodium 1% nasal spray or PBS intranasally, or intranasally and intratracheally, for 7 days. They were infected intranasally with SARS-CoV-2 at Day 0, just after the first treatment. Astodrimer significantly reduced the number of viral genome copies (>99.9%) and the levels of infectious virus (~95%) in the lung and trachea of mice versus PBS (phosphate-buffered saline). Thus, astodrimer blocked or reduced SARS-CoV-2 replication and its sequelae in a mouse model [86]. In view of this interesting result, a Phase 1 clinical trial was designed to assess the safety, tolerability, and absorption of astodrimer sodium 1% antiviral nasal spray, carried out with 40 healthy volunteers. Thirty received astodrimer, and ten a placebo. They self-administered the spray four times daily for 14 days. No systemic absorption of astodrimer sodium via the nasal mucosa was detected, and it was well tolerated [87]. The efficacy was demonstrated in a Phase 2 clinical trial involving non-hospitalized adults with SARS-CoV-2 infection who used a nasal spray of astodrimer (N = 109) or placebo (N = 113) four times daily from Day 1 to Day 7. The reduction in SARS-CoV-2 load was statistically significant for all age groups, and the effect increased in older age groups (65 years and older) [88]. To provide a more synthetic overview of the largely chronological clinical development of SPL7013/astodrimer, the main preclinical and clinical studies are summarized in Table 1.

The clinical progression of astodrimer also provides important lessons for dendrimer translation. This progression is probably not explained only by intrinsic antiviral or antibacterial potency, but also by the suitability of the product concept for clinical development. Astodrimer is administered locally, mainly as a vaginal gel or nasal spray, thereby limiting systemic exposure and reducing the burden associated with systemic biodistribution and long-term organ accumulation. Its polyanionic surface also differs from many strongly cationic antimicrobial dendrimers, which are often more potent against bacterial membranes but raise greater cytotoxicity concerns. In addition, bacterial vaginosis and topical antiviral indications provide accessible clinical endpoints and allow local formulations with a comparatively simpler development path than systemic anti-infective nanomedicines. Thus, astodrimer illustrates that successful translation of dendrimers depends not only on antimicrobial activity, but also on route of administration, formulation simplicity, safety margin, indication selection, and regulatory feasibility.

**Scheme 7:**
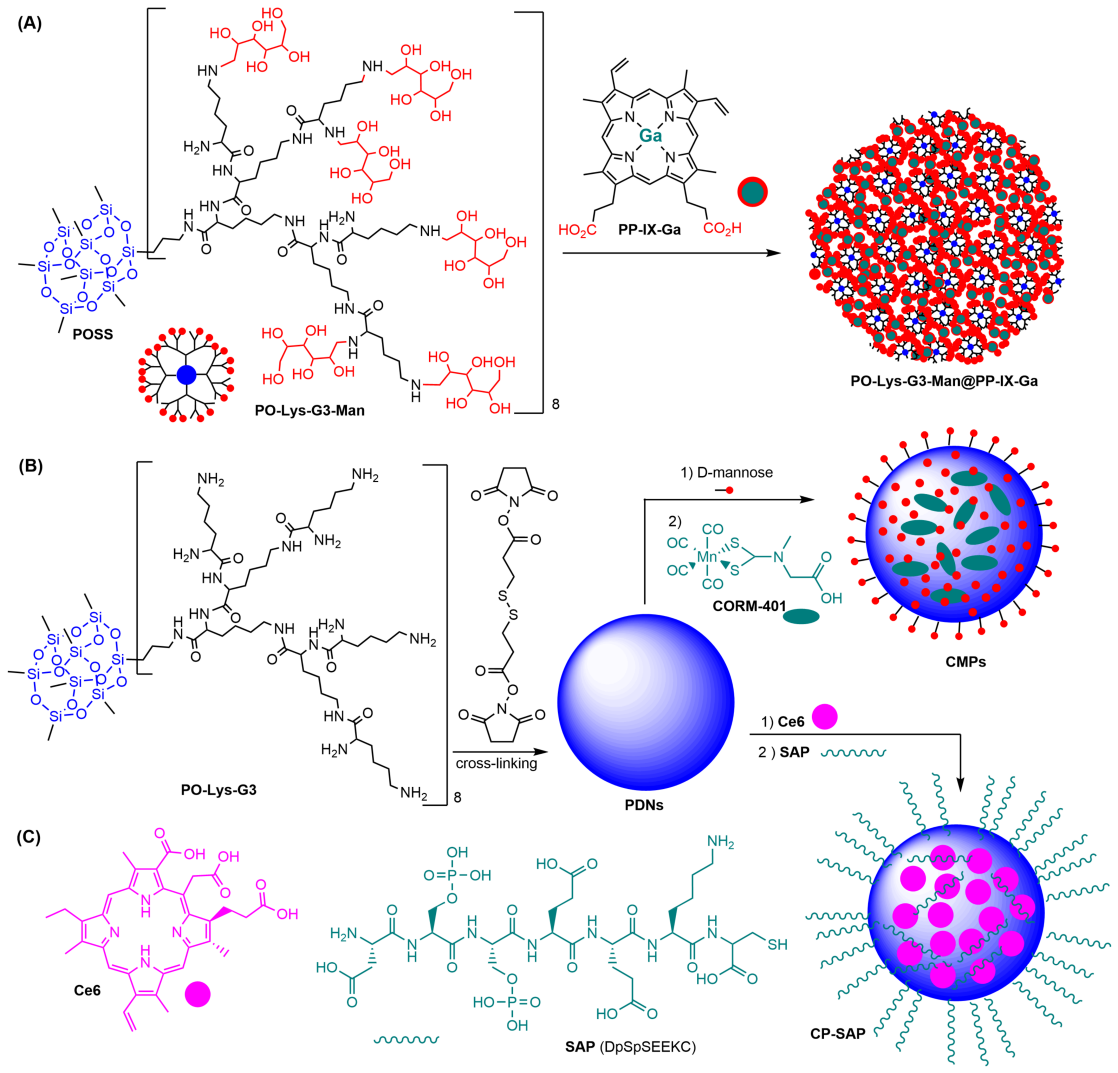
(**A**) Design of **PO-Lys-G3-Man@PP-IX-Ga** nanoparticles. (**B**) Reversible cross-linking of **PO-Lys-G3** and encapsulation of **CORM-401** to form nanogel **CMPs**. (**C**) Encapsulation of **Ce6** in **PDNs**, followed by conjugation with **DpSpSEEKC** to form nanogel **CP-SAP**.

A recent example of P-Lys dendrimer was synthesized from a polyhedral oligomeric silsesquioxane (**POSS**) core up to generation three, with 64 peripheral amine groups. This dendrimer was then randomly functionalized with mannose at 80.69%, affording **PO-Lys-G3-Man** (Scheme 7A). Mixing this dendrimer with **PP-IX-Ga** (protoporphyrin IX complexing gallium) resulted in self-assembly to afford nanoparticles of **PO-Lys-G3-Man@PP-IX-Ga** (mean diameter 282.7 nm, as evidenced by dynamic light scattering (DLS)), with gallium uniformly distributed, as shown by transmission electron microscopy (TEM). The presence of mannose enables targeted delivery to macrophages via mannose receptor recognition, whereas the positive charges (protonated amines) facilitate endosomal escape and release of **PP-IX-Ga** in the acidic intracellular environment. In this environment, free iron ions compete with gallium ions to form the iron−protoporphyrin IX complex (**PP-IX-Fe**), inducing iron metabolism-dependent bacterial death. Furthermore, laser activation of **PP-IX-Ga** generates reactive oxygen species (ROS), further amplifying bacterial killing. This process was tested at a dose of 50 mg·kg^−1^ in mice infected with *S. aureus* (intracellular form). All the treated mice survived to Day 10, whereas all the untreated mice died on Day 5 or before [89].

The **PO-Lys-G3** dendrimer was used in another experiment. It was first cross-linked via reversible disulfide bonds to form poly(L-Lysine) dendrimer-based nanogels (**PDNs**). The surface of the **PDNs** was decorated with mannose, and they were internally loaded with **CORM-401** (a CO-releasing molecule), to afford the **CORM**-mannose dendritic polymers (**CMPs**, Scheme 7B). **CORM-401**, even when encapsulated, can release numerous CO molecules in response to high reactive oxygen species (ROS) levels. **CMPs** were found to be efficient against *S. aureus*, *Citrobacter rodentium*, and *E. coli*. The therapeutic efficacy of **CMPs** was assessed in mice with dextran sulfate sodium (DSS)-induced colitis infected with *S. aureus*, *C. rodentium*, and *E. coli*. Mice received rectal administration of 150 μL of **CMPs**-1.5 (1.5 mg·mL^−1^) or **CMPs**-3 (3 mg·mL^−1^) on Days 1, 3, 5, 7, and 9. **CMPs** scavenged ROS, suppressed inflammatory responses, eliminated pathogens, and alleviated colitis in the mouse model. Furthermore, **CMPs** could remodel gut flora composition by restraining detrimental bacterial communities and augmenting beneficial bacterial abundance [90].

A recent use of **PDNs** consisted of loading it with the photosensitizer **Ce6** then functionalizing it with saliva-acquired peptide (**SAP**, sequence DpSpSEEKC) to confer wet adhesion properties to the resulting **CP-SAP** (Scheme 7C). This functionalized nanogel was tested as a photodynamic antimicrobial and an in situ remineralization agent for dental caries prevention. It was first tested against *S. mutans*, *Streptococcus sanguis,* and *C. albicans*, then in a rat caries model against *S. mutans*. Caries development was induced in rat teeth, after which the nanogel **CP-SAP** was deposited and irradiated with a 665 nm laser. The degree of caries in the **CP-SAP** group was significantly lower than that in the other treatment groups, and higher enamel density was also observed after 21 days of treatment [91].

**Figure 11:**
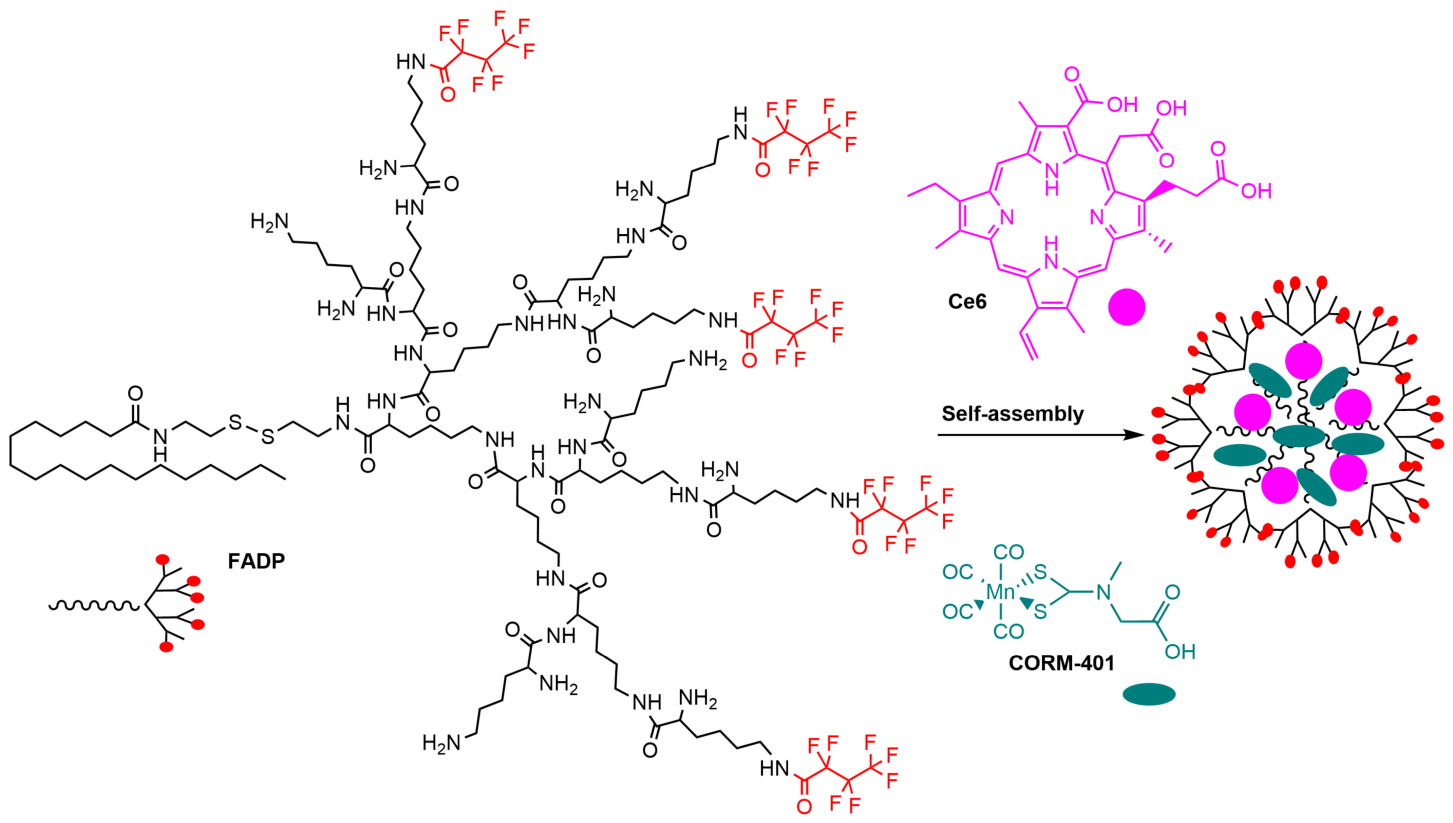
Self-assembled partly fluorinated amphiphilic Lysine dendron (FADP), for encapsulating **Ce6** and **CORM-401**.

A partly fluorinated amphiphilic dendritic peptide based on Lysine (**FADP**) was able to self-assemble in water and was loaded with **Ce6** (chlorin e6, Phytochlorin, a photosensitizer) and **CORM-401** (Figure 11). This assembly (**Ce6&CORM@FADP**) was used in photodynamic therapy (PDT) to generate both ^1^O_2_ from **Ce6** and CO from **CORM-401** using near-infrared (NIR) light irradiation. The association of ^1^O_2_ with CO displayed synergistic antibacterial and biofilm ablation effects against *S. aureus* and *E. coli*, with a survival rate of 0.5% after only 8 min of irradiation. This assembly effectively inhibited biofilm formation and was also capable of efficiently eradicating existing biofilms. It was then used in vivo in mice with *E. coli*-infected subcutaneous wounds. Almost no *E. coli* survived after treatment with **Ce6&CORM@FADP** and two rounds of NIR light irradiation, and the inflammatory response was strongly suppressed. In another experiment, a catheter was pre-colonized with *S. aureus* biofilms and implanted subcutaneously into the inner thighs of mice. After treatment with **Ce6&CORM@FADP** plus NIR light irradiation, all the wounds healed well, without signs of swelling or purulence. Almost no biofilm remained on the surface of the catheters, demonstrating the efficacy of **Ce6&CORM@FADP** in eradicating existing biofilms in vivo [92].

**Figure 12:**
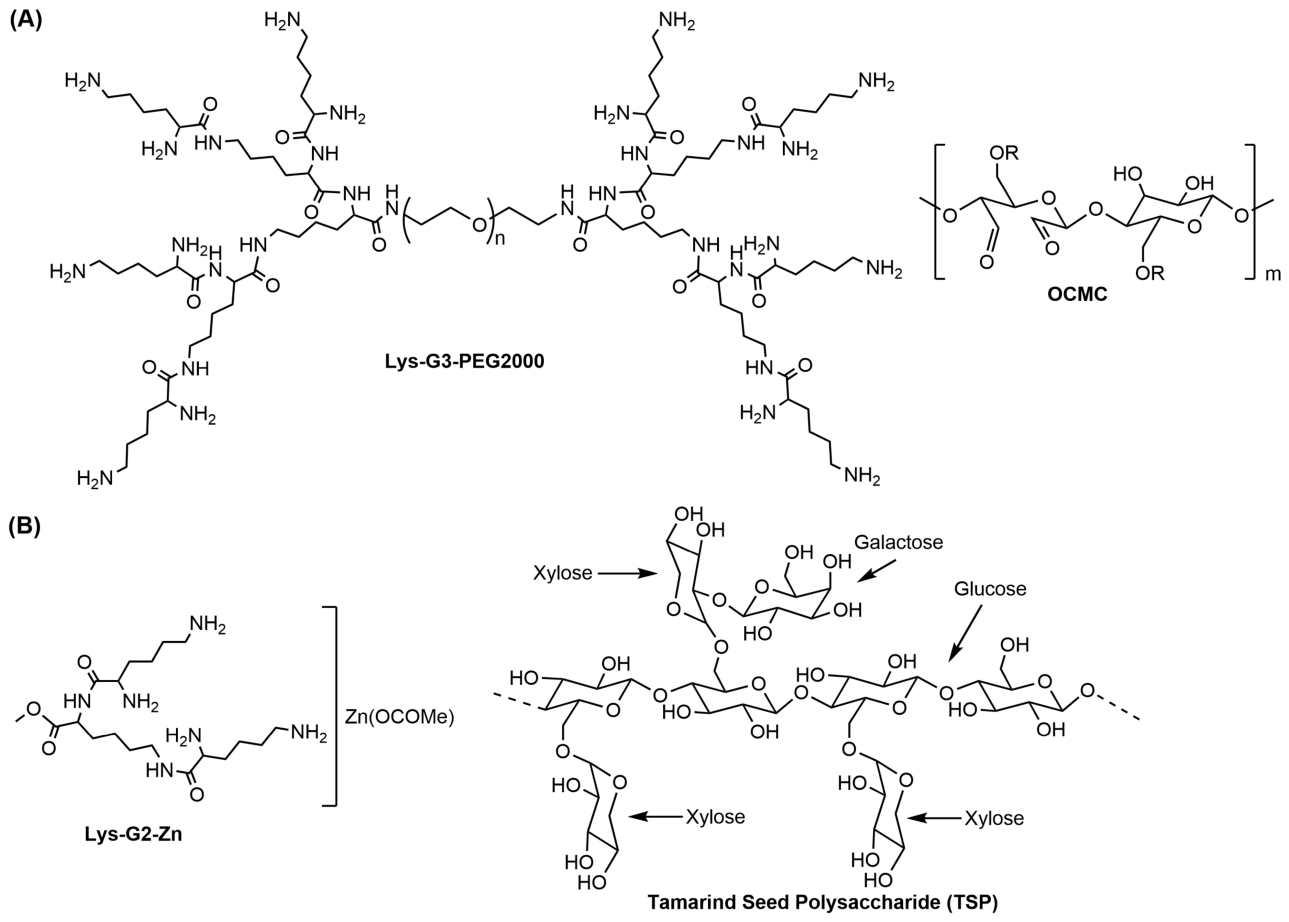
(**A**) Pegylated Lysine dendrimer (**Lys-G3-PEG2000**) and oxidized carboxymethyl cellulose (**OCMC**). (**B**) Lysine dendrimer complexing Zn and tamarind seed polysaccharide (**TSP**).

A barbell-like peptide dendrimer based on generation three Lysine dendrons and PEG2000 at the core (**Lys-G3-PEG2000**) was associated with an oxidized carboxymethyl cellulose (**OCMC**) to generate a hydrogel (Figure 12A). **OCMC** forms imine bonds with endogenous amines of the extracellular matrix (ECM) via a Schiff base reaction, affording adhesive properties and innate hemostatic capability. The amines of **Lys-G3-PEG2000** were also able to react with the aldehydes of **OCMC**, strengthening the adhesive properties. Furthermore, the remaining unreacted amines displayed antimicrobial activities. This hydrogel was tested in vivo on a female rat liver hemorrhage model. The hydrogel was applied to the surface of the trauma site immediately after the injury (a bleeding puncture created with an 18G needle (1.2 mm)), and the hemostatic performance was assessed. The bleeding was stopped in 30 s and was efficiently reduced compared with the control groups. Another in vivo assay involved lacerations on the dorsum of rats. The skin defects treated with the hydrogel exhibited complete wound closure after 2 weeks, with no scars observed. Furthermore, good anti-infective activity of the hydrogel was observed compared to the untreated group [93].

A small Lysine dendrimer was used for complexing Zn, and the complex was then formulated with tamarind seed polysaccharide (**TSP**) (Figure 12B). This association exhibited antibacterial activity against *Propionibacterium acnes*, *S. epidermidis*, *S. aureus*, *E. coli*, and *P. aeruginosa* in vitro. In vivo wound healing studies were carried out by creating, on the dorsal thoracic region of rats, an open excision circular wound with a diameter of 1.6 cm. In comparison with a marketed product (Erytop^®^), the hydrogel of the metallo-dendrimer displayed significant performance with respect to percent re-epithelization and wound contracture [94].

### 4.2. Other Types of Peptide Dendritic Structures

In addition to Lysine dendrimers, peptide dendrimers display highly diverse structures, depending on both the type of peptides and the degree of branching. Several reviews have gathered the antimicrobial properties of dendrimeric peptides [95], and more precisely of antibacterial dendrimeric peptides [96].

**Figure 13:**
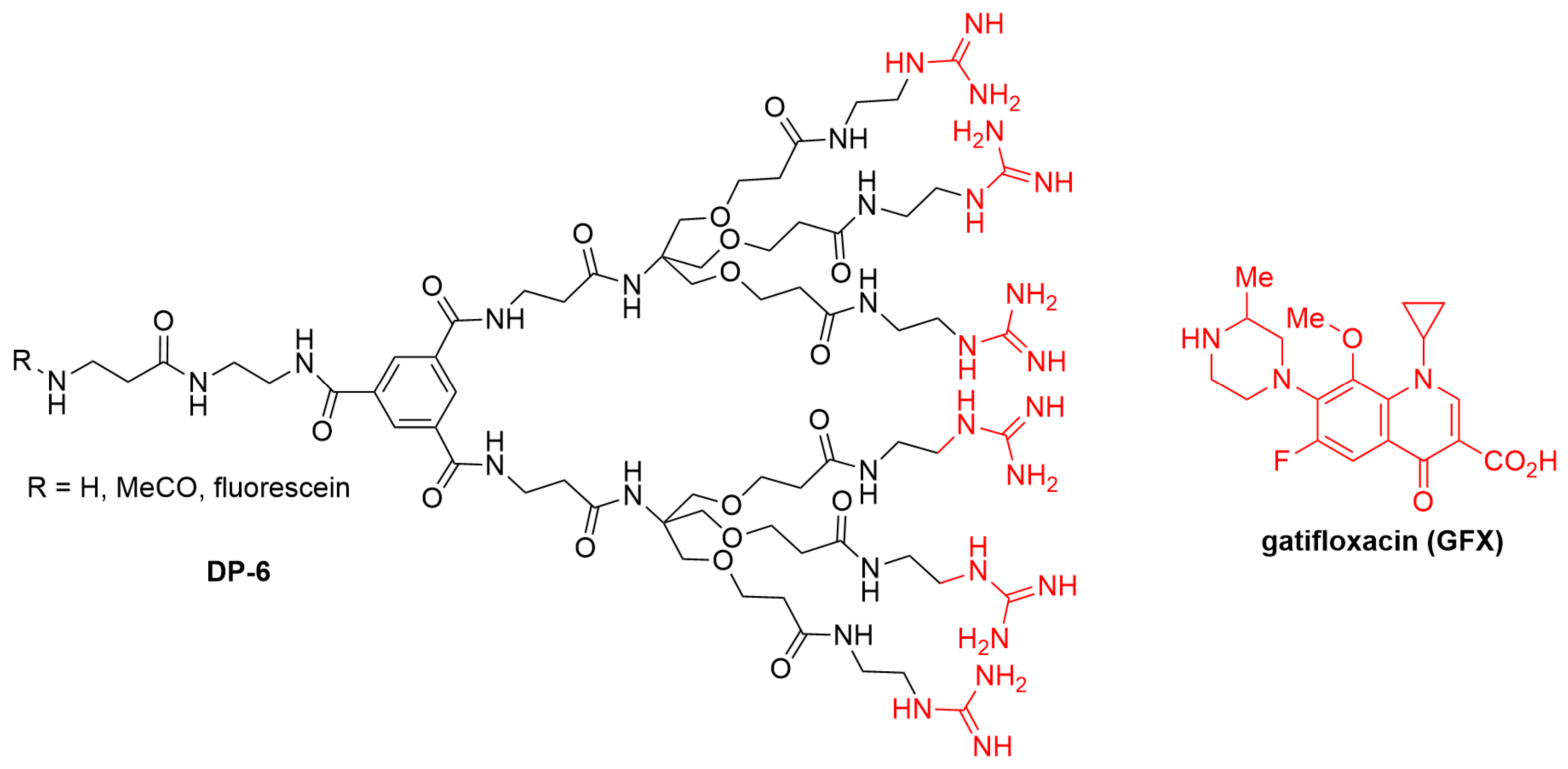
Dendron made of triolyl branches and six surface guanidine groups, used as a carrier for **GFX**.

A dendron with triolyl branches and six surface guanidine groups (**DP-6**) was used as a vehicle for gatifloxacin (**GFX**, Figure 13), an antibiotic for treatment of bacterial conjunctivitis caused by aerobic Gram-positive and -negative bacteria. Increased solubility of the drug in aqueous solution was observed. **DP-6/GFX** formulations were suitable for ocular administration in rabbits, in particular to determine the pharmacokinetic parameters, and also for determining the effect of multiple doses for 7 days (21 doses in total). A higher drug concentration in the conjunctiva was observed compared to free **GFX**, as well as in all the ocular tissues, demonstrating enhanced drug delivery efficiency with the dendron [97].

**Figure 14:**
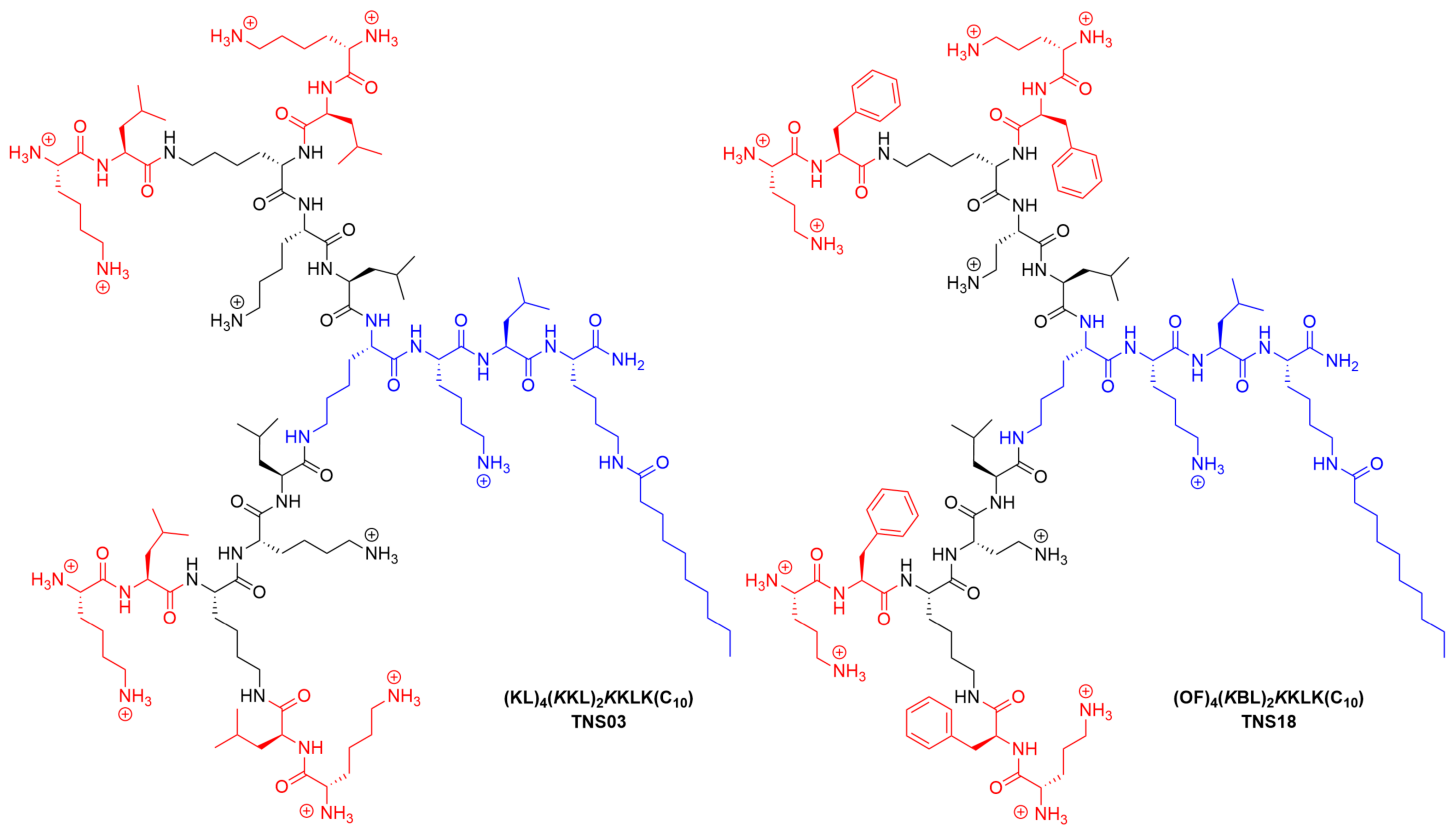
Generation-two dendritic peptides (O = ornithine; F = phenyl alanine; B = diaminobutyric acid; L = leucine; K = Lysine; *K* = branching Lysine; K(C_10_) = side-chain decanoylated Lysine). The core is in blue, the first generation in black, and the second generation in red.

A series of generation-two dendritic peptides bearing a fatty acid chain at the core was prepared by solid-phase peptide synthesis (two examples are shown in Figure 14). These dendrimers were tested against *P. aeruginosa*, *E. coli*, and *Bacillus subtilis*, and all of them were active. The compound bearing the C_10_ chain was the most efficient (**TNS03**). Then, a library of 1936 dendritic peptides was built using split-and-mix synthesis, out of which compound **TNS18** and two other dendritic peptides emerged as the most effective. Compound **TNS18** (acetate form) was used to treat mice intraperitoneally infected with a MDR *E. coli* strain. Only 20% of untreated mice survived, whereas 100% survived when treated with **TNS18**. This compound was better than a third-generation cephalosporin antibiotic, ceftriaxone (40% survival), and a carbapenem-class antibiotic, imipenem (80% survival) [98].

**Figure 15:**
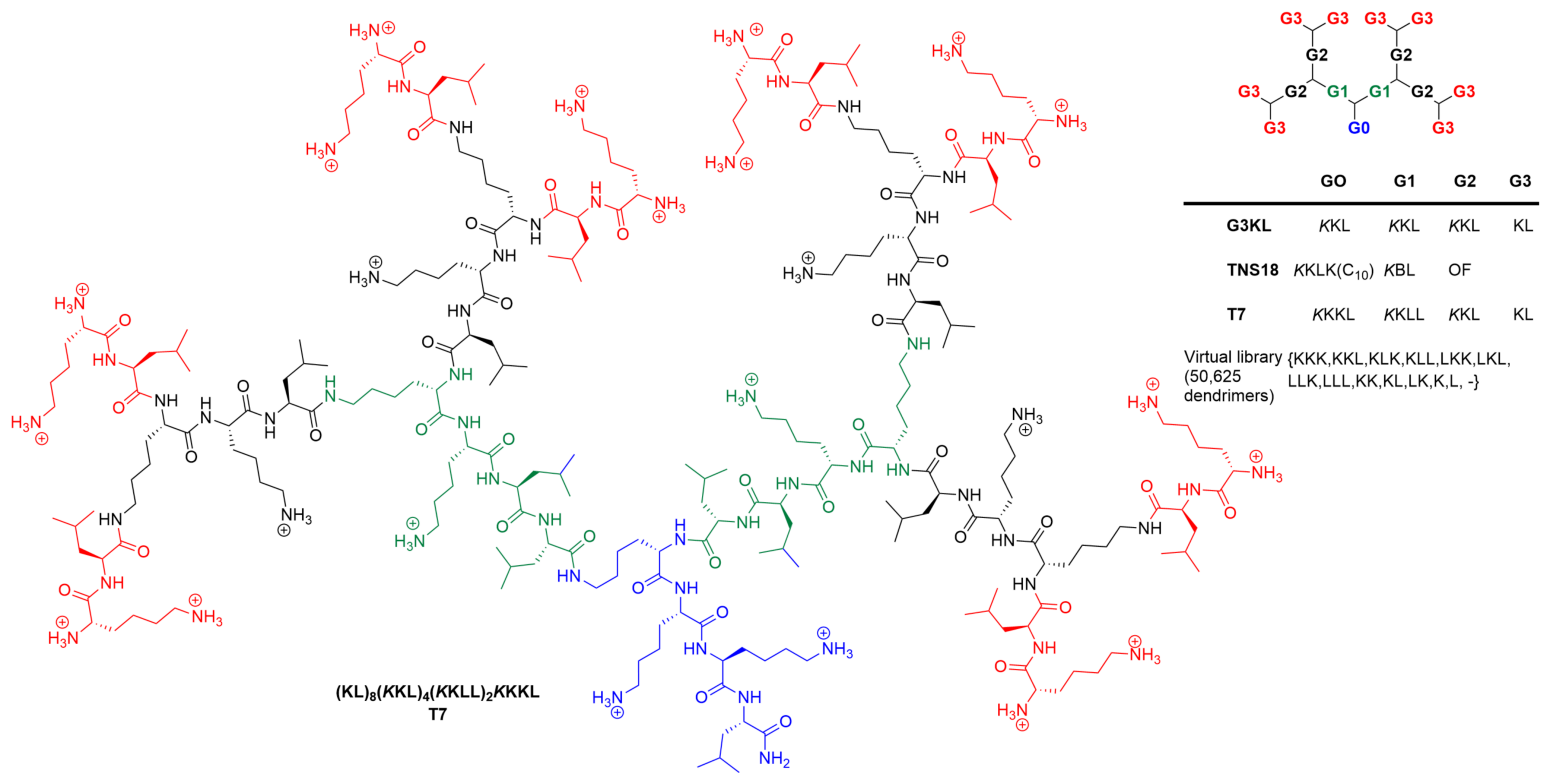
Third-generation peptide dendritic structures (core in blue, first generation in green, second generation in black, and third generation in red) and the principle of a virtual library of 50,625 dendrimers.

An even larger library of 50,625 dendritic peptides featuring all possible permutations of up to three residues of Lys or Leu in the branches was virtually designed, starting from dendrimer **G3KL**, which was previously shown to display antibacterial properties in vitro [99]. A few dendrimers from this virtual library were tested against *P. aeruginosa*, then against *K. pneumoniae* strains. Compound **T7** (Figure 15) was the only one that combined significant activity across all strains tested and negligible hemolysis. Both dendrimers **G3KL** and **T7** were tested in mice infected with a MDR *A. baumannii* strain. Surprisingly, dendrimer **G3KL** was totally inactive: all mice treated with this compound died after a few hours. On the contrary, 70% of the mice treated with **T7** were alive after 72 h [100]. Dendrimers **TNS18** and **G3KL** were tested in vivo in *G. mellonella* wax-moth larvae infected with *P. aeruginosa* strains, killing ≥ 97.5% of the larvae within 24 h. However, treatment with dendritic peptides did not provide any protection against *P. aeruginosa* infection. Mortality rates were comparable with the positive control (infected but not treated) [101].

**Figure 16:**
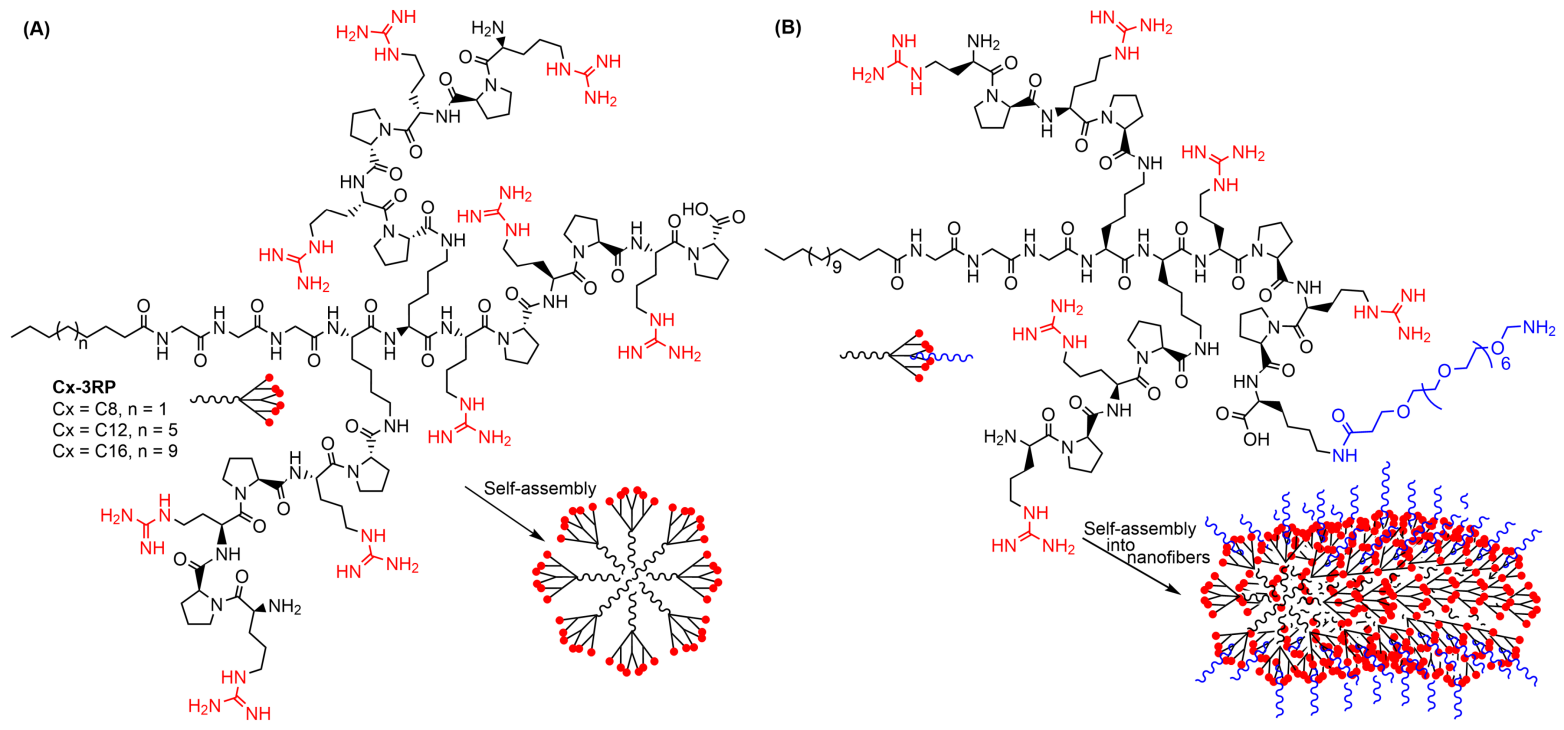
Peptide dendrons self-assembling in buffer. (**A**) Spherical nanoparticles. (**B**) Nanofibers.

Peptide dendrons equipped with a C8, C12, or C18 alkyl chain at the core and with two (**2RP**) or three (**3RP**) arms were shown to self-assemble in phosphate-buffered saline (PBS) to form spherical nanoparticles with diameters ranging from 5 to 45 nm (Figure 16A). Further aggregation into larger, irregular clusters was observed, leading to an average hydrodynamic diameter of approximately 302 nm, for the **C16-3RP** cluster, which was the most active compound against a range of bacteria, including *E. coli*, *P. aeruginosa*, *Salmonella enterica*, *Salmonella typhimurium*, *S. aureus*, *S. epidermis*, and *Enterococcus faecalis*. In vivo experiments were carried out with model mice injected with *E. coli* injection in the peritoneal cavity to induce acute peritonitis. One hour after infection, the mice were treated with 10 mg·kg^−1^ of **C16-3RP**. The treatment resulted in a perceptible reduction in the bacterial burden in the liver, spleen, lung, and kidney tissues. The levels of serum proinflammatory cytokines in the **C16-3RP**-treated group were significantly lower than those in the untreated group [102].

A related peptide dendron was functionalized with a hydrophilic poly(ethylene glycol) chain on the surface (Figure 16B). Due to the presence of a hydrophobic chain at the core and a hydrophilic chain on the surface, this compound self-assembled into nanofibers, which formed aggregates. These aggregates were tested in a peritonitis-sepsis mouse model (MDR *E. coli* infection). Unlike various conventional antibiotics, these peptide aggregates were efficient for treating this infection, as shown by a significant decrease in the bacterial burden in all tissues examined [103].

**Figure 17:**
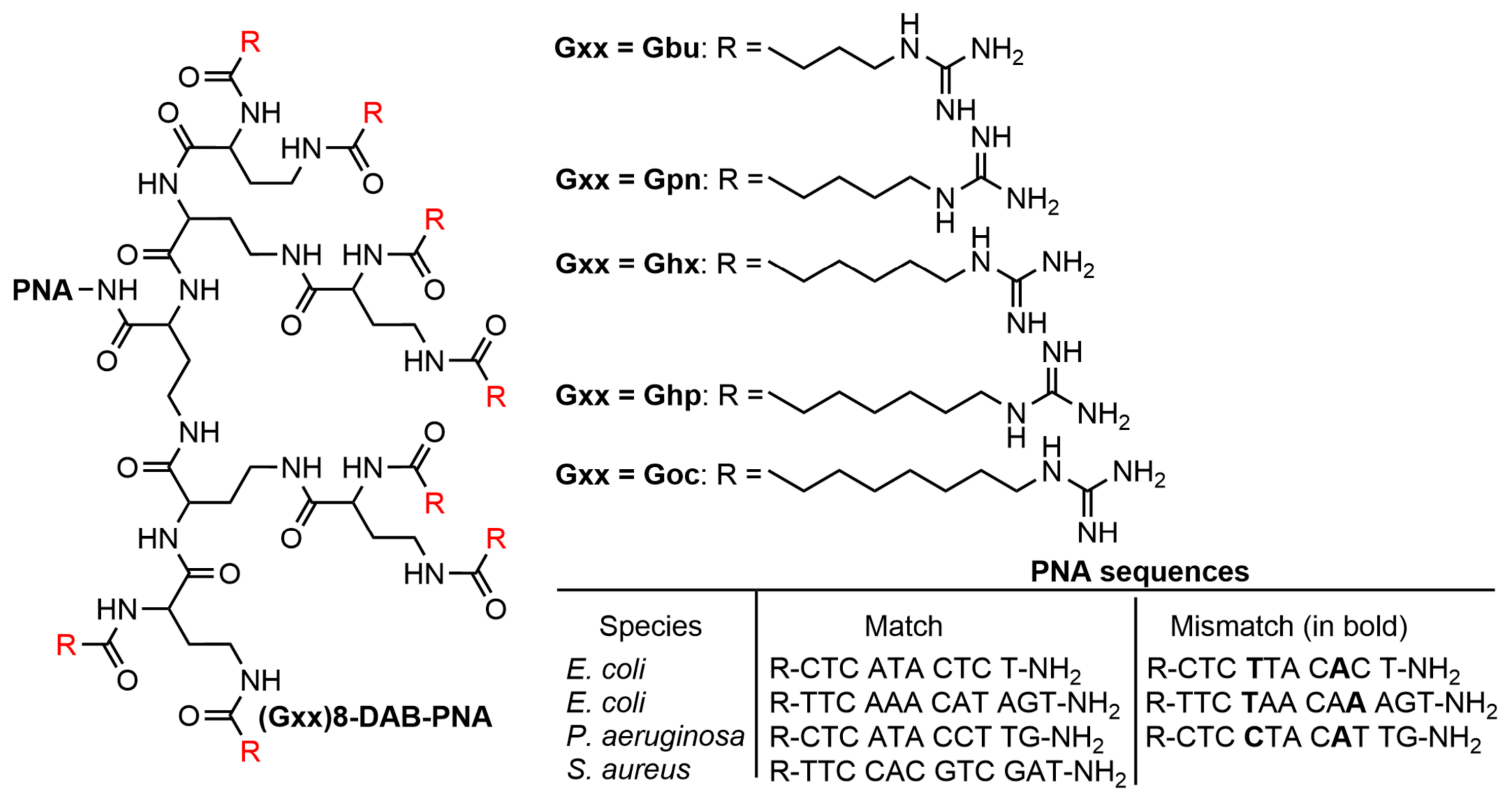
Non-natural peptide dendrons functionalized with antisense antibacterial peptide nucleic acids at the core (match or mismatch).

A series of peptide dendrons based on a non-natural amino acid (diaminobutanoic acid (**DAB**)), with antisense antibacterial peptide nucleic acids (**PNAs**) at the core, was chosen for targeting the translation start of the *acpP* mRNA, which codes for an essential gene required for fatty acid synthesis (match or mismatch). The surface was functionalized with eight moieties of guanidinobutanoic acid **(Gbu)8-DAB-PNA**, guanidinopentanoic acid **(Gpn)8-DAB-PNA**, guanidinohexanoic acid **(Ghx)8-DAB-PNA**, guanidinoheptanoic acid **(Ghp)8-DAB PNA**, or guanidinooctanoic acid **(Goc)8-DAB PNA** (Figure 17). The compound with the longest chain on the surface (**(Goc)8-DAB PNA**) was the most active against *E. coli* and *K. pneumoniae*. This compound was tested in vivo against MDR *E. coli* EC-106-09 in a neutropenic murine peritonitis model established by i.p. inoculation with *E. coli*. The mice were treated with 10 mg·kg^−1^ or 25 mg·kg^−1^ of either match or mismatch **(Goc)8-DAB PNA**. The match **PNA** was significantly more effective than the mismatch **PNA** in reducing the infection burden [104].

**Figure 18:**
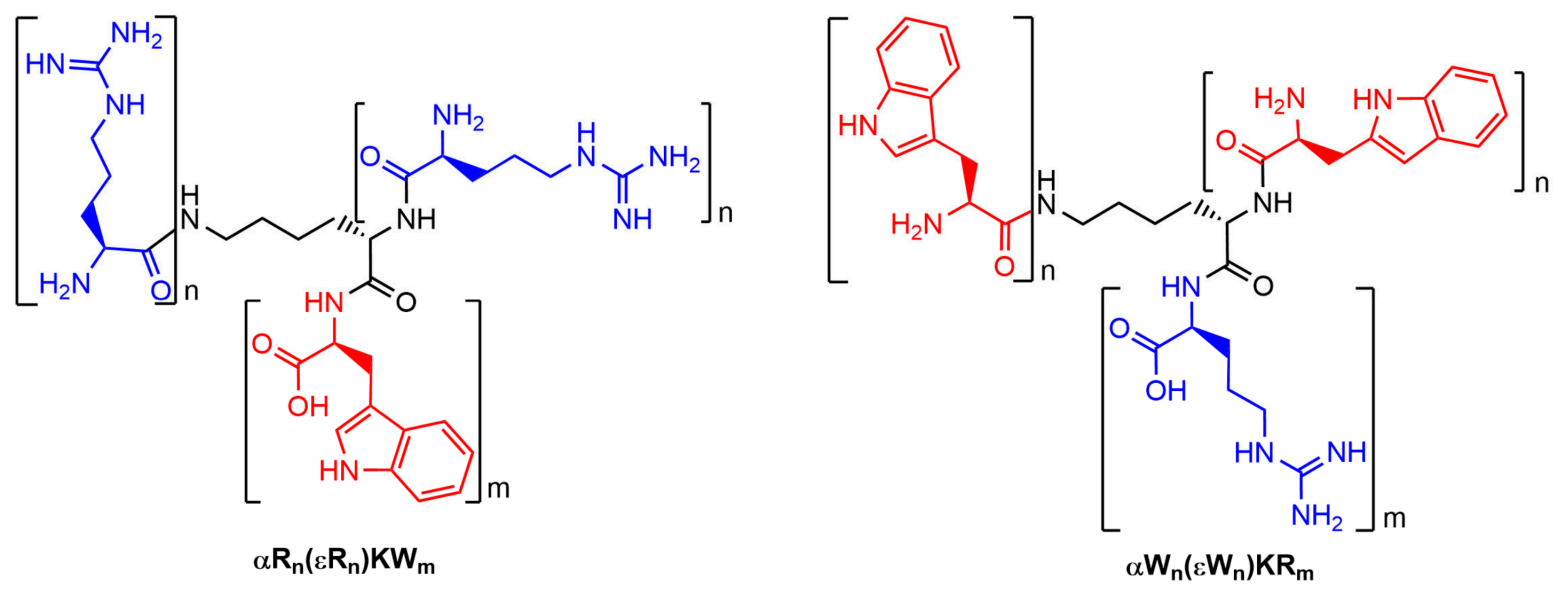
Two tri-branched peptides based on a Lysine core and composed of 4 to 14 amino acids, depending on the length of the branches.

Two tri-branched dendritic peptides (**αR_n_(εR_n_)KW_m_** and **αW_n_(εW_n_)KR_m_**) were synthesized from a Lysine core (K) using a combination of arginine (R) and tryptophan (W) (Figure 18). The n and m values were n = 1 for m = 1, 2, or 3; n = 2 for m = 3, 4, or 5; and n = 3 for m = 5, 6, or 7, thus affording 18 dendritic peptides composed of 4 to 14 amino acids. In vitro experiments against *S. aureus*, *B. subtilis*, *E. coli*, *K. pneumoniae*, *P. aeruginosa*, and *A. baumannii* showed that the dendritic peptide **R_2_(R_2_)KW_4_** was the most efficient. It was then tested in vivo in a murine pulmonary infection model induced by *E. coli* and compared with the efficacy of polymyxin B treatment. Mice that received the **R_2_(R_2_)KW_4_** peptide showed reduced infiltration of pneumonia cells and a little liver hemorrhage compared to those that received polymyxin B [105].

### 4.3. Overall Assessment of Peptide Dendrimers

Overall, peptide and poly(L-lysine) dendrimers are notable in this review primarily because they include **SPL7013**/VivaGel^®^/astodrimer, the most clinically advanced antimicrobial dendrimer described here. Other peptide dendrimers and dendrons broaden this family to include antimicrobial peptide-inspired compounds, amphiphilic derivatives bearing fatty-acid or alkyl chains, self-assembled nanostructures, and delivery systems. Several have shown efficacy in animal infection models, including models involving MDR Gram-negative bacteria, but the evidence remains heterogeneous and mostly preclinical. Further comparison across standardized infection models, administration routes, toxicity endpoints, and pharmacokinetic parameters will be needed to assess their therapeutic potential.

## 5. Miscellaneous Dendrimers

In addition to the two main families of dendrimers used for treating infections, PAMAM and peptide dendrimers, in particular poly(Lysine) dendrimers, many different types of dendrimers have been proposed for the same purpose. The following paragraphs concern poly(carbosilane) (CSi), poly(phosphorhydrazone) (PPH), poly(glycerol) (PGL), polyester (PED) dendrimers, and different types of dendritic–linear-dendritic compounds. 

### 5.1. Carbosilane Dendrimers (CSi)

**Figure 19:**
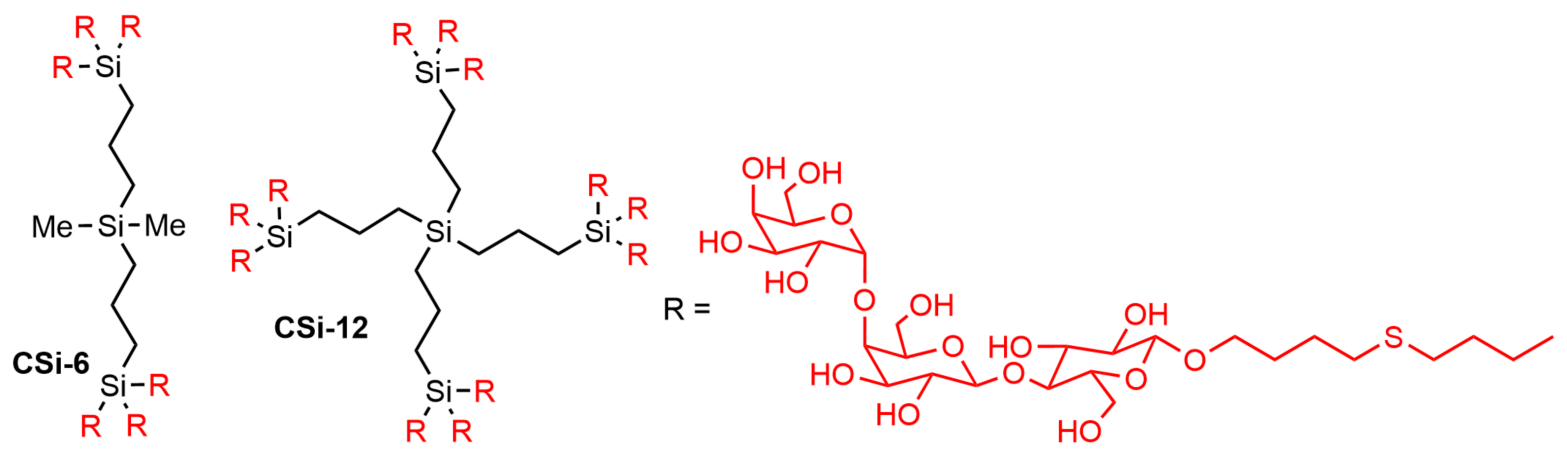
Carbosilane dendrimers functionalized with modified trisaccharides.

CSi dendrimers [106] are characterized by a hydrophobic internal structure, unlike PAMAM and peptide dendrimers. Very small CSi dendrimers were functionalized with modified trisaccharides (Figure 19) and were tested in mice infected with the Shiga toxin (Stx)-producing *E. coli* O157:H7, which causes fatal diarrhea and hemorrhagic colitis. The smaller compound (**CSi-6**) was the most efficient when administered intravenously to mice. Indeed, all mice treated with **CSi-6** survived more than 2 months without any symptoms, even at a dose of 5.0 μg·g^−1^ of body weight, and even when administered after establishment of the infection. On the contrary, none of the mice treated with **CSi-12** survived more than 7 days. It should be noted that this in vivo result contrasts with the in vitro results, where **CSi-12** inhibited Stx more effectively than **CSi-6** [107]. This discrepancy suggests that factors other than intrinsic Stx inhibition may influence in vivo efficacy. In this model, parameters such as biodistribution, tissue penetration, toxin accessibility, clearance, steric constraints, or aggregation may have contributed to the superior protection observed with **CSi-6**. The smaller **CSi-6** compound may therefore have reached or neutralized the relevant toxin more efficiently in vivo than **CSi-12**, despite its lower Stx-inhibitory activity in vitro. This example highlights the limits of extrapolating directly from in vitro potency to in vivo efficacy, since dendrimer size, architecture, and multivalency may influence not only target binding, but also biodistribution, accessibility to the toxin, clearance, and overall biological performance in a living organism.

### 5.2. Poly(phosphorhydrazone) (PPH) Dendrimers

**Figure 20:**
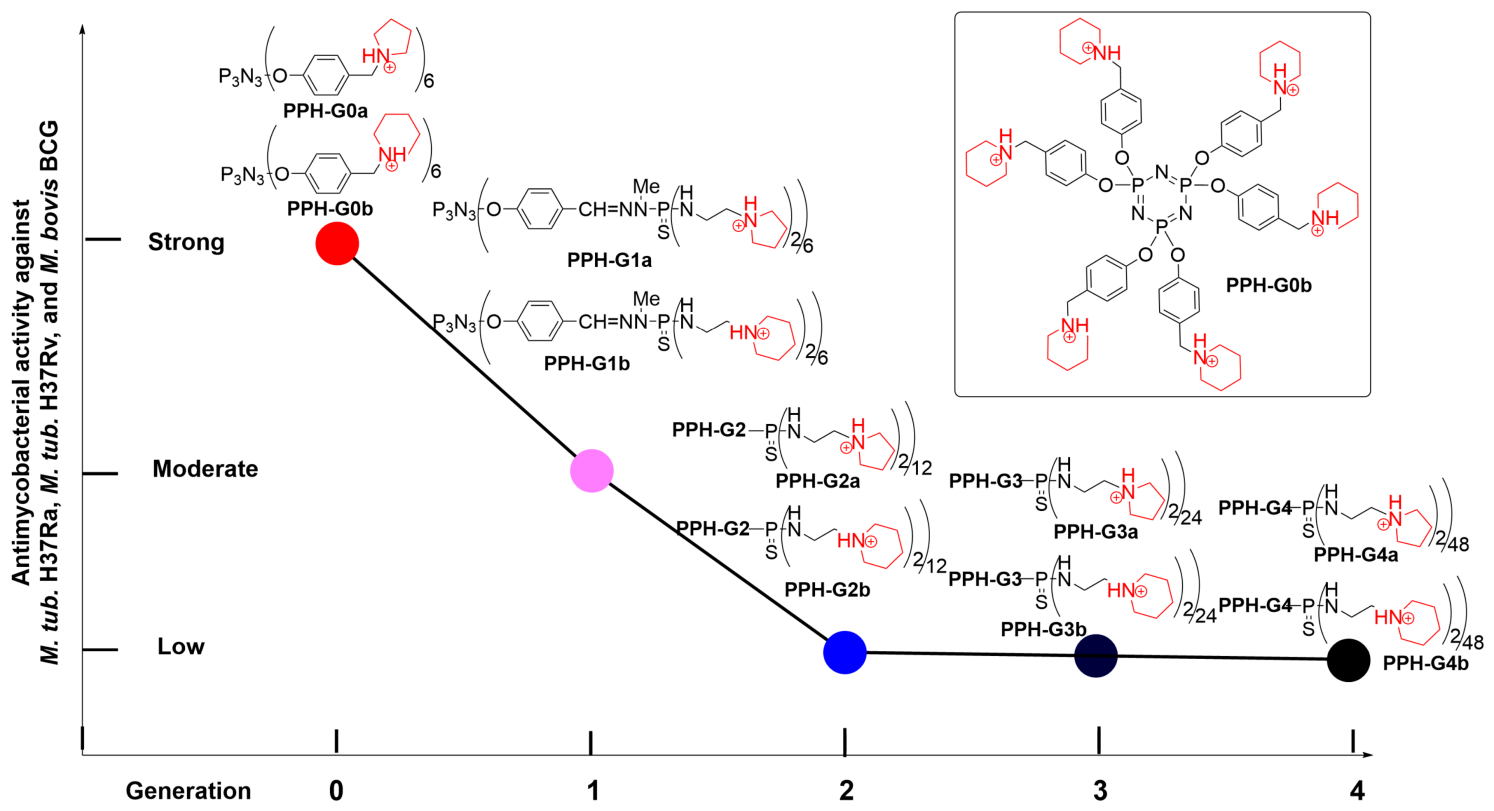
Influence of the generation of PPH dendrimers on efficacy against tuberculosis.

PPH dendrimers [108] have also a hydrophobic internal structure, like CSi dendrimers. Among the numerous properties of PPH dendrimers [109], one can cite their effectiveness against *M. tuberculosis*. Different generations of PPH dendrimers were synthesized and functionalized on their surface with different types of cyclic ammoniums. These dendrimers were tested in vitro against three bacterial strains: attenuated *M. tuberculosis* H37Ra, virulent *M. tuberculosis* H37Rv, and *Mycobacterium bovis* BCG. A negative dendritic effect was observed, ongoing from generation zero to generation four. Only the most active compounds are shown in Figure 20. As compound **PPH-G0b** had a much better safety index (16.02) than compound **PPH-G0a** (3.1), it was selected for in vivo tests in mice infected with the H37Ra strain of *M. tuberculosis*. Compound **PPH-G0b** administered orally very significantly reduced the mean bacterial count in the lungs of infected mice, with a greater efficacy than that observed with rifampicin (**RIF**) and ethambutol (**ETB**), two reference drugs used against tuberculosis [110]. The same compound was tested against drug-resistant mycobacterial strains in combination with multiple classes of antibiotics, with the aim of potentiating their activity. A murine neutropenic bacteremia model was used for testing different combinations of antibiotics with **PPH-G0b** against non-tubercular mycobacterial pathogens. The use of **PPH-G0b** alone or in combination with the drugs (rifampicin, bedaquiline, linezolid, or clofazimine) led to a highly significant reduction in bacterial counts in all organs as compared with any of the individual treatments [111].

**Figure 21:**
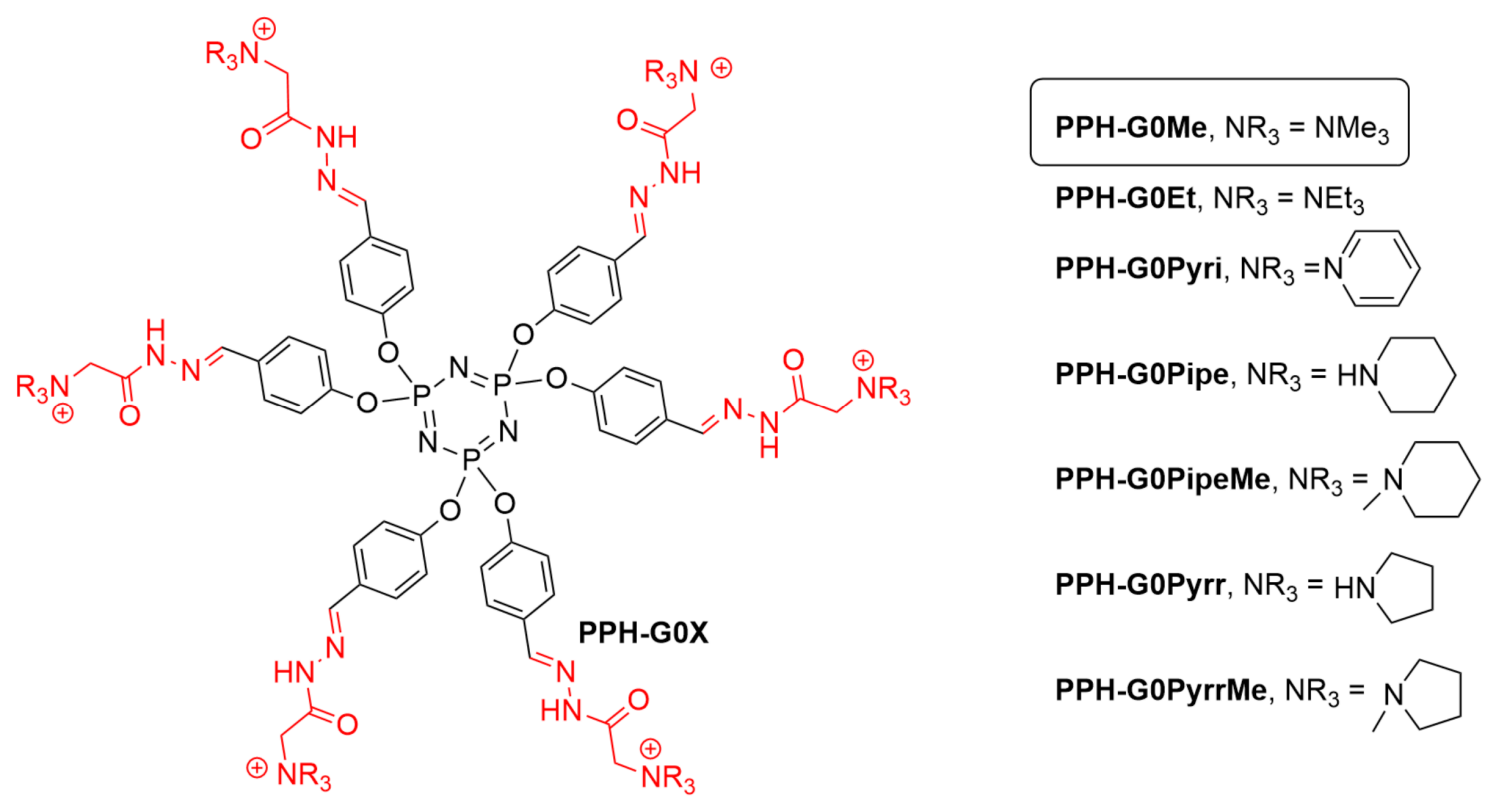
Generation-zero PPH dendrimers functionalized with different types of acetyl hydrazones.

A series of small (generation zero) PPH dendrimers functionalized with acetyl hydrazide derivatives of trimethyl ammonium (Girard T reagent), triethyl ammonium, pyridinium (Girard P reagent), piperidinium and methyl piperidinium, or pyrrolidinium and methyl pyrrolidinium (Figure 21) was synthesized. These compounds were tested against *S*. *aureus* and *Enterococcus* species. Compound **PPH-G0Me** was efficient and exhibited the highest selectivity index (>62.5); thus, it was tested in vivo. A murine skin model of infection with gentamicin-resistant *S*. *aureus* NRS119 was used for testing **PPH-G0Me** alone or associated with gentamicin. It was shown that the combination had better efficacy than the individual components used separately (**PPH-G0Me** and gentamicin) [112].

### 5.3. Poly(glycerol ether) (PGL) Dendrimers

**Scheme 8:**
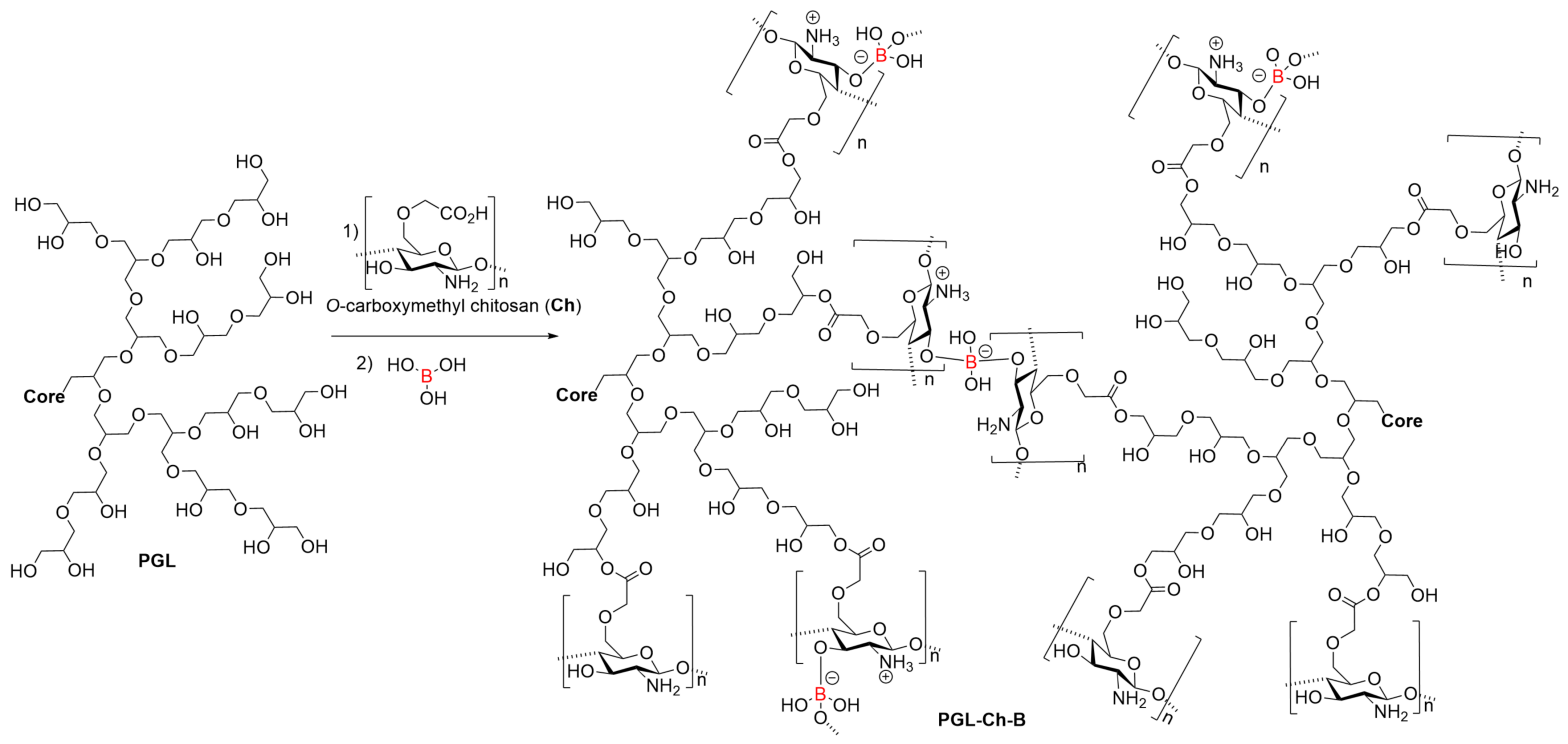
Schematized polyglycerol dendritic structures associated with chitosan and boric acid.

PGL dendrimers are hyperbranched polymers that can be prepared by a controlled synthetic approach. The first synthesis was based on the use of latent ABm monomers, wherein B-groups were set free only upon reaction of the A group [113], but several other methods have been proposed and reviewed [114]. Despite being less rigorously defined than classical dendrimers, PGL dendrimers (proposed structure in Scheme 8, left part) have many biomedical applications, as they are composed of a stable and biocompatible polyether scaffold [115]. They were covalently associated with *O*-carboxymethylated chitosan. The reaction of such materials with boric acid for improving biocompatibility [116] induced a marked increase in the bulk viscosity of **PGL-Ch-B**. Antibacterial studies against *S. aureus* and *P. aeruginosa* showed significant suppression of bacterial proliferation, due to the delivery of boron and the cationic nature of **PGL-Ch-B**. This material was used to prepare artificial membranes, which were used for coating wounds on female rats. Histological evaluations were performed to assess tissue response to the membranes used and progression of healing at Days 3, 7, 15, and 30. Complete healing was observed in all cases at 30 days post-operation, and the degradation of the membrane was complete (no evidence of fragments of the **PGL-Ch-B** membranes was found) [117].

**Figure 22:**
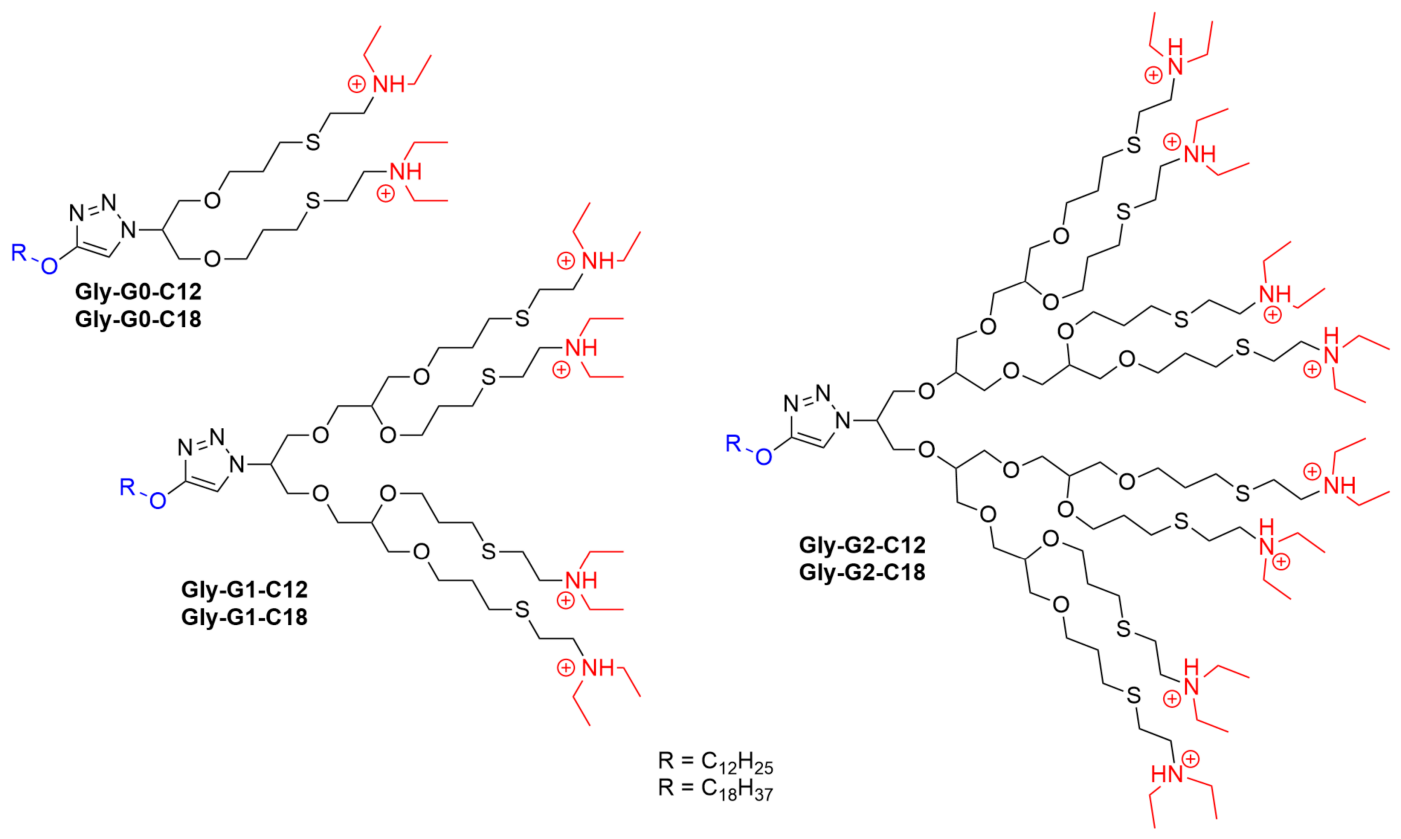
Cationic PGL dendrons with either a C12 or C18 chain at the core.

Another example of PGL dendritic structure concerned six cationic dendrons with either a C12 or a C18 chain linked to the core via a CuAAC reaction, which were used as surfactants (Figure 22). These dendrons were tested against Gram-positive (MRSA) and Gram-negative (*E. coli*) bacteria, including drug-resistant strains, as well as for preventing biofilm formation and eradicating pre-existing biofilms. The most effective compound in all tests was **Gly-G1-C12**, which was then tested in vivo in wounds infected with MRSA to evaluate its ability to inhibit wound infections in mice. It was found that optimal collagen recovery and accelerated tissue healing were induced by compound **Gly-G1-C12**. The **Gly-G1-C12** dendron induced the fastest recovery (faster than vancomycin), achieving complete wound closure by Day 14. The survival rate was 100% for the treated mice, but also for those which were not treated [118].

### 5.4. Poly(ester) Dendrimers (PEDs)

**Figure 23:**
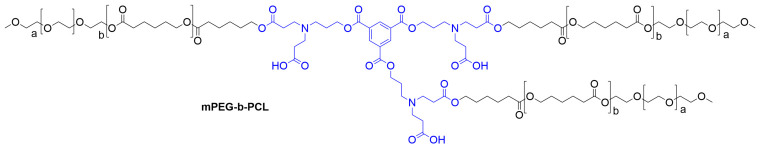
The star polymer **mPEG-b-PCL** created from a first-generation dendrimer (in blue) for drug delivery.

Unlike glycerol ether derivatives, which are stable compounds, the main properties of PEDs are their hydrolytic instability and high biodegradability, resulting in inherent low toxicity and good biocompatibility [119]. A first-generation poly(ester-amine) dendrimer was functionalized with three arms composed of diblock copolymer of methoxy poly(ethyleneglycol)-b-poly(ε-caprolactone) (**mPEG-b-PCL**) (Figure 23). This star polymer formed nanovesicles suitable for efficient drug (vancomycin) delivery. The encapsulation rate inside the nanovesicles was 76.49 ± 2.4%. The proportion of vancomycin released from the drug-loaded nanovesicles was 65.8% over a period of 48 h. The drug-loaded nanovesicles exhibited 8- and 16-fold lower minimum inhibitory concentrations (MICs) against methicillin-sensitive and methicillin-resistant *S. aureus* strains compared to free vancomycin. A murine model of in vivo skin infection showed a 20-fold reduction in MRSA load in groups treated with the drug-loaded nanovesicles compared to free vancomycin. This was proof that the nanovesicles enhanced the activity of vancomycin. The mice with untreated skin displayed evidence of tissue inflammation and abscess formation [120].

**Figure 24:**
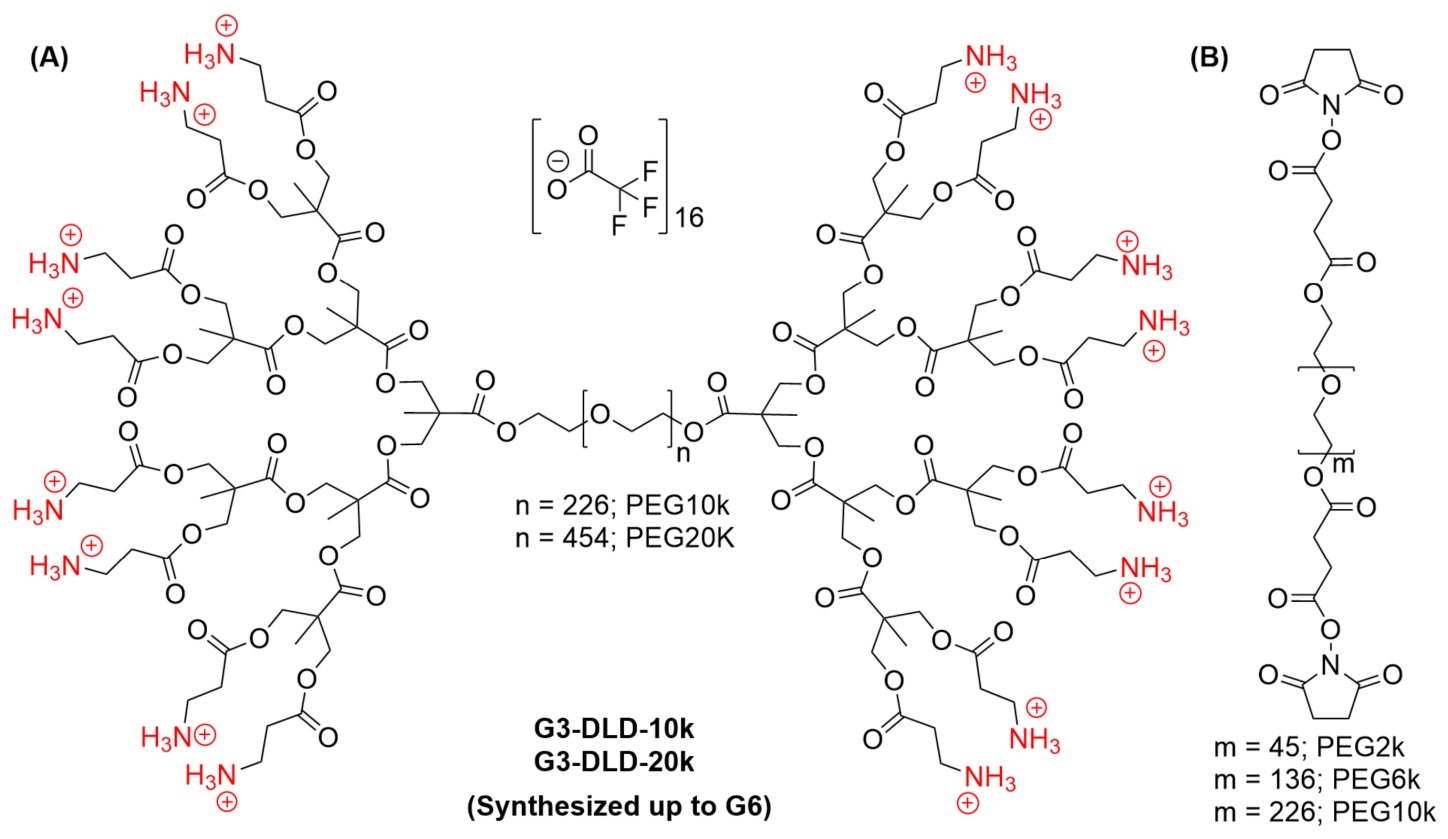
(**A**) Polyester dendrimer (G3) built on a PEG core. (**B**) NHS-PEG cross-linker.

A series of dendritic–linear-dendritic (**DLD**) hybrids based on a PEG core (PEG10k or PEG20k, molecular weights of 10 or 20 kg·mol^−1^) and polyester branches was synthesized from generation one to generation six, having from 4 to 128 ammonium groups on the surface, based on β-alanine (Figure 24A). The antibacterial properties of the amine-functionalized PEG20k-based **DLDs** were evaluated against different bacterial strains. Generation six (**G6-DLD-20k**) was found to be the most effective against *E. faecalis*, *E. coli*, *P. aeruginosa*, *S. aureus*, MRSA, *S. epidermidis*, and *Propionibacterium acnes* (now *Cutibacterium acnes*) at a minimum microbicidal concentration (MMC) of around 1 μM and a MIC below 1 nM. These dendrimers can be cross-linked using *N*-hydroxysuccinimide (NHS) PEG-based cross-linkers (Figure 24B) of type PEG2k, PEG6k, or PEG10k, used in different molar ratios, to form hydrogels. This cross-linking process was carried out in an in vivo setting using a two-component syringe delivery system to generate the hydrogels. Statistically, 4, 8, 16, and 32 (n) amines out of the total 128 for **G6-DLD-20k** were used for cross-linking. A cross-linking density of 8 out of the 128 amines was determined as optimal. Studies of the hydrogel degradation demonstrated that the ester bonds were primarily degraded by a hydrolytic rather than an enzymatic process. The dual syringe was used to induce the formation of hydrogel for surgical site application in an in vivo animal mouse model of an *S. aureus*-infected suture. An 80% reduction in the number of bacteria was observed 4 h post-infection, and a 90% reduction 6 h post-infection, in vivo [121].

### 5.5. Other Types of Dendrimers

**Figure 25:**
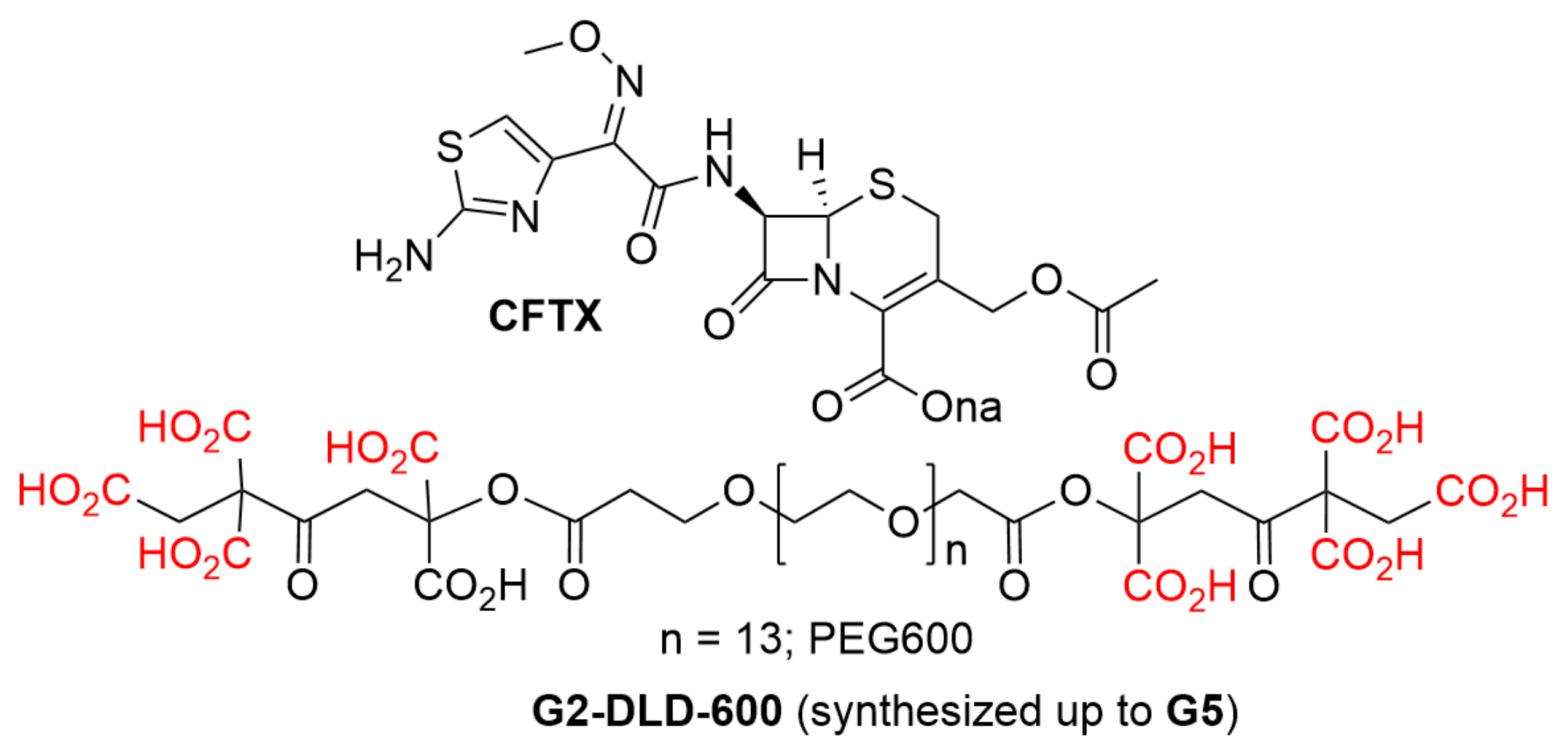
Different generations of **DLD** used to encapsulate the **CFTX** antibiotic.

Another type of **DLD** hybrid was prepared by linking, to a PEG600 core, dendrimers made of citric acid derivatives, from generation one to generation five (**G2-DLD-600,** Figure 25). These **DLDs** were used for the encapsulation of cefotaxime sodium (**CFTX**) and tested against *S. aureus*, *P. aeruginosa*, *K. pneumoniae*, and *Haemophilus influenzae* to assess the antibacterial activity. **G5-DLD-600-CFTX** exhibited a 92.4% encapsulation efficacy and displayed an 83.8% drug release in 48 h, along with a synergistic effect between **CFTX** and the hyperbranched structure. Pharmacokinetic investigations were conducted by intramuscular administration to rats, but no antimicrobial test was carried out in vivo [122].

**Figure 26:**
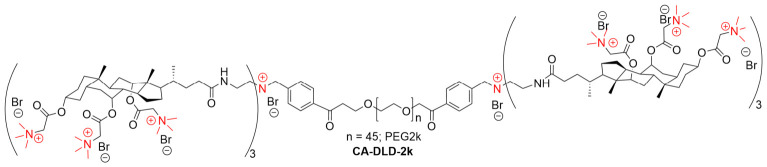
**DLD** functionalized with ammoniums derivatives of cholic acid.

A related example of **DLD** concerned PEG-2k functionalized at each end with three branches composed of amphiphilic cholic acids (**CAs**) modified with quaternary ammoniums (Figure 26). This compound self-assembled into nanostructured micelles in aqueous solution and displayed potent antibacterial activity against *S. aureus* and *E. coli*, with MIC values of 7.50 and 7.79 μM, respectively. In vivo studies were performed with mice that had two wounds with a diameter of 10 mm on their backs infected with *S. aureus*. It was shown that **CA-DLD-2k** injected inside wounds 24 h after infection was much more effective and safer than penicillin G in treating *S. aureus* infection and promoted more rapid wound healing. Furthermore, evaluation of toxicity in the main organs proved the excellent biosafety of **CA-DLD-2k** [123].

**Figure 27:**
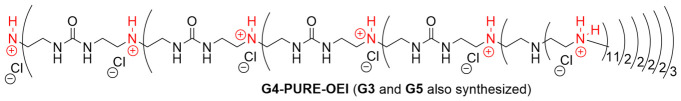
Poly(urea) dendrimer (generation four) functionalized with oligo(ethyleneimine).

The periphery of poly(urea) (**PUREs**) dendrimers [124] was functionalized with 48 linear oligoethyleneimines (**OEIs**). The surface and the internal structure were easily protonated, affording a highly cationic dendritic structure of generations three, four, and five (**G4-PURE-OEI** in Figure 27). Compounds **G3-PURE-OEI and G4-PURE-OEI** were tested against a collection of both Gram-positive (*S. aureus* MRSA, *Streptococcus pneumoniae*) and Gram-negative (*P. aeruginosa*, *A. baumannii*, carbapenem-resistant *K. pneumoniae* and *S. enterica*, *Burkholderia cenocepacia*) bacteria and fungi (*C. albicans* and *Candida glabrata*; for a review about the use of dendrimers against fungi, see [125]). Generation four displayed highly efficient antimicrobial and anticandidal activity and was tested in vivo on *G. mellonella* larvae infected with a lethal dose of *S. aureus* or *P. aeruginosa*. It was shown that **G4-PURE-OEI** clearly improved larva survival [126].

### 5.6. Comparative Analysis of Miscellaneous Dendrimers

The miscellaneous dendrimer families discussed above should not be considered as a homogeneous group. Carbosilane dendrimers illustrate the potential of hydrophobic scaffolds for multivalent toxin neutralization, but the available in vivo evidence remains limited and indication-specific. Phosphorhydrazone dendrimers are interesting for antimycobacterial activity and antibiotic potentiation; however, despite encouraging early in vivo results in mouse models, they remain at a very early preclinical stage, with limited pharmacokinetic, biodistribution, safety, and comparative efficacy data. Polyglycerol- and polyester-based systems offer attractive biocompatibility or biodegradability features and are often used in hydrogels, membranes, or coatings, where antimicrobial and regenerative effects may be combined. However, in such systems, the contribution of the dendrimer itself to direct antimicrobial efficacy can be difficult to separate from wound-healing, drug-release, or material-related effects. Poly(urea) dendrimers show broad antimicrobial potential, but the in vivo evidence is still limited to early infection models. Thus, these platforms broaden the chemical diversity of antimicrobial dendrimers, but their translational maturity remains highly variable.

## 6. Conclusions

Dendrimers have demonstrated significant potential as antimicrobial agents, both independently and as nanocarriers for existing drugs, which can be either encapsulated within the dendritic structure or covalently attached to its periphery, offering a promising strategy to combat antibiotic resistance. In vivo studies spanning diverse animal models —from larvae to macaques, most commonly mice and rats—have underscored their efficacy. These studies cover a broad range of infection-related models, including bacterial vaginosis, wound and skin infections, dental and periodontal infections, pulmonary infections, tuberculosis, biofilm-associated infections, and selected viral infection models. To facilitate comparison among the different dendrimer families, Table 2 summarizes representative antimicrobial dendrimer-based systems for which in vitro activity was reported and further evaluated in vivo. The table highlights the active role of each system, the main in vitro target or activity, the in vivo model, the route of administration, and the principal biological outcome. The activity can arise from different mechanisms, including membrane disruption, multivalent interactions with microbial or toxin targets, antibiofilm effects, delivery of antibiotics or antimicrobial peptides, release of gaseous mediators such as NO, CO, or SO_2_, and modulation of the local inflammatory environment.

Despite these promising results, comparison across studies remains difficult. The reported systems differ widely in dendrimer scaffold, generation, surface chemistry, payload, formulation, route of administration, dosing regimen, infection model, and biological endpoint. In addition, some studies provide clear evidence of antimicrobial efficacy in infected animal models, whereas others mainly report pharmacokinetic, biodistribution, local tolerance, or wound-healing outcomes. Future work would benefit from more standardized in vivo models, systematic toxicity and pharmacokinetic studies, and direct comparison with clinically relevant antimicrobial treatments. This last point remains particularly important, because some studies compare dendrimer-based systems with antibiotics or reference treatments, whereas others evaluate efficacy without a standard-of-care control. Such comparisons are needed to determine whether dendrimers offer a true therapeutic advantage rather than only activity in isolated experimental models.

This heterogeneity also prevents any simple ranking of dendrimer families according to therapeutic performance. Across dendrimer families, the current evidence does not allow definitive classification of platforms according to efficacy/safety ratio, manufacturability, or biodegradability. Nevertheless, several broad trends can be identified. PAMAM dendrimers are the most extensively explored and highly versatile, but cationic derivatives raise recurring toxicity concerns. Poly(L-Lysine) dendrimers, especially astodrimer, provide the clearest clinical precedent, largely in locally administered formulations. Peptide dendrimers and amphiphilic dendrons show strong discovery-stage antimicrobial potential, particularly against resistant bacteria, but remain heterogeneous in design and evaluation. Polyester and polyglycerol-based systems may offer advantages in biodegradability or biocompatibility, especially for hydrogels and coatings, whereas carbosilane and phosphorhydrazone dendrimers remain promising but less clinically advanced. Thus, the optimal platform depends on the intended indication, route of administration, payload, local versus systemic exposure, and required safety profile.

Establishing clear structure–activity relationships for antimicrobial dendrimers remains challenging. Even in classical small-molecule QSAR, closely related analogs can display unexpectedly large differences in biological activity, a phenomenon known as “activity cliffs” [127,128]. This limitation may be even more pronounced for dendrimer-based systems, where biological performance depends not only on the chemical structure, but also on macromolecular aspects such as dendrimer-specific parameters, including generation, surface charge, multivalency, architecture [129], functionalization density, aggregation state, formulation, payload loading, release behavior, and the way these parameters interact with the infection model used. Therefore, the available studies do not support definitive predictive SAR rules. Several structure–activity trends nevertheless emerge from the systems reviewed here. Cationic surfaces often favor interaction with bacterial membranes and can provide strong antimicrobial activity, but they may also increase cytotoxicity and safety concerns. Neutral or anionic dendrimers generally show improved tolerability but often require additional functionalization, payload delivery, or multivalent interactions to achieve strong antimicrobial effects. Amphiphilic dendrons bearing hydrophobic chains can mimic antimicrobial peptides and promote membrane insertion, but their in vivo behavior depends on aggregation, formulation, and route of administration. Higher generation is not necessarily associated with better activity, as illustrated by examples in which smaller dendrimers or lower generations performed better in vivo. These trends should be interpreted cautiously, because they arise from heterogeneous studies rather than from systematic head-to-head comparisons.

Consistent with these limitations, clinical translation remains limited. To date, only a small number of dendrimer-based anti-infective systems have reached the stage of clinical evaluation. Astodrimer/SPL7013 provides the clearest example of successful translation, probably because it combines local administration, limited systemic exposure, a polyanionic surface associated with favorable local tolerability, a favorable safety profile, relatively simple topical formulations, and clinically accessible indications such as bacterial vaginosis and topical antiviral prevention. PAMAM-NAC represents a different clinical example, in which a dendrimer nanotherapy was evaluated in the context of severe infection-associated inflammation rather than direct antimicrobial activity. By contrast, most other dendrimer families, including peptide, carbosilane, phosphorhydrazone, polyglycerol, polyester, and poly(urea) dendrimers, remain mainly preclinical.

Overall, dendrimers offer a versatile and chemically tunable platform for antimicrobial and anti-infective applications. Their value lies not only in intrinsic antimicrobial activity, but also in multivalent pathogen or toxin binding, antibiofilm activity, drug delivery, local formulation, and modulation of infection-associated inflammation. However, their future development will require better-defined infection models, appropriate clinical comparators, systematic safety and biodistribution studies, clearer but cautiously interpreted structure–activity trends, and reproducible formulations. For complex formulations involving partial functionalization, supramolecular assembly, drug encapsulation, hydrogels, coatings, or hybrid nanomaterials, translation will also require robust physicochemical characterization and quality control, including batch-to-batch reproducibility, degree of substitution, purity, size distribution, payload loading, release kinetics, sterility, and stability, to name a few. Safety remains particularly important for highly cationic architectures, for which hemolysis, membrane disruption, cytokine release, complement activation, organ accumulation, and long-term clearance should be more systematically evaluated. Under these conditions, dendrimer-based systems may continue to provide useful tools for difficult-to-treat infections, particularly when their architecture, route of administration, and safety profile are matched to a well-defined clinical indication.

## Figures and Tables

**Table 1:** Main clinical and advanced translational studies involving SPL7013/astodrimer.

Dendrimer/Formulation	Indication/Study Aim	Study Type/Phase	Subject or Model	Main Outcome	Reference
**SPL7013** vaginal gel	HSV/HIV prevention	Preclinical studies	Mouse, guinea pig, and macaque models	Protection against HSV or SHIV transmission in relevant models	[66,67,68,69,70]
**SPL7013** penile gel	Male genital tolerability	Phase 1	Healthy men	Acceptable local safety/tolerability	[71,72,73]
**SPL7013** vaginal gel	Vaginal safety	Phase 1	Healthy women	Local tolerability evaluated, with mild irritation reported in some studies	[74,75,76,77,78,79,80]
**SPL7013** vaginal gel	Bacterial vaginosis	Phase 2/3	Women with bacterial vaginosis	Superior efficacy versus placebo and good tolerability	[81,82]
**SPL7013** vaginal gel	Bacterial vaginosis	Phase 3	Women with recurrent bacterial vaginosis	Reduced recurrence versus placebo	[83]
**SPL7013** nasal spray	SARS-CoV-2 infection	Preclinical mouse and Phase 1/2 studies	Mouse model, healthy volunteers, and non-hospitalized adults with SARS-CoV-2 infection	Well tolerated and reduced viral load in the clinical study	[86,87,88]

**Table 2:** Representative antimicrobial dendrimer-based systems, the main in vitro target or activity, the in vivo model, the route of administration, and the principal biological outcome.

Dendrimer Family	Dendrimer/System	Active Role	Main In Vitro Target/Activity	In Vivo Model	Route/Administration	Main In Vivo Outcome	Ref.
**PAMAM**	**PAMAM G4-OH**	Intrinsic antibacterial activity	Activity against *E. coli*; lower cytotoxicity than **PAMAM G4-NH_2_**	Pregnant guinea pig model of intra-cervical *E. coli* infection/chorioamnionitis	Topical cervical application	No bacterial growth detected in amniotic fluid samples after treatment	[31]
**PAMAM**	**PAMAM G5-NH_2_-DOP** coating	Antimicrobial and osteogenic surface coating	Antimicrobial activity on titanium alloy surfaces	Rat Ti6Al4V tibial implant model with *S. aureus*-infected implants	Implant coating	Improved antimicrobial performance and long-term implant stability	[32]
**PAMAM**	**PAMAM G3-BN**	Antibacterial and enamel-preserving dendrimer	Reduced adhesion, viability, and biofilm formation of *S. mutans* and *S. sanguinis*	Rat dental caries model with human enamel blocks	Topical oral application	Reduced lesion depth and improved enamel remineralization	[33]
**PAMAM**	**TDZ-G4@CA** (**PAMAM-G4-NH_2_-TDZ/CA-Tyr**)	Antibiotic delivery/antibiofilm system	MRSA biofilm disruption and pH/lipase-responsive release of D-Tyr and tedizolid	Mouse MRSA subcutaneous abscess model	I.v. administration	Accumulated at infected site and induced strong bacterial reduction	[34]
**PAMAM**	**PAMAM-G4-NH_2_-AZM/DA-PEG-PLys**	pH-responsive antibiotic delivery	Biofilm penetration and azithromycin release in acidic biofilm microenvironment	Mouse chronic *P. aeruginosa* lung infection	I.v. administration	Reduced bacterial burden and inflammatory markers	[35]
**PAMAM**	**PAMAM-PEG** hydrogel	Local hydrogel/antimicrobial delivery platform	Sustained antibiotic release and intrinsic antibacterial activity expected from amine-terminated PAMAM	Pregnant guinea pig vaginal tolerance model	Local intravaginal insertion	Well tolerated and localized on the superficial mucified epithelial layer; no infected in vivo efficacy model reported	[36]
**PAMAM dendrimer**	β-cyclodextrin-graft-**PAMAM-NO**	NO delivery/antibiofilm system	Inhibition and dispersion of *S. aureus* biofilm	Rat chronic rhinosinusitis model	Intranasal spray	Improved symptoms and therapeutic effect against *S. aureus* infection	[41]
**PAMAM dendron**	**C18-G1**	Intrinsic antibacterial amphiphilic dendron	Activity against *S. aureus*, *E. coli*, and resistant strains	Mouse MRSA-infected wound model	Local wound treatment	Accelerated wound healing; optimal healing rate at Day 7	[42]
**PAMAM dendron**	**C18-G3-NH_2_**	Intrinsic antibacterial amphiphilic dendron	Membrane insertion and lysis of bacteria	Mouse acute pneumonia and bacteremia models with *P. aeruginosa*	I.p. administration	Effective at lower therapeutic concentrations than reference antibiotics in the reported models	[44]
**PAMAM dendron**	**C18-G3-Me**	Intrinsic antibacterial amphiphilic dendron	Broad activity against resistant Gram-positive and Gram-negative bacteria	Mouse bacteremia, pneumonia, and peritonitis-sepsis models	Systemic treatment	Reduced bacterial burden and improved survival in severe infection models	[45]
**PAMAM/chitosan**	**Cs-PAMAM-MET-NO**	Combined antibiotic and NO delivery	Activity against *E. coli* and *S. aureus*	Rat MRSA-infected wound model	Local wound treatment	Accelerated wound healing	[46]
**PAMAM/chitosan-derived carbon dots**	**CPA-CD-NO**	NO delivery/imaging/antibacterial platform	Reduced *P. aeruginosa* viability and biofilm; fluorescent bacterial imaging	Rat infected wound model	Local wound treatment	Eradicated severe infection and inflammation	[47]
**PAMAM/chitosan**	**CS-PAMAM-Dns**	Glutathione-responsive SO_2_ delivery	Activity against *S. aureus*, MRSA, *E. coli*, and *P. aeruginosa*	*C. elegans* infected with MRSA	Treatment in infected nematodes	Improved survival of infected *C. elegans*	[48]
**PAMAM**	**PAMAM/SDZ**	Non-covalent drug carrier	Sulfadiazine encapsulation	Rat pharmacokinetic/biodistribution study	Oral or i.v. administration	Increased plasma and tissue delivery after intravenous administration; no in vivo anti-infective efficacy model reported	[49]
**PAMAM**	PTM-loaded **PLGA/PAMAM** nanoparticles	Antibiotic carrier	Platensimycin encapsulation	Mouse MRSA peritonitis model	I.p. administration	Full survival of treated infected mice, unlike free platensimycin	[50]
**PAMAM**	**PAMAM/RIF** microspheres	Pulmonary drug delivery	Rifampicin encapsulation in inhalable microspheres	Healthy rat pulmonary delivery model	Intratracheal administration	Altered rifampicin pharmacokinetics after pulmonary delivery; no in vivo infection efficacy reported	[53]
**PAMAM/biomimetic vesicles**	MPN-loaded PAMAM in platelet membrane nanovesicles	Antibiotic adjuvant/pH-responsive delivery	**PAMAM-G5-NH_2_** inhibits NDM activity; meropenem protection/delivery	Zebrafish larvae infection foci; mouse *E. coli* pneumonia and muscle sepsis	I.v. administration	Accumulation at infection sites and protection of infected mice from death	[54]
**PAMAM**	**PAMAM/DMADDM** coating	Dental implant coating/antibacterial delivery	Antimicrobial activity against *C. albicans* and *S. aureus* biofilms	Rat peri-implantitis model	Coated titanium implant	Reduced microbial development and promoted osseointegration	[55]
**PAMAM**	**PAMAM-G4.5-CO_2_H/HNK**	Antibacterial and remineralizing drug carrier	Honokiol delivery against cariogenic bacteria	Rat dental caries model	Topical oral treatment	Reduced caries lesions, decreased *S. mutans*, and promoted enamel remineralization	[56]
**PAMAM**	PAMAM-based Mino/ALA nanocarrier	Dual pH/lipase-responsive drug delivery	Biofilm disruption and antioxidant/antibacterial delivery	Diabetic rat periodontitis model	Local periodontal treatment	Suppressed dental plaque biofilms and periodontal bone loss	[57]
**PAMAM**	**PAMAM-G3.5-AMP/EG**	Antimicrobial peptide-functionalized carrier	High bactericidal efficacy and biofilm disintegration	Rat dentin caries model with oral *S. mutans* infection	Topical dental treatment	Reduced salivary *S. mutans* and increased molar mineral density	[58]
**PAMAM hydrogel**	PAMAM/oxidized dextran/AgNP hydrogel	pH-responsive antibacterial hydrogel	Broad-spectrum antibacterial activity from AgNPs and cationic PAMAM	Mouse *S. aureus* skin infection model	Local subcutaneous hydrogel injection followed by bacterial inoculation at the gel site	Lower bacterial burden than untreated or commercial Ag gel groups	[60]
**PAMAM dendron/collagen**	**ZnO-TES-PAMAM-G3-NP**-collagen scaffold	Antibacterial regenerative scaffold	Antibacterial activity of metal oxide-dendrimer collagen scaffolds	Rat non-infected excisional wound healing model	Local scaffold application	Accelerated wound closure and skin regeneration; no infected in vivo efficacy model	[61]
**PAMAM dendron**	Amphiphilic PAMAM dendron/PNA	Carrier for antisense nucleic acid mimics	Delivery of antisense PNA targeting essential *E. coli* gene and antibacterial activity of the carrier/PNA system	*G. mellonella* larvae toxicity model	Intra-hemocoelic injection	No significant toxicity; no infected in vivo efficacy model reported	[62]
**Poly(L-Lysine)**	**BRI-2999**/**SPL299**	Topical antiviral microbicide	HSV-2 inhibition	Mouse genital HSV-2 infection model	Vaginal topical application	Protection when applied before viral exposure	[66]
**Poly(L-Lysine)**	**SPL7013**/astodrimer	Antiviral and antimicrobial topical dendrimer	Broad antiviral and antibacterial activity; clinical development mainly for bacterial vaginosis and viral infections	Mouse, guinea pig, macaque, rabbit, and human studies	Topical vaginal/rectal/penile gel; nasal spray in SARS-CoV-2 studies	Demonstrated efficacy in bacterial vaginosis trials; antiviral activity shown in multiple preclinical and clinical settings	[67,68,69,70,71,72,73,74,75,76,77,78,79,80,81,82,83,84,85,86,87,88]
**Poly(L-Lysine)**	**PO-Lys-G3-Man@PP-IX-Ga**	Macrophage-targeted antibacterial nanostructure	Intracellular antibacterial activity with gallium and photodynamic effect	Mouse intracellular *S. aureus* infection model	I.v. administration	PG3M@GaPP 50 mg.kg^−1^ achieved 100% survival, weight recovery, reduced organ bacterial burden, and limited relapse after treatment cessation	[89]
**Poly(L-Lysine) nanogel**	**CMPs**	CO-releasing immunomodulatory antibacterial nanomedicine	Activity against *S. aureus*, *C. rodentium*, and *E. coli*	Mouse DSS-induced colitis with bacterial infection	Rectal administration	Reduced pathogens, inflammation, ROS, and remodeled gut microbiota	[90]
**Poly(L-Lysine) nanogel**	**CP-SAP**	Photodynamic antibacterial and remineralizing nanogel	Activity against *S. mutans*, *S. sanguis*, and *C. albicans*	Rat dental caries model	Topical dental application plus laser irradiation	Reduced caries severity and increased enamel density	[91]
**Lysine dendron**	**Ce6&CORM@FADP**	Photodynamic and CO gas antibacterial therapy	Synergistic killing and biofilm ablation against *S. aureus* and *E. coli*	Mouse infected wound and catheter biofilm models	Local treatment plus NIR irradiation	Eradicated bacterial burden and biofilm in vivo	[92]
**Lysine dendrimer hydrogel**	**Lys-G3-PEG2000/OCMC** hydrogel	Antimicrobial adhesive hydrogel	Antimicrobial activity from residual amines	Rat liver hemorrhage and skin wound models	Local hydrogel application	Rapid hemostasis, wound closure, and anti-infection effect	[93]
**Lysine metallodendrimer**	Zn–lysine dendrimer/TSP hydrogel	Antibacterial wound-healing hydrogel	Activity against *P. acnes*, *S. epidermidis*, *S. aureus*, *E. coli*, and *P. aeruginosa*	Rat excision wound model	Topical hydrogel application	Improved re-epithelialization and wound contraction; no infected in vivo model reported	[94]
**Peptide dendron**	**DP-6/GFX**	Ocular antibiotic carrier	Enhanced gatifloxacin solubility/permeability	Rabbit ocular pharmacokinetic and tolerability model	Ocular administration	Increased ocular tissue levels compared with free gatifloxacin; no infected ocular model reported	[97]
**Peptide dendrimer**	**TNS18**	Intrinsic antibacterial peptide dendrimer	Activity against MDR *E. coli* and other bacteria	Mouse MDR *E. coli* peritonitis model	I.p. administration	100% survival, outperforming ceftriaxone and imipenem in this model	[98]
**Peptide dendrimer**	**T7**	Intrinsic antibacterial peptide dendrimer	Activity against *P. aeruginosa* and *K. pneumoniae* strains with low hemolysis	Mouse MDR *A. baumannii* infection model	I.p. administration	Improved survival compared with inactive G3KL	[100]
**Peptide dendrimers**	**TNS18** and **G3KL**	Antibacterial peptide dendrimers	Activity against *P. aeruginosa* strains	*G. mellonella* infection model	Larval treatment	No protection despite in vitro activity	[101]
**Peptide dendron**	**C16-3RP**	Self-assembled antibacterial aggregates	Broad activity against Gram-positive and Gram-negative bacteria	Mouse *E. coli* acute peritonitis model	I.p. administration	Reduced bacterial burden and inflammatory cytokines	[102]
**Peptide dendron**	**C16-2RP-PEG8/C16-3RP-PEG8**	Self-assembled antibacterial aggregates	Activity against MDR Gram-negative bacteria	Mouse MDR *E. coli* peritonitis-sepsis model	I.p. administration	C16-2RP-PEG8 reduced bacterial burden in major organs; C16-3RP-PEG8 was inactive in vivo despite in vitro activity	[103]
**Peptide dendron-PNA**	**(Goc)_8_-DAB-PNA**	Antisense antibacterial dendron conjugate	PNA targeting *acpP* mRNA in Gram-negative bacteria	Neutropenic mouse peritonitis model with MDR *E. coli*	I.p. administration; i.v. used for tolerance/biodistribution	Match PNA significantly reduced infection burden compared with mismatch PNA	[104]
**Dendritic peptide**	**R2(R2)KW4**	Membrane-disruptive antimicrobial peptide	Activity against Gram-positive and Gram-negative bacteria	Mouse *E. coli* pulmonary infection model	I.p. administration	Reduced pulmonary inflammatory infiltration and liver hemorrhage compared with polymyxin B	[105]
**Carbosilane**	**CSi-6** trisaccharide dendrimer	Shiga toxin neutralization	Shiga toxin neutralization	Mouse *E. coli* O157:H7 infection model	I.v. administration	Protected mice from fatal infection and symptoms	[107]
**PPH**	**PPH-G0b**	Intrinsic antimycobacterial dendrimer	Activity against *M. tuberculosis* strains and *M. bovis* BCG	Mouse *M. tuberculosis* H37Ra infection model	Oral administration	Reduced lung bacterial counts more efficiently than reference drugs in the reported model	[110]
**PPH**	**PPH-G0b** + antibiotics	Antibiotic potentiator	Potentiation of rifampicin, bedaquiline, linezolid, and clofazimine against drug-resistant mycobacteria	Murine neutropenic bacteremia model	Oral administration	Combination treatments reduced bacterial counts in organs more than individual treatments	[111]
**PPH**	**PPH-G0Me** ± gentamicin	Intrinsic antibacterial/antibiotic potentiator	Activity against *S. aureus* and *Enterococcus* species	Mouse gentamicin-resistant *S. aureus* skin infection model	Topical skin application	Combination with gentamicin was more effective than either component alone	[112]
**Polyglycerol/chitosan**	**PGL-Ch-B** membrane	Antibacterial wound-healing membrane	Suppressed *S. aureus* and *P. aeruginosa* proliferation	Rat wound model	Wound membrane coating	Complete healing by Day 30 with membrane degradation, no infected in vivo model reported	[117]
**Polyglycerol dendron**	**Gly-G1-C12**	Intrinsic antibacterial cationic surfactant	Activity against MRSA and *E. coli*, including biofilms	Mouse MRSA-infected wound model	Local wound treatment	Accelerated wound closure and collagen recovery	[118]
**Polyester dendrimer**	**mPEG-b-PCL/G1-PEA** vancomycin nanovesicles	Antibiotic delivery	Lower MIC against MSSA and MRSA than free vancomycin	Mouse MRSA skin infection model	Local intradermal administration at infection site	Reduced MRSA load more than free vancomycin	[120]
**Polyester DLD**	**G6-DLD-20k** hydrogel	Antibiotic-free antibacterial hydrogel coating	Broad antibacterial activity against multiple strains, including MRSA	Mouse *S. aureus*-infected suture model	In situ hydrogel coating	Reduced bacterial load post-infection	[121]
**Citric acid DLD**	**G5-DLD-600-CFTX**	Antibiotic carrier	Cefotaxime encapsulation and antibacterial activity	Rat pharmacokinetic study	Intramuscular administration	Improved pharmacokinetic profile; no in vivo antimicrobial efficacy test	[122]
**Cholic acid DLD**	**CA-DLD-2k**	Intrinsic antibacterial amphiphilic dendrimer	Activity against *S. aureus* and *E. coli*	Mouse *S. aureus*-infected wound model	Intrawound injection	More effective than penicillin G in this model and promoted wound healing	[123]
**Poly(urea)**	**G4-PURE-OEI**	Broad-spectrum antibacterial and antifungal dendrimer	Activity against Gram-positive, Gram-negative bacteria and *Candida* spp.	*G. mellonella* infected with *S. aureus* or *P. aeruginosa*	Larval treatment	Improved larvae survival	[126]

## Data Availability

No new data were created or analyzed in this study. Data sharing is not applicable to this article.

## Abbreviations

The following abbreviations are used in this manuscript:

ALA	α-Lipoic acid
AMP	Antimicrobial peptide
AMR	Antimicrobial resistance
Arg8	Oligoarginine peptide
AZM	Azithromycin
BCG	Bacille Calmet–Guérin
BN	Borneol
BV	Bacterial vaginosis
CA	*Cis*-aconit or cholic acid
CD	Cyclodextrin or carbon dots
CFTX	Cefotaxime sodium
Ch	*O*-carboxymethylated chitosan
CMP	CORM-mannose dendritic polymer
CO	Carbon monoxide
CORM	CO-releasing molecule
COVID	Coronavirus disease
CS	Chitosan
CSi	Carbosilane dendrimer
CuAAC	Copper-catalyzed azide-alkyne cycloaddition
DA	2,3-Dimethyl maleic anhydride
DAB	Diaminobutanoic acid
DEX	Dexamethasone
DLD	Dendritic–linear-dendritic hybrid
DLS	Dynamic light scattering
DMADDM	Dimethylaminododecyl methacrylate
Dns	2,4-dinitrobenzenesulfonyl chloride
DOP	Dopamine
DSS	Dextran sulfate sodium
EDC	1-Ethyl-3-(3-dimethylaminopropyl)carbodiimide
EG	Epigallocatechin gallate
ETB	Ethambutol
FADP	Fluorinated amphiphilic dendritic peptide
FITC	Fluorescein isothiocyanate
Gbu	Guanidinobutanoic acid
GFX	Gatifloxacin
Ghp	Guanidinoheptanoic acid
Ghx	Guanidinohexanoic acid
Goc	Guanidinooctanoic acid
Gpn	Guanidinopentanoic acid
HIV	Human immunodeficiency virus
HNK	Honokiol
HPV	Human papillomavirus
HSV	Herpes simplex virus
I.p.	Intraperitoneal
I.v.	Intravenous
LNA	Locked nucleic acid
Lys	L-Lysine
Man	Mannose
MAO	Micro-arc oxidation
MDR	Multidrug resistance
MET	Methicillin
MIC	Minimum inhibitory concentration
Mino	Minocycline hydrochloride
MMC	Minimal microbicidal concentration
MO	Metal oxide
MPN	Meropenem
MRSA	Methicillin-resistant *Staphylococcus aureus*
NAC	N-acetyl cysteine
NAM	Nucleic acid mimic
NDM	New Delhi metallo-β-lactamase
NHS	*N*-hydroxysuccinimide
NIR	Near-infrared
NO	Nitric oxide
NP	Nanoparticle
OCMC	Oxidized carboxymethyl cellulose
OEI	Oligoethyleneimine
PAMAM	Poly(amidoamine)
PBS	Phosphate-buffered saline
PCL	Poly(ε-caprolactone)
PDN	Poly(L-Lysine) dendrimer-based nanogel
PED	Polyester dendrimer
PEG	Poly(ethylene glycol)
PGL	Polyglycerol
P-Lys	Poly(L-Lysine)
PMV	Platelet membrane nanovesicle
PNA	Peptide nucleic acid
POSS	Polyhedral oligomeric silsesquioxane
PPH	Poly(phosphorhydrazone)
PP-IX	Protoporphyrin IX
PTM	Platensimycin
PURE	Poly(urea)
RIF	Rifampicin
ROS	Reactive oxygen species
SA	Succinic anhydride
SAP	Saliva-acquired peptide
SARS-CoV	Severe acute respiratory syndrome coronavirus
SDZ	Sulfadiazine
SHIV	Simian-human immunodeficiency virus
SM	Streptomycin
SPDP	*N*-succinimidyl 3-(2-thiopyridyl)propionate
SPL7013	Astodrimer sodium, VivaGel
STI	Sexually transmitted infection
Stx	Shiga toxin
TDZ	Tedizolid
TEM	Transmission electron microscopy
TES	Triethoxysilyl
TSP	Tamarind seed polysaccharide
